# MoCaNLO: a Monte Carlo integrator for NLO calculations

**DOI:** 10.1140/epjc/s10052-026-15752-7

**Published:** 2026-08-27

**Authors:** A. Denner, D. Lombardi, S. Lopez Portillo Chavez, M. Pellen, G. Pelliccioli

**Affiliations:** 1 Institut für Theoretische Physik und Astrophysik, Universität Würzburg, Emil-Hilb-Weg 22, 97074 Würzburg, Germany; 2 Dipartimento di Fisica, Università di Torino and INFN, Sezione di Torino, Via P. Giuria 1, 10125 Turin, Italy; 3 Physikalisches Institut, Albert-Ludwigs-Universität Freiburg, Hermann-Herder-Str. 3, 79104 Freiburg, Germany; 4 Dipartimento di Fisica “Giuseppe Occhialini”, Università degli Studi di Milano-Bicocca and INFN, Sezione di Milano-Bicocca, Piazza della Scienza 3, 20126 Milan, Italy

## Abstract

We present the Monte Carlo integration code MoCaNLO, which computes cross sections and distributions for processes at high-energy colliders like the LHC at leading and next-to-leading order (NLO) in the strong and electroweak couplings. It relies on the Recola package for the calculation of matrix elements and uses Catani–Seymour dipole subtraction for the treatment of infrared singularities. It has been used for several cutting-edge calculations of NLO QCD and electroweak corrections over the last years, such as NLO QCD corrections to off-shell top–antitop-quark production in association with a pair of bottom quarks and NLO electroweak corrections to vector-boson scattering processes.

## Introduction

The analysis of particle-physics scattering experiments relies heavily on simulations based on first-principles calculations within the framework of the Standard Model (SM) of particle physics. To this end, event generators like MadGraph5_aMC@NLO [1], Powheg-Box [2], Sherpa [3], Herwig [4], or Pythia [5] are commonly used, either as stand-alone tools or in combination, to simulate complete events from the hard scattering to hadronisation. On the other hand, for processes with many particles in the final state, these event generators are not yet able to incorporate state-of-the-art predictions as obtained in fixed-order calculations. This kind of predictions can be accomplished via dedicated Monte Carlo (MC) integration programs that often provide results in terms of weighted events. One such program is MoCaNLO, which is described in this manual.

MoCaNLO (MOnte CArlo at NLO accuracy) is a flexible multi-purpose MC integration program. It computes integrated cross sections and differential distributions for arbitrary processes at the LHC with both NLO QCD and electroweak (EW) accuracy in the SM. Thereby, full off-shell effects and spin correlations are taken into account. Besides, NLO corrections to processes at lepton–lepton and lepton–hadron colliders, as well as ultra-peripheral collisions of photons from hadrons or heavy ions are also supported.

The phase-space integration uses an elaborated multi-channel importance sampling along the lines of Refs. [6–9]. The required tree-level and one-loop matrix elements are provided by the matrix-element generator Recola [10, 11], which relies on the Collier library [12] to numerically evaluate the one-loop scalar and tensor integrals. The subtraction of the infrared (IR) divergences appearing in the real and virtual corrections has been automated based on the Catani–Seymour dipole formalism for both QCD and QED [13–18]. For the separation of photons and jets in the final state both Frixione isolation [19] and the photon-to-jet fragmentation function [20–22] are available. The IR singularities arising from the splitting of virtual photons into quark–antiquark pairs are regularised using the photon-to-jet conversion function [23]. Unstable particles are treated in the complex-mass scheme [7, 24–26]. Alternatively, the pole approximation, which is based on the pole expansion [26–28], can be used for the virtual corrections following the strategy of Ref. [29]. The corresponding non-factorisable virtual corrections are implemented according to Refs. [30–32]. For a class of polarised and unpolarised processes, the pole approximation can be employed for all contributions to NLO cross sections, while ensuring that all matrix elements are evaluated in the same Lorentz reference frame. For leading-order (LO) processes, MoCaNLO can deal with arbitrary particles in the final state. However, for NLO calculations external particles that emit real radiation, i.e. photons or gluons, have to be massless. While the light quarks are massless throughout, the bottom quark and the τ lepton can be massive, if they do not appear as radiating external particles. Masses for the electron and the muon only play a role in the regularisation of collinear singularities for initial-state leptons or as input for the lepton structure functions, i.e. they are exclusively relevant for processes at lepton colliders.

MoCaNLO has demonstrated its ability to compute NLO corrections for high-multiplicity processes up to 2→8. Thus, it has been used to calculate NLO QCD and EW corrections to top-pair and associated top-pair production [33–41], to the associated production of single bosons [23, 42], to the production of gauge-boson pairs [43, 44], to the production of single Higgs bosons and Higgs-boson pairs via vector-boson fusion [45, 46], to triple-vector-boson production [47, 48], to vector-boson scattering (VBS) processes and their irreducible background [49–57], and to single-top processes [58]. It has also been employed to evaluate NLO QCD and EW corrections for processes involving polarised vector bosons [59–69].

This manual is structured as follows: The installation of the code is described in Sect. 2, while its basic use is illustrated in Sect. 3. The following sections discuss the input cards needed to run the program, namely the run_card.xml in Sect. 4, the proc_card.xml in Sect. 5, the param_card.xml in Sect. 6, the cut_card.xml in Sect. 7, and the plot_card.xml in Sect. 8. User-defined features are described in Sect. 9. After the conclusions, Sect. 10, we provide a list of validated processes in Appendix A.

## Installation and compilation

MoCaNLO can be downloaded from the git repository

https://mocanlo.gitlab.io

Unpacking the tarball creates five directories. The folder MoCaNLO contains the actual code, while the directory Tests offers some test runs with corresponding sample input and output. In the folder validated_processes, which is referred to as /path/to/processes in the following, further example processes can be found. The manual directory contains the PDF file for the code documentation. The directory card_generator contains the independent package MCG, which may be used to create input cards for MoCaNLO.

The MoCaNLO package consists of the MC code itself and the additional program MCG. The MC code is independent of the program MCG, whose use is optional. For details about the usage and compilation of MCG, we refer to the file card_generator/mcg/README.md.

The MoCaNLO folder, which is referred to as /path/to/mo canlo, has the following subdirectory structure:

**Figure:**
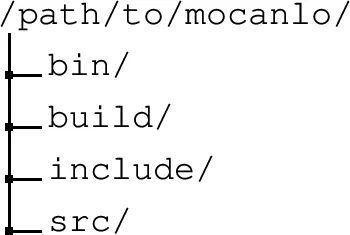

The subdirectory bin contains binaries upon successful compilation and scripts for collecting results and generating plots. The build directory contains compiled object (.o) and Fortran module files (.mod) upon compilation. The include directory holds include files containing several preprocessor switches. Finally, the source code of MoCaNLO as well as some supplementary tools are placed in the subdirectory src.

**Prerequisites and external packages**

**Table 1:** Software requirements and external packages needed to run MoCaNLO

Software requirements	
	Recommended
gfortran	≥8.3.0
CMake	≥3.13.4
gnuplot	≥5.4.1

External packages	
	Recommended
LHAPDF	≥6.0.5
Recola	≥1.5
Collier	≥1.2.9

The software requirements for the MC code are listed in Table 1. Additionally, some external packages are needed. Each package has to be compiled separately and its installation location has to be properly added to the compilation script compile_mocanlo available in the /path/to/mocan lo folder. They are:

- **LHAPDF**: MoCaNLO is linked to LHAPDF [70] for the evaluation of parton distribution functions (PDFs). For a successful build, compile LHAPDF dynamically (https://lhapdf.hepforge.org) and set LHAPDF_PATH in the script compile_mocanlo to the location of the LHAPDF installation, i.e. the folder with the subdirectories bin, include, lib (containing libLHAPDF.so), and share. Set the LHAPATH environment variable to the path where PDF sets are installed, e.g. execute

**Figure:**
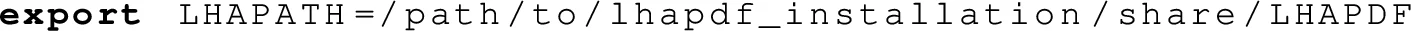

if these are stored in /path/to/lhapdf_installation /share/LHAPDF.
- Recola **/** Collier : MoCaNLO relies on Recola  [10, 11] and Collier  [12] for the matrix-element computation and loop-integral reduction and evaluation. The two packages have to be compiled separately from MoCaNLO. Instructions and downloads can be found at https://collier.hepforge.org/index.html and https://recola.gitlab.io/index.html. After a successful ins-tallation, the path variables COLLIER_ROOT and RECOLA_ ROOT in the script compile_mocanlo have to be set to the location of the compiled Collier and Recola libraries, respectively.

**Figure:**
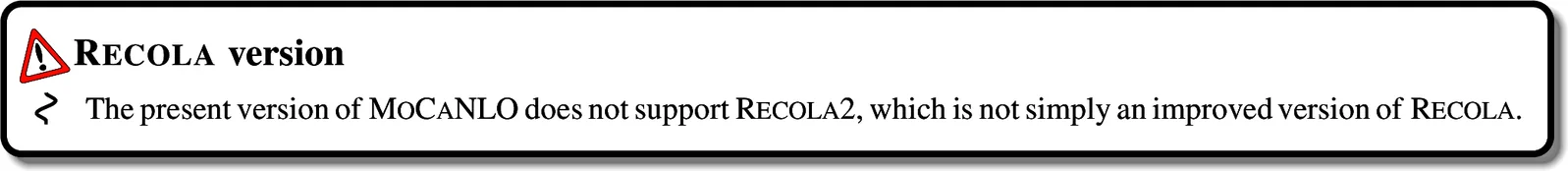
- **gamma-UPC** (optional): For photon–photon ultra-perip-heral collisions (UPC), MoCaNLO makes use of the gamma-UPC library [71] for the photon PDF. Instructions and downloads can be found at https://www.lpthe.jussieu.fr/~hshao/gammaupc.html. After a successful installation, the path variable UPCDIR in the script compile_mocanlo has to be set to the location of the compiled gamma-UPC library. In addition, in the script compile_mocanlo, cmake must be executed with the option-DUPC=ON.

**Compilation** To build MoCaNLO, simply execute the script compile_mocanlo as:

**Figure:**
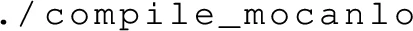

The main MoCaNLO executable mocanlo appears in /path /to/mocanlo/bin upon successful compilation of the sour-ce. The script binds the different libraries to mocanlo using CMake. To make use of all scripts and executable (see description later), add the MoCaNLO bin directory to PATH, by executing

**Figure:**
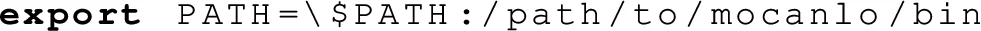

**Tests** The correct functionality of the code can be verified upon executing the test runs in the directory Tests. Each subdirectory *test* corresponds to a physical process and defines a test based on a single partonic subprocess. Available test folders are: sl-vbs ($\text {p} \text {p} \rightarrow \mu ^+{\nu }_\mu \text {j} \text {j} \text {j} \text {j}$), ttxz ($\text {p} \text {p} \rightarrow \text {e} ^+\nu _\text {e} \mu ^-\bar{\nu }_\mu \tau ^+\tau ^-\text {j} _{\text {b}} \text {j} _{\text {b}}$), tzj ($\text {p} \text {p} \rightarrow \text {e} ^+\text {e} ^-\mu ^+\nu _\mu \text {j} _{\text {b}}\text {j}$), vbs-zz ($\text {p} \text {p} \rightarrow \text {e} ^+\text {e} ^-\mu ^+\mu ^-\!\text {j} \text {j}$), and wz-pol ($\text {p} \text {p} \rightarrow \text {e} ^+\nu _\text {e} \mu ^+\mu ^-$ with polarised $\text {Z}$ and $\text {W}$ $^+$ bosons), where $\text {j} _\text {b}$ denotes bottom jets. The script Tests/check_process.sh, which must be executed inside Tests/, takes one of the test directories *test* as its argument

**Figure:**
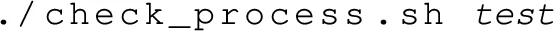

and runs MoCaNLO using *test*/cards and *test*/inputs as input. The standard output of MoCaNLO is printed to the screen and saved in the file *test*/output_local, which is compared to *test*/output. If the standard output is as expected, the script compares all cross sections and histograms calculated locally, which are written to *test*/*test*/runs/.../run_.../results, to their counterparts found in *test*/results. After each pair of files is compared, the script warns if these differ and offers their location for manual comparison. The test finishes with a summary of failures. If no argument is passed to check_process.sh, all directories are tested.

**Figure:**
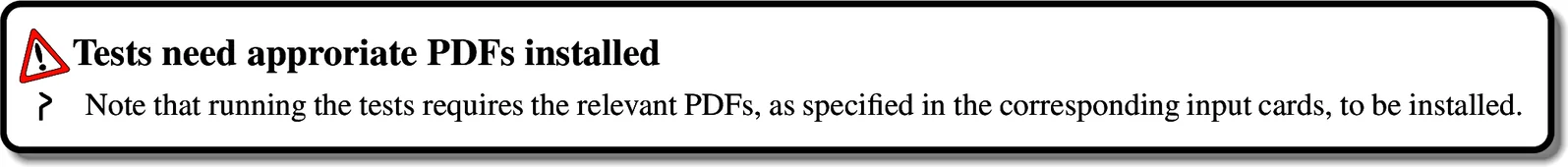

## Getting started

### Citation

If you use MoCaNLO, please cite the present manual. As mentioned above, input cards can be generated for arbitrary processes using MCG. Alternatively, example cards for a large variety of processes are provided. If you happen to use some of the example cards, please also cite the related references as indicated in the README.txt file of each process or as reported in Appendix A.

### Running the example process

This section outlines the procedure for running MC simulations of the process $\text {p} \text {p} \rightarrow \text {e} ^+\nu _\text {e} \text {b} \mu ^-\bar{\nu }_\mu \bar{\text {b}}$, which is one of the examples provided within the MoCaNLO package and can be found under /path/to/processes/ttbar/pp_tt/. Before any MC run is submitted, the process directory pp_epvemumvmxbbx_qcd contains only one folder named cards, with four XML files that define subprocesses, standard model and MC parameters, cuts to apply, and plots to create: proc_card.xml, param_card.xml, cut_card.xml, and plot_card.xml, explained in detail in Sect. 4.

One additional XML file, i.e. run_card.xml, defines MC runs for individual contributions of partonic subprocesses: In our example process one finds Born, virtual, real, and integrated-dipole contributions (see Sect. 5.1) labelled by integers.

**Figure:**
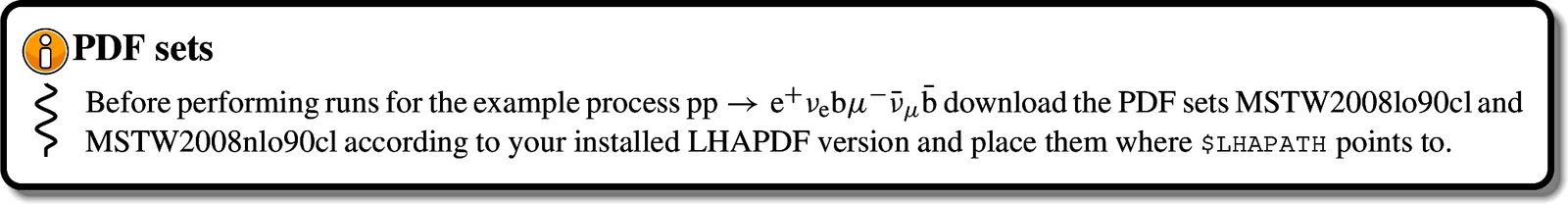

### Running a single contribution to a subprocess

The run with id="1", as displayed in Listing 1, specifies the Born contribution of the gluon–gluon-induced subprocess $\text {g} \text {g} \rightarrow \text {e} ^+\nu _\text {e} \text {b} \mu ^-\bar{\nu }_\mu \bar{\text {b}}$ (defined in proc_card.xml). This contribution constitutes the Born part of the NLO cross section.

**Figure:**
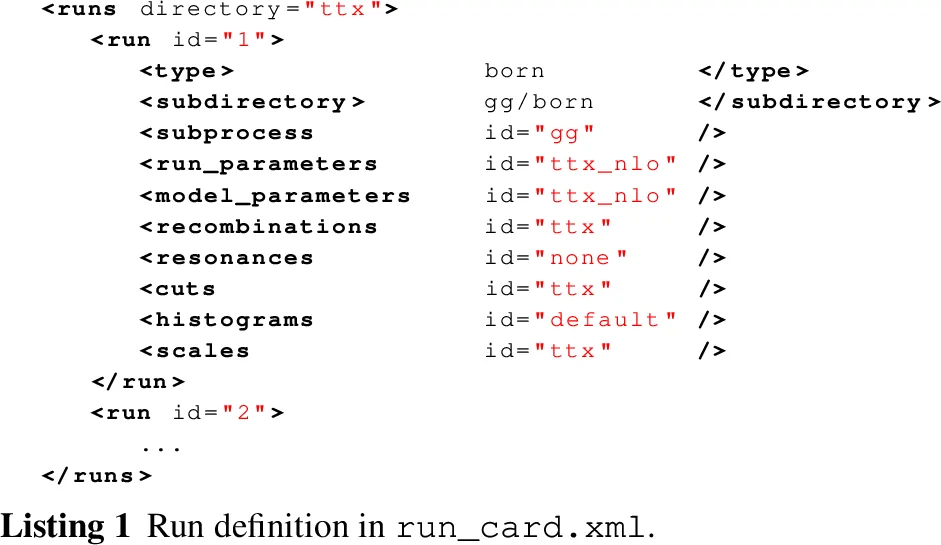

To perform the run, execute MoCaNLO with the (relative or absolute) path to the process as first and the desired run ID as second command-line argument:

**Figure:**
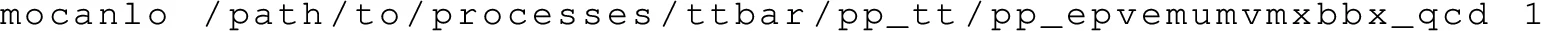

In the following, we make use of  to ease notations. The run prints information about the initialisation and the input parameters, and, once Recola is initialised, it outputs a list of parameters as being used for the evaluation of the matrix elements. After the initialisation phase, the MC run begins, printing intermediate result summaries once 1000 accepted events have been reached and at increasing intervals thereafter. Each time an intermediate result is printed to the terminal, this result as well as all plots defined in plot_card.xml are dumped to disc. Thus, the process may be killed with Ctrl+C without loosing the obtained results.

The run creates a new subdirectory structure in the process directory, e.g.:

**Figure:**
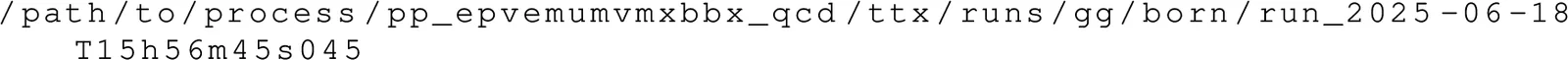

where ttx is defined by  in Listing 1, grouping several runs into a *runs set*. The subdirectory gg/born/ in runs/ is set as the run’s output directory by $\mathtt {<}  \texttt {subdirectory}\mathtt {>}$ in the run definition. Each run with the same ID creates a subdirectory in the latter including a timestamp of the form run_YYYY-MM-DDTHHhMMmSSsSSS, which contains the cross sections and plots in its result subdirectory, as well as information about the initialisation and the run phase of the MC in its data subdirectory. The example run is setup in such a way that it is computed for 3 variations of the renormalisation and factorisation scale (specified in param_card.xml in Listing 16) at the same time by rescaling the matrix elements and the PDFs. To this end, scale factors are applied to the central scales. The first scale factor, which usually equals one, is identified with the central scale, and the corresponding result is displayed as intermediate result on the terminal. The second and third scale factors, for example, correspond to a factor of 0.5 and 2, respectively. When more than one scale factor is used, the subdirectory result contains folders of the type scale_factor_ *i* hosting the results for each scale factor. The resulting directory structure is as follows:

**Figure:**
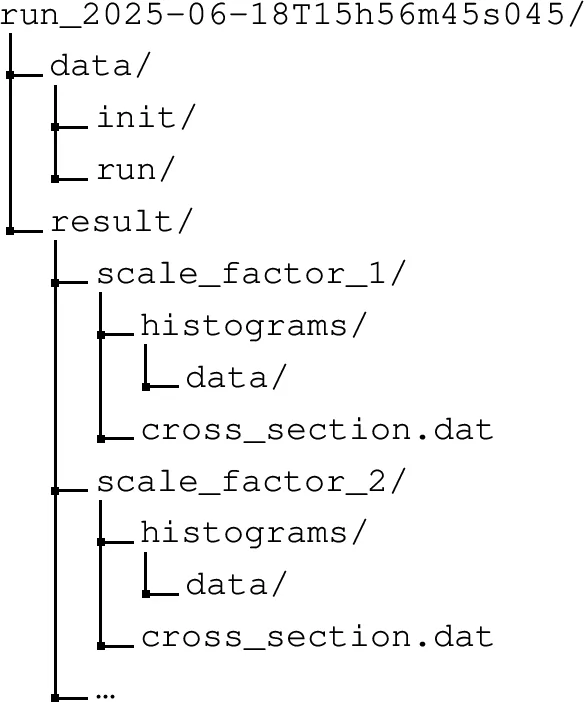

The intermediate result as displayed on the terminal is printed to the file cross_section.dat for each scale factor: At the top of this file, a selection of the most important information is also reported in one line, preceded by a pipe symbol “|”, to facilitate its extraction via the grep command. At the bottom, a summary of occurred errors and exception is printed. The folder histograms/data contains data files for each plot defined in plot_card.xml of the form histogram_ *type*_ *name*.dat, e.g. one has histogram_transverse_momentum_positron.dat for the transverse-momentum distribution of the positron.

In addition, the subdirectory data/init/user_input contains a set of files that reflect the inputs to the run. The file data/init/feynman_diagrams.tex encodes all Feynman diagrams corresponding to the constructed integration channels in LaTeX format. With the command sequence

**Figure:**
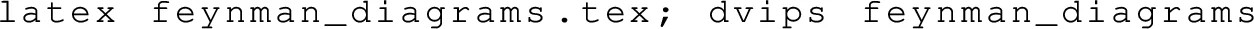

a PS file is generated that shows all these diagrams, provided that the package axodraw [72, 73] is installed.

The subdirectory data/run contains the file feynman_di agrams_survey.tex, which encodes again all diagrams but now in the order of importance for the integration, which is complemented by the information in the file channel_survey.dat. The file integration_stability shows the evolution of the integration error with the number of accepted events. Finally, the file max_weight_event.dat stores information on the event with the largest weight and the file typical_events.dat information on 10 events of typical weight.

**Figure:**
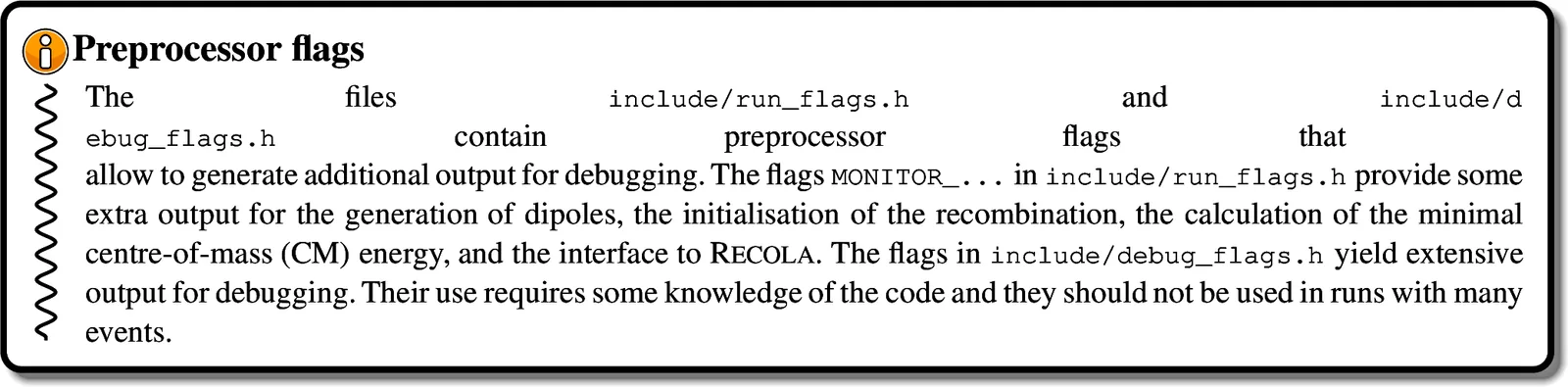

### Averaging of multiple runs

To speed up the integration, runs may be performed in parallel and their results, i.e. integrated cross sections and differential distributions, averaged. For these runs to be independent, different seeds for the random number generator must be used. An optional third argument to mocanlo allows to pass a seed to the MC, while without a third argument, a fixed standard seed is chosen. The Linux shell Bash provides the function $RANDOM returning a pseudo-random integer on each invocation. It may be used to provide seeds to MoCaNLO to- perform independent MC runs by executing, possibly more than once

**Figure:**
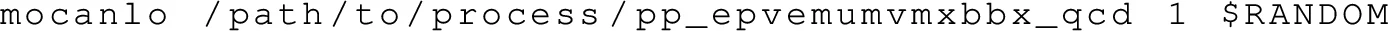

which is equivalent to

**Figure:**
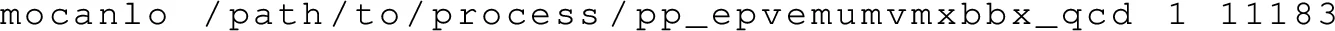

if $RANDOM returns the random number 11183.

When a seed is passed to MoCaNLO, it is appended to the name of the created run folder as run_YYYY-MM-DDTHHhMMmSSsSSS_ *seed*, e.g. :

**Figure:**
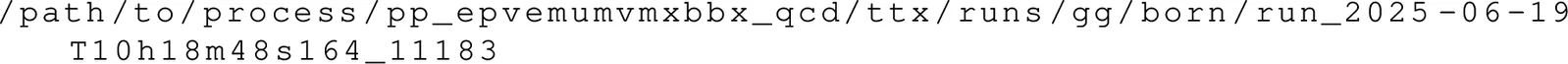

with the random seed 11183.

MoCaNLO comes with a tool that averages runs, i.e. integrated cross sections and differential distributions: the script average_runs (to be found in the bin folder like all scripts described in the following) averages all runs in any subfolder of a runs set directory passed as a first argument to the binary:

**Figure:**
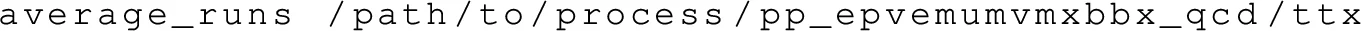

It creates the folder average in the directory hosting the run folders, which contains a subfolder result with the averaged cross section in cross_section.dat and the averaged histograms in the folder histograms/data, individually for every scale factor. When the tool encounters contributions with only one run, it still creates the folder average, but as a symbolic link to the single run directory. For each contribution the script performs (where needed) the average of all runs with different random seeds. If runs with equal random seeds are found, the script stops. In this case the user is required to manually intervene to decide which folders to keep and make sure that only one run per random seed exists before launching the script again. In case a contribution was restarted using the code’s features described in Sect. 6.2.1, only the last run folder that was created is considered. The latter corresponds to the folder whose name contains the additional step *N* identifier with the highest integer *N* within the contribution’s folder. Note that the averaging is computed as if the separate runs were one concatenated MC run. If one wants to only obtain an average of the cross sections (without having to wait for all histograms for all scale factors to be averaged), the average_runs script can also be run with a second optional argument, namely--xs-only, that precisely skips the histogram averaging.

### Performing an NLO computation

The run_card.xml usually defines different runs that contribute to a full NLO calculation of the hadronic process, such as $\text {p} \text {p} \rightarrow \text {e} ^+\nu _\text {e} \text {b} \mu ^-\bar{\nu }_\mu \bar{\text {b}}$. An NLO calculation requires at least one run for Born, virtual corrections, real corrections, and integrated dipole contributions (Sect. 5.1).

For the considered example process, after a run of a few hours one can retrieve the NLO results. Executing

**Figure:**
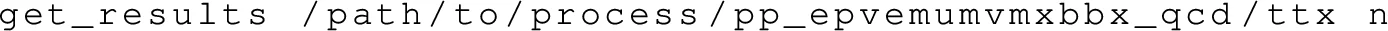

performs the run averaging for the cross sections and prints them for the *n*th scale factor to the terminal. If the last argument is missing, the results for all scale factors are printed.

After averaging the runs as described in Sect. 3.4, different averaged contributions can be merged with the command

**Figure:**
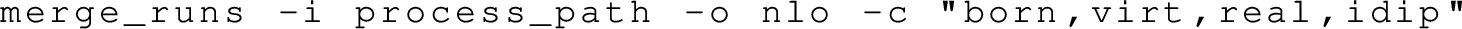

where the argument-i provides the target folder where averaged results for the different contributions can be found, e.g. . The argument-o specifies the name of the output folder in the results directory, which in the example above is located in process_path/results/nlo. Finally,-c contains the list of names of contributions to be merged, enclosed in double quotes and either space or comma separated. Note that the name of contributions in the list must match the names of the folders where the results for the different contributions for each partonic channel are written by the MoCaNLO code, i.e. as specified in the run_card.xml. A summary of how to execute the script can be found by typing

**Figure:**
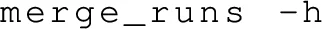

The script sums up all indicated contributions to cross sections and histograms into the subdirectory nlo of results. The structure of the output folder is as follows:

**Figure:**
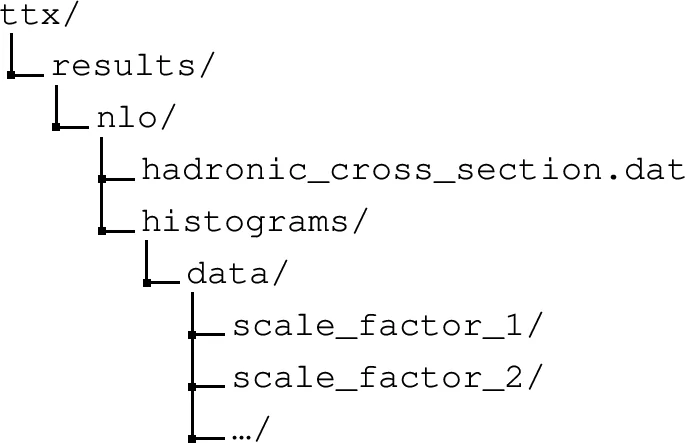

The merged cross sections are listed for all scale factors in the file hadronic_cross_section.dat in the directory nlo. The merged histogram files are written to the directories scale_factor_ *i*.

### Plotting results

MoCaNLO comes with some scripts that allow to plot results using gnuplot based on the histograms obtained with merge_runs. The script create_k-factor_plots produces absolute distributions together with ratios of contributions, create_correction_plots absolute distributions along with relative contributions, and create_scale_enve lope_plots absolute distributions and relative contributions with scale envelopes based on the results in the different scale_factor_ *i* directories. The syntax of these commands reads:

**Figure:**
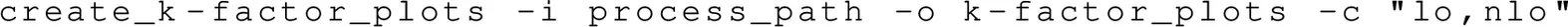

The argument-i provides the runs set folder where merged results for the different contributions are located in the subdirectory results. The argument-o specifies the name of the output folder in the plots directory, which in the example above is located in process_path/plots/k-factor_plo ts. This is also the default, in case the argument-o is omitted. Finally,-c contains the list of names of contributions to be plotted, enclosed in double quotes and either space or comma separated. These contributions have to be created via merge_runs with the corresponding names. Thus, in the example above the subfolders results/lo and results/nlo must exist and contain the relevant information. The first contribution is used as reference for the relative plots.

A folder plots is created in the runs set directory, which contains the subdirectory specified in the plot command. The resulting directory structure is as follows

**Figure:**
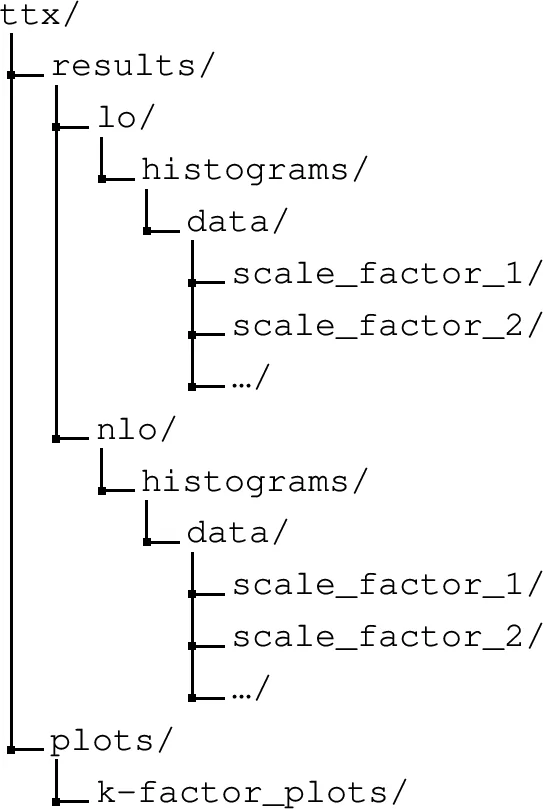

Single EPS and PDF files for the individual histograms are placed in plots/k-factor_plots together with a PDF file named k-factor_plots.pdf that collects all histograms. Each plot shows the different contributions, i.e. lo and nlo in this example, in an upper panel and the contributions divided by the first one in the lower panel, i.e. lo/lo and nlo/lo in the example.

The script create_correction_plots works in the same way, but is meant for displaying relative corrections. If for instance the virtual, real, and integrated dipole corrections have been merged with appropriate executions of the merge_runs script and stored in the folders results/virt, results/real, and results/idip, these could be plotted via

**Figure:**
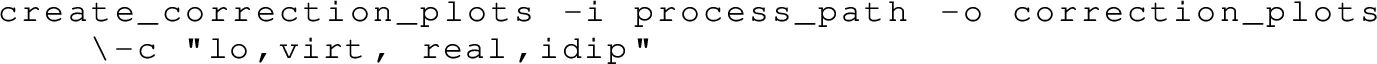

The resulting plots show the absolute contributions lo,virt ,real, and idip in the upper panel and the ratios of virt/lo, real/lo, and idip/lo in the lower panel in per cent. The default output folder, in case the argument-o is omitted, is process_path/plots/correction_plots.

The script create_scale_envelope_plots works as the script create_k-factor_plots, but displays in addition bands for the scale variation obtained from the maxima and minima in the different process_path/results/.../hist ograms/data/scale_factor_ *i* directories. It creates in .../histograms/data a further subdirectory called scale _envelope containing the data for the scale-envelope plots.

**Figure:**
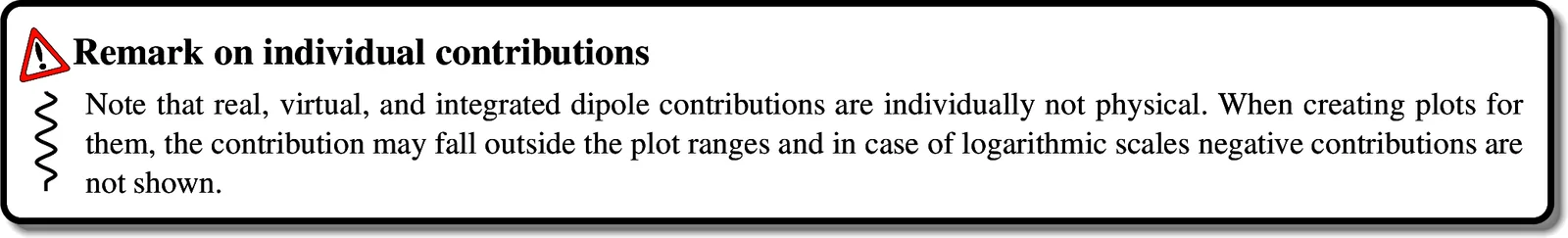

## Setting up a process in the run_card.xml

A run for a given process is fully specified in MoCaNLO by five input cards: run_card.xml, proc_card.xml, param_ card.xml, cut_card.xml, and plot_card.xml (see Table 2). The code expects them to be located in a folder named cards inside the specific process directory:

**Figure:**
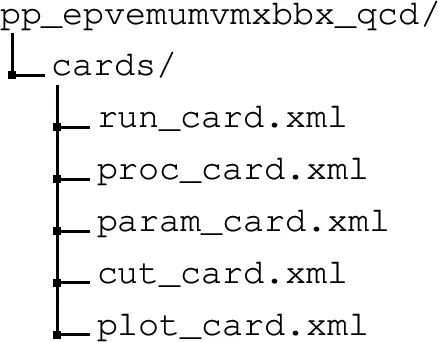

**Table 2:** List of cards for the process definition, together with the different XML sections they contain. The symbol ✓ marks the sections that must be linked to a process definition in the run_card.xml, while all remaining ones are optional. The rightmost column contains the section of this manual that describes each section

Card	XML section(s)		Description	
run_card.xml	$\mathtt {<} \texttt {run}\mathtt {>}$	–	Definition of MC runs using links to other cards	
proc_card.xml	$\mathtt {<} \texttt {subprocess}\mathtt {>}$	✓	List of partonic subprocesses and their coupling order	Section 5.1
	$\mathtt {<} \texttt {resonances}\mathtt {>}$		Specification of resonances restricting the generated phase-space mappings and matrix-element contributions	Section 5.2
	$\mathtt {<} \texttt {on\_shell\_projection}\mathtt {>}$		Details of the pole approximation and its on-shell projection	Section 5.2
param_card.xml	$\mathtt {<} \texttt {model\_parameters}\mathtt {>}$	✓	Parameters of the particle model such as masses and widths	Section 6.1
	$\mathtt {<} \texttt {run\_parameters}\mathtt {>}$	✓	Various MC-run parameters	Section 6.2
	$\mathtt {<} \texttt {weight\_opts}\mathtt {>}$		Tuning of the multi-channel weight optimisation	Section 6.3
cut_card.xml	$\mathtt {<} \texttt {recombinations}\mathtt {>}$		Definitions of recombinations for the jet algorithm	Section 7.1
	$\mathtt {<} \texttt {cuts}\mathtt {>}$		Phase-space cuts	Section 7.3
	$\mathtt {<} \texttt {scales}\mathtt {>}$		Definition of the renormalisation/ factorisation scale	Section 7.4
plot_card.xml	$\mathtt {<} \texttt {histograms}\mathtt {>}$		Histograms	Section 8

The run_card.xml plays the role of the top-level card. In it, all contributions (to be computed as individual runs), which together represent an independent and self-consistent calculation, are grouped by runs sets, as illustrated in Listing 1. In this example, the XML group  defines a runs set ttx, which means that the output of all runs in this group will appear in subdirectories of the folder ttx in the process directory.

Within a runs set, the most elemental part of a calculation is represented by a run, which is fully specified by the tags contained in the XML group , with N an integer number. In our example,  defines the process with ID 1.

**Figure:**
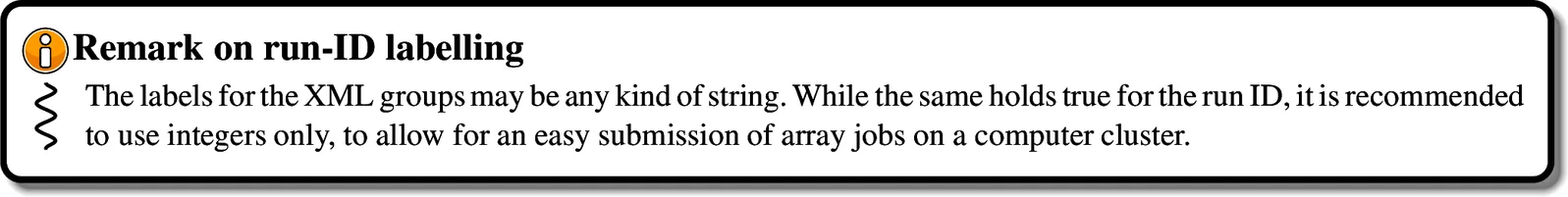

The tags that define a run can be divided in three different classes:

- **basic tags**: This set comprises two tags that should always be specified when defining a process. The tag $\mathtt {<}  \texttt {type}\mathtt {>}$ is used to define the type of contribution to be computed and can take one of the following values: born, real, virt, idip, lpid, vnfc, idpi, and idpk. The output and results of the run are stored in the folder’s runs set in the path given by the tag $\mathtt {<}  \texttt {subdirectory}\mathtt {>}$.
- **linking tags**: These tags are used to link XML sections that are defined in the other four cards. Each of these tags has a one-to-one correspondence with these sections, whose complete list is reported in Table 2. For instance, the tag  in Listing 1 refers to the homonymous XML group in cut_card.xml, which hosts a specific set of cuts. These tags are particularly useful, since they offer the flexibility to use different setups (e.g. parameters, cuts, set of histograms, ...) for different IDs, and simplify switching back and forth between setups. Note that the linking tags need to be specified for every process: the ones that MoCaNLO requires for a consistent run are marked with the symbol ✓ in Table 2. If those are not present, the code will stop with an appropriate message.
- **flag tags**: This set includes only the optional tag $\mathtt {<}  \texttt {osp\_type}\mathtt {>}$, which controls some features of the pole approximation as described in Sect. 5.2.

**Figure:**
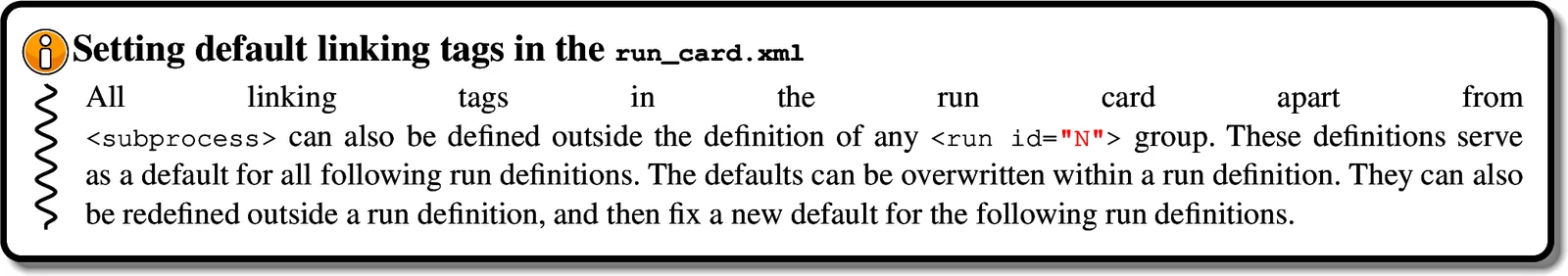

## Definition of the process with the proc_card.xml

The process card (proc_card.xml) contains the list of all partonic processes that can be linked in the run_card.xml to define a process with a given run ID (see Listing 1). This list is enclosed in the XML section $\mathtt {<}  \texttt {subprocesses}\mathtt {>}$. In there, each subprocess contains the information that uniquely defines the $\mathtt {<}  \texttt {run\_type}\mathtt {>}$, i.e. the specific LO or NLO contribution to be computed, as described in Sect. 5.1.

In the process card, intermediate resonances can be specified in the section $\mathtt {<}  \texttt {resonances}\mathtt {>}$ to enable a dedicated phase-space integration and/or the pole-approximation algorithm, which serves as a basis for the definition of polarised cross sections (see Sect. 5.2). The section $\mathtt {<}  \texttt {on\_shell\_projection}\mathtt {>}$ allows to control some features of the on-shell projection used to compute it.

### Partonic process specification

A subprocess definition is enclosed in an XML $\mathtt {<}  \texttt {subprocess}\mathtt {>}$ block identified by a unique subprocess ID (see Listing 2), which is used by the run_card.xml to refer to it. Its definition comprises two parts: the partonic process ($\mathtt {<}  \texttt {partonic\_process}\mathtt {>}$), which describes the lowest-order contribution to the considered channel, and the real process ($\mathtt {<}  \texttt {real\_process}\mathtt {>}$), which specifies the contribution with one additional final-state particle. The same subprocess ID can be referred to in the run_card.xml by multiple run IDs with different $\mathtt {<}  \texttt {run\_type}\mathtt {>}$ values. This allows to compute the various contributions to the NLO cross section of a specific channel.

**Figure:**
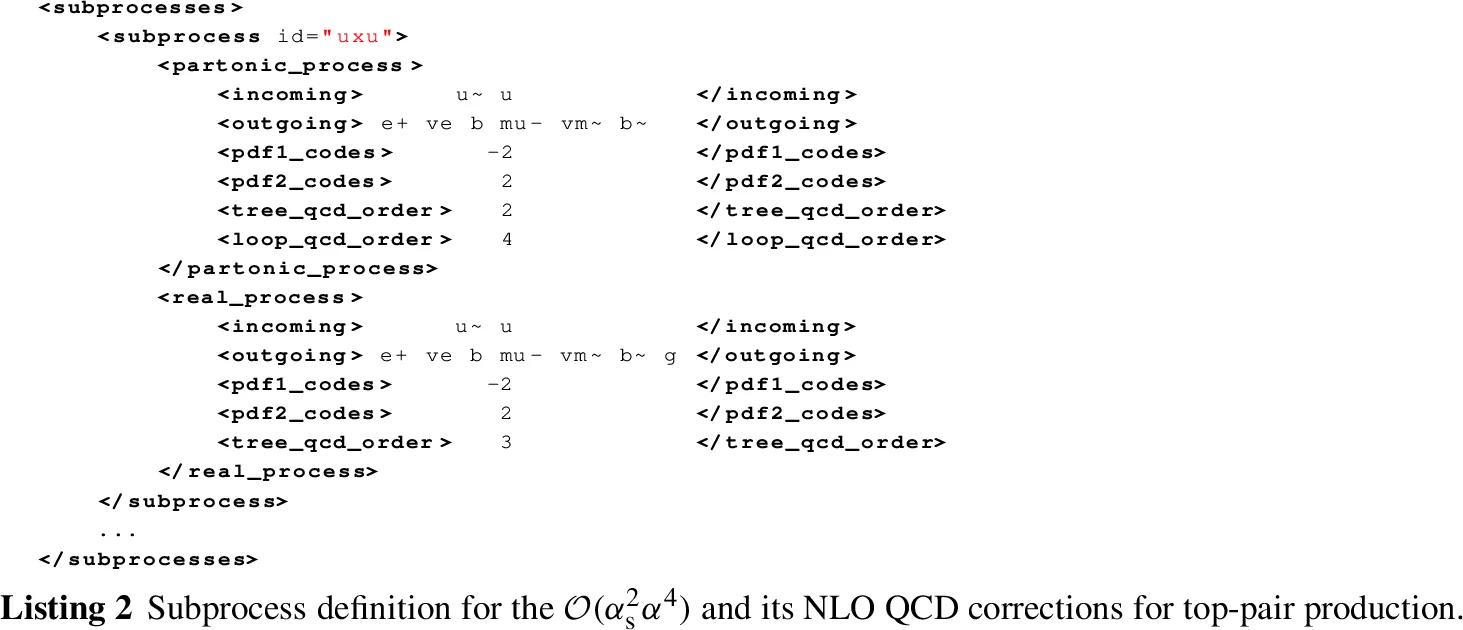

The parameters of both the $\mathtt {<}  \texttt {partonic\_process}\mathtt {>}$ and the $\mathtt {<}  \texttt {real\_process}\mathtt {>}$ are summarised in Table 3.

**Table 3:** Parameters of the $\mathtt {<}  \texttt {partonic\_process}\mathtt {>}$ or $\mathtt {<}  \texttt {real\_process}\mathtt {>}$ subsections of proc_card.xml and their default values

Parameter	Possible values	Default value
$\mathtt {<} \texttt {incoming}\mathtt {>}$	Pair of particle names	None
$\mathtt {<} \texttt {outgoing}\mathtt {>}$	List of particle names	None
$\mathtt {<} \texttt {pdf1\_codes}\mathtt {>}$	List of integers	None
$\mathtt {<} \texttt {pdf2\_codes}\mathtt {>}$	List of integers	None
$\mathtt {<} \texttt {tree\_qcd\_order}\mathtt {>}$	Integer	0
$\mathtt {<} \texttt {tree\_qcd\_order\_2}\mathtt {>}$	Integer	0
$\mathtt {<} \texttt {loop\_qcd\_order}\mathtt {>}$	Integer	0
$\mathtt {<} \texttt {interference}\mathtt {>}$	Integer	0
$\mathtt {<} \texttt {tree\_e0\_order}\mathtt {>}$	Integer	Guessed
$\mathtt {<} \texttt {loop\_e0\_order}\mathtt {>}$	Integer	Guessed

For both the particle content of the initial and final states is defined in the tags $\mathtt {<}  \texttt {incoming}\mathtt {>}$ and $\mathtt {<}  \texttt {outgoing}\mathtt {>}$, respectively, using the name convention for the particles reported in the second column of Table 4. The tags $\mathtt {<}  \texttt {pdf1\_codes}\mathtt {>}$ and $\mathtt {<}  \texttt {pdf2\_codes}\mathtt {>}$ indicate the PDF codes (using the numbering convention in the fourth column of Table 4) to be used for the first and second incoming beam, respectively.

**Table 4:** The different columns in the table report: particle names, identifiers in MoCaNLO and Recola, the particle codes according to the PDG MC particle numbering scheme, the jet types for the definition of recombinations and cuts, and the PDF codes according to LHAPDF. Antiparticles of non-self-conjugated particles have the negative PDG and PDF code and a “ ” added to the MoCaNLO and Recola identifier (except for particles with explicit charge signs in the identifier, which change for the antiparticle). Jet types are usually the same for the antiparticle, except for the charged leptons (4, 16, 18) and the $\text {W} ^-$ boson (10). The different jet type for neutrinos in parenthesis can be switched on with the input option $\mathtt {<}  \texttt {neutrino\_species\_tagging}\mathtt {>}$

Name	MoCaNLO	Recola	PDG code	Jet type	PDF code
$\text {d}$	d	d	1	1	1
$\text {u}$	u	u	2	1	2
$\text {s}$	s	s	3	1	3
$\text {c}$	c	c	4	1	4
$\text {b}$	b	b	5	2	5
$\text {t}$	t	t	6	7	6
$\text {e} ^-$	e-	e-	11	5	8
$\text {e} ^+$	e+	e+	-11	4	-8
$\nu _\text {e}$	ve	nu_e	12	6 (21)	–
$\bar{\nu }_\text {e}$			-12	6 (22)	–
$\mu ^-$	mu-	mu-	13	17	9
$\mu ^+$	mu+	mu+	-13	16	-9
$\nu _\mu$	vm	nu_mu	14	6 (23)	–
$\bar{\nu }_\mu$			-14	6 (24)	–
$\tau ^-$	ta-	tau-	15	19	10
$\tau ^+$	ta+	tau+	-15	18	-10
$\nu _\tau$	vt	nu_tau	16	6 (25)	–
$\bar{\nu }_\tau$			-16	6 (26)	–
γ	a	A	22	3	7
$\text {g}$	g	g	21	1	0
$\text {Z} _0$	z	Z0	23	8	–
$\text {W} ^+$	w+	W+	24	9	–
$\text {W} ^-$	w-	W-	-24	10	–
$\text {H}$	h	H	25	11	–

The orders of the amplitude in the QCD coupling $g_\textrm{s}$ are fixed in MoCaNLO with the help of additional tags. The orders in the EW coupling *e* follow for a given amplitude from the number of external particles. We use the usual quantities $\alpha _\text {s} =g_\text {s}^2/(4\pi )$ and $\alpha =e^2/(4\pi )$.

The value of the tag $\mathtt {<}  \texttt {tree\_qcd\_order}\mathtt {>}$ defines the power of the strong coupling $g_\text {s}$ at the amplitude level. For the virtual contribution, which originates from the interference of tree-level and one-loop amplitudes, the QCD order is fixed by specifying separately the powers of $g_\text {s}$ entering the tree-level and one-loop amplitudes using the $\mathtt {<}  \texttt {tree\_qcd\_order}\mathtt {>}$ and $\mathtt {<}  \texttt {loop\_qcd\_order}\mathtt {>}$ tags, respectively. An example of the usage of these tags is given in Listing 2. There, the $\mathcal {O}(\alpha _\text {s} ^2\alpha ^4)$ and its NLO QCD corrections to the cross section are specified for a partonic channel contributing to the example process $\text {p} \text {p} \rightarrow \text {e} ^+\nu _\text {e} \text {b} \mu ^-\bar{\nu }_\mu \bar{\text {b}}$.

For processes that receive contributions of different orders in the QCD coupling at LO, the additional tags $$\mathtt {<}  \texttt {interference}\mathtt {>}$$ and $$\mathtt {<}  \texttt {tree\_qcd\_order\_2}\mathtt {>}$$ are needed. Contributions of interferences between LO matrix elements with different coupling orders can be obtained by setting $$\mathtt {<}  \texttt {interference}\mathtt {>}$$$${}=1$$ together with $$\mathtt {<}  \texttt {tree\_qcd\_order}\mathtt {>}$$ and $$\mathtt {<}  \texttt {tree\_qcd\_order\_2}\mathtt {>}$$ to the power of the strong coupling of the two interfering matrix elements. Interferences between one-loop and tree-level matrix elements are obtained in an analogous way by specifying $$\mathtt {<}  \texttt {loop\_qcd\_order}\mathtt {>}$$ and $$\mathtt {<}  \texttt {tree\_qcd\_order\_2}\mathtt {>}$$, and interferences of loop-induced amplitudes by $$\mathtt {<}  \texttt {tree\_qcd\_order}\mathtt {>}$$ and $$\mathtt {<}  \texttt {tree\_qcd\_order\_2}\mathtt {>}$$. Note that when $$\mathtt {<}  \texttt {interference}\mathtt {>}$$ is not specified or set to 0, the tag $$\mathtt {<}  \texttt {tree\_qcd\_order\_2}\mathtt {>}$$ is ignored. This feature is needed for our example process $$\text {p} \text {p} \rightarrow \text {e} ^+\nu _\text {e} \text {b} \mu ^-\bar{\nu }_\mu \bar{\text {b}}$$, which receives at LO three different contributions from the $$\mathcal {O}(\alpha _\text {s} ^2\alpha ^4)$$, $$\mathcal {O}(\alpha _\text {s} \alpha ^5)$$, and $$\mathcal {O}(\alpha ^6)$$. To evaluate the $$\mathcal {O}(\alpha _\text {s} \alpha ^5)$$, for instance, one needs to interfere amplitudes with different powers of $$g_\text {s}$$, as exemplified in Listing 3.

**Figure:**
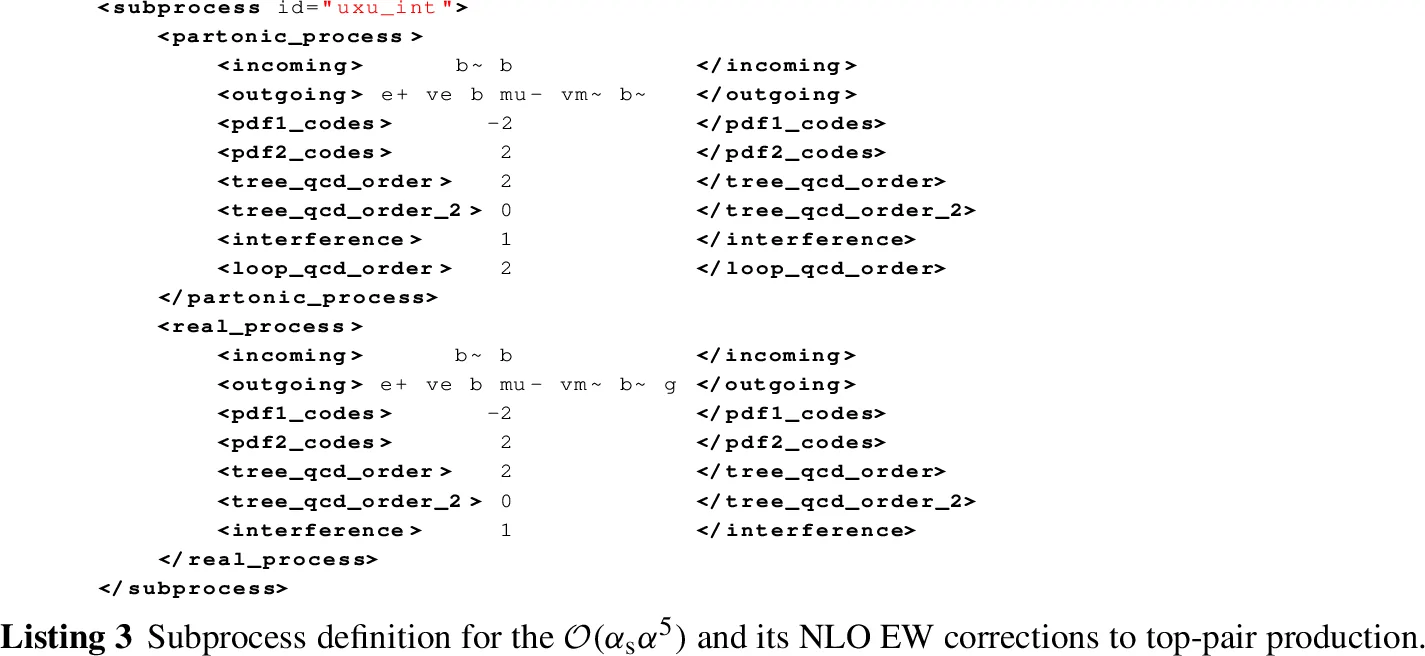

**Figure:**
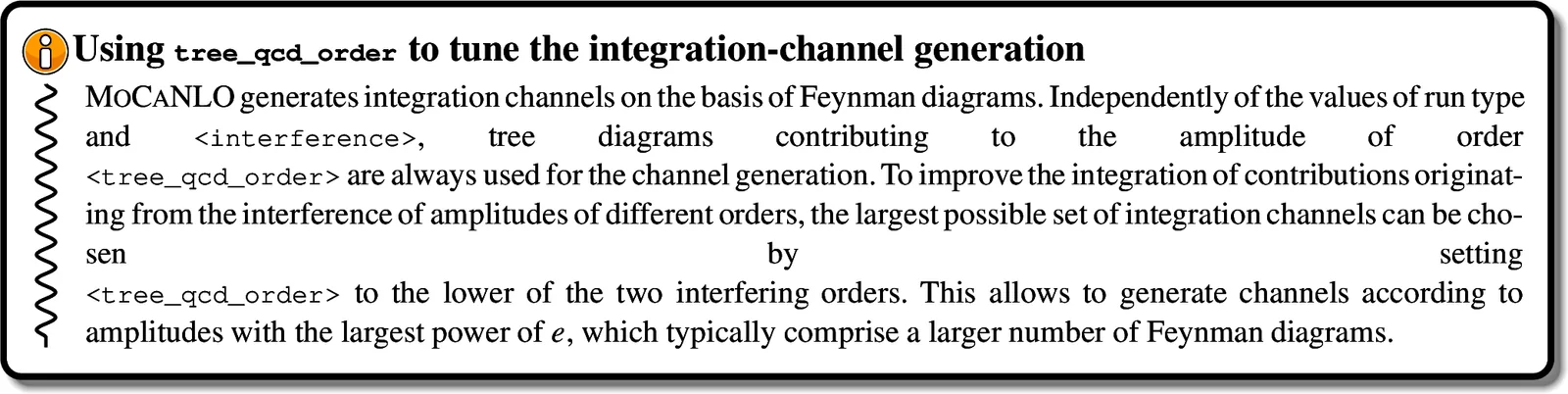

For processes with external on-shell photons, the corresponding couplings should always be renormalised at zero momentum transfer in order to avoid a dependence on the light quark masses [26]. This can be ensured by specifying the number of electromagnetic couplings α(0) at tree and one-loop level using the tags $\mathtt {<}  \texttt {tree\_e0\_order}\mathtt {>}$ and $\mathtt {<}  \texttt {loop\_e0\_order}\mathtt {>}$, respectively, while the remaining electromagnetic couplings are renormalised using the scheme chosen via $\mathtt {<}  \texttt {scheme\_alpha}\mathtt {>}$ in the param_card.xml. This is illustrated in Listing 4 for the partonic process $\bar{\text {u}}\text {u} \rightarrow \text {e} ^+\text {e} ^-\gamma$ and its EW correction. Note that in this example only the coupling of one photon is fixed to $\sqrt{\alpha (0)}$.

**Figure:**
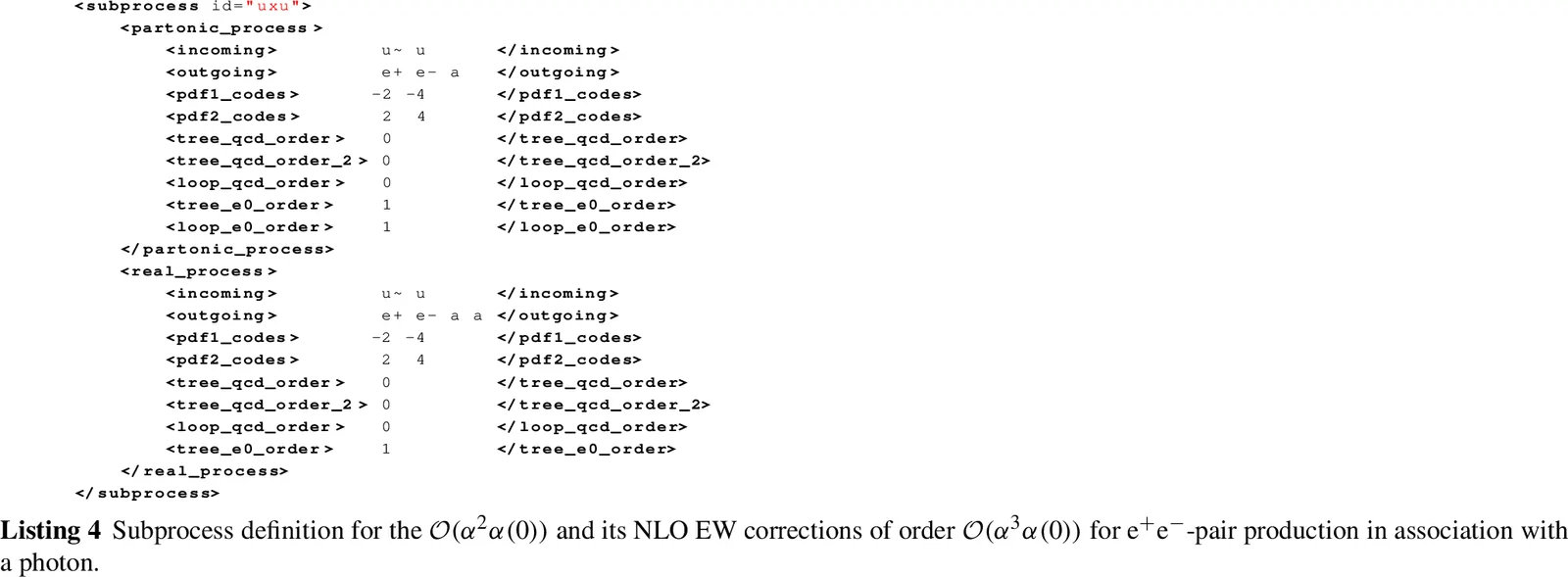

Alternatively, also the coupling of the real-emission photon could be set to $$\sqrt{\alpha (0)}$$ by setting $$\mathtt {<}  \texttt {tree\_e0\_order}\mathtt {>}$$=2 in $$\mathtt {<}  \texttt {real\_process}\mathtt {>}$$. However, in this case one has to additionally include $$\mathtt {<}  \texttt {loop\_e0\_order}\mathtt {>}$$$${}=2$$ in $$\mathtt {<}  \texttt {partonic\_process}\mathtt {>}$$ in order not to spoil the cancellation of IR singularities.

If $\mathtt {<}  \texttt {tree\_e0\_order}\mathtt {>}$ and/or $\mathtt {<}  \texttt {loop\_e0\_order}\mathtt {>}$ are not specified in proc_card.xml, MoCaNLO uses the following defaults. For processes without external photons, both are set to zero. For processes with external photons, both are set to the number of photons in the final state of $\mathtt {<}  \texttt {partonic\_process}\mathtt {>}$. Since the treatment of a possible bremsstrahlung photon is not unique for a real process, MoCaNLO stops and offers a guess. If this guess corresponds to the desired choice, it can be accepted by adding the tag

**Figure:**
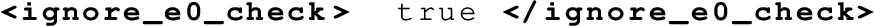

in the $\mathtt {<}  \texttt {run\_parameters}\mathtt {>}$ section of param_card.xml, which prevents the code from stopping. Note that a proper subtraction of infrared singularities requires the same value for $\mathtt {<}  \texttt {tree\_e0\_order}\mathtt {>}$ for the $\mathtt {<}  \texttt {real}\mathtt {>}$ and $\mathtt {<}  \texttt {idip}\mathtt {>}$ runs.

**Figure:**
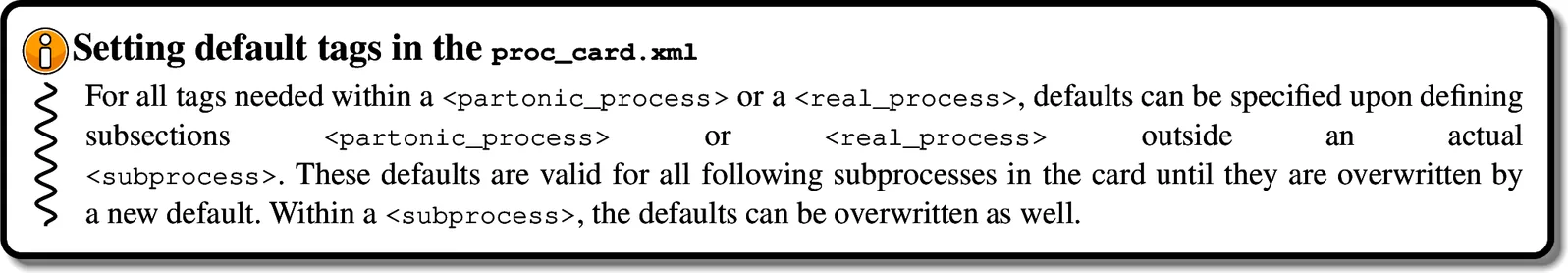

A standard computation at NLO accuracy (in either the $\alpha _\text {s}$ or the α coupling) requires to run contributions of Born (*B*), virtual (*V*), integrated-counterterm (*I*), and subtracted-real (R-D) type. More in detail, the differential cross section in a generic observable $\mathcal {O}$ at NLO reads,

1 $$\begin{aligned} \frac{{\text {d}} \sigma ^{\text {NLO}}}{\text {d} \mathcal {O}}= &   \int _{\text {cuts}} \text {d} \Phi _n\,\delta _n({\mathcal {O}})\left( B + V + I\right) \nonumber \\  &   +\int _{\text {cuts}} \text {d} \Phi _{n+1}\bigl [\delta _{n+1}({\mathcal {O}})\,R-\delta _{n}(\mathcal {O})\,D\bigr ], \end{aligned}$$

where the labels *n* and n+1 indicate that cuts and the evaluation of the observable should be applied (after possible recombination and isolation) to Born-like and real-correction-like phase-space kinematics, respectively. These four contributions are identified in MoCaNLO by the born, virt, idip, and real run types. The local subtraction counterterms (*D*) and the corresponding integrated counterparts (*I*) enable the subtraction of IR singularities of QED and QCD type. In the term dubbed *I*, all integrated counterparts of the local counterterms *D* are collected, including also initial-state collinear and PDF-renormalisation terms. In MoCaNLO the counterterms are implemented in the Catani–Seymour dipole formalism [13–18] and limited to IR-singular configurations caused by massless external particles (with the exception of NLO EW corrections to processes with intermediate $\text {W}$ bosons in the pole approximation, as detailed in Sect. 5.2.2).

Infrared-finite, loop-induced contributions must be handled with the lpid run type. Other run types can be used for the calculation of contributions in the pole approximation as described in Sect. 5.2.3, specifically for the virtual non-factorisable corrections (vnfc), for the *I*-operator part of integrated counterterms (idpi), and for the *P*- and *K*-operator parts of the integrated dipole contributions (idpk).

It is important to note that, depending on the run_type of a given run, only some information of the $\mathtt {<}  \texttt {subprocess}\mathtt {>}$ block is used.

- **born**: this type is intended for processes that receive tree-level contributions at LO. Only the $\mathtt {<}  \texttt {partonic\_process}\mathtt {>}$ part of the subprocess definition is used, with the order of the amplitudes computed using tree_qcd_order and if relevant tree_e0_order; when interference is set to 1, tree_qcd_order_2 is also used.
- **virt**: this is intended for one-loop virtual corrections to processes that receive contributions at tree level. Only the $\mathtt {<}  \texttt {partonic\_process}\mathtt {>}$ part of the subprocess definition is used, with the order of the amplitudes computed using loop_qcd_order and tree_qcd_order, or, when interference is set to 1, tree_qcd_order_2; if relevant also tree_e0_order and loop_e0_order enter.
- **vnfc**: only the $\mathtt {<}  \texttt {partonic\_process}\mathtt {>}$ part of the subprocess definition is used, with the order of the amplitude computed as for the born run_type. This type is dedicated to the computation of virtual non-factorisable contributions in the pole approximation (see Sect. 5.2.3). Its usage is only allowed if the section  is specified in the run_card.xml (see Sect. 5.2). The type of non-factorisable corrections to be computed is steered by tree_qcd_order and loop_qcd_order: if loop_qcd_order > tree_qcd_order non-factorisable QCD corrections are evaluated, and if loop_qcd_order = tree_qcd_order the EW ones are selected. If interference = 1, tree_qcd_order_2, and loop_qcd_order are instead compared to choose between the two types of corrections.
- **lpid**: this run type is intended for loop-induced processes. It works as a born one, where the orders of the loop diagrams are specified with $\mathtt {<}  \texttt {tree\_qcd\_order}\mathtt {>}$ (and not by loop_qcd_order), and, if interference is set to 1, $\mathtt {<}  \texttt {tree\_qcd\_order\_2}\mathtt {>}$, thus allowing to calculate loop-induced interference contributions. Analogously, the orders of $\sqrt{\alpha (0)}$ are specified via $\mathtt {<}$tree_e0_order$\mathtt {>}$.
- **real**: a real contribution comprises the real squared amplitude and all Catani–Seymour subtraction counterterms needed to have a finite result. The information to define the real squared amplitude is taken from the $\mathtt {<}  \texttt {real\_process}\mathtt {>}$ block. The same applies for the construction of its subtraction counterterms. Their complete list is obtained considering all underlying Born processes that can be reached from the real process by discarding a candidate emissus particle and replacing the candidate emitter particle by the splitting one. The underlying Born processes whose particle content is not compatible with the single-particle-cut requirements (see Sect. 7.3) specified in the cut_card.xml are discarded already at this level, to prevent the computation of contributions that would eventually be cut away. Note that the PDF codes in the $\mathtt {<}  \texttt {real\_process}\mathtt {>}$ part are also the relevant ones for the computation of the subtraction terms. Note that, when the real is treated in the pole approximation (see Sect. 5.2.2), the code also uses the information of the $\mathtt {<}  \texttt {incoming}\mathtt {>}$ and $\mathtt {<}  \texttt {outgoing}\mathtt {>}$ from the $\mathtt {<}  \texttt {partonic\_process}\mathtt {>}$ .
- **idip**: for this run type both the $\mathtt {<}  \texttt {partonic\_process}\mathtt {>}$ and the $\mathtt {<}  \texttt {real\_process}\mathtt {>}$ parts are relevant. This contribution is computed using the PDF codes in the $\mathtt {<}  \texttt {real\_process}\mathtt {>}$ part. It results from the sum of dipoles constructed as for the real case that also match the particle content (up to ordering of the final state) and the amplitude order of the underlying Born process specified in the $\mathtt {<}  \texttt {partonic\_process}\mathtt {>}$ block. Only these dipoles are taken into account in the result. This means that the complete set of integrated counterterms is obtained by running multiple idip runs whose $\mathtt {<}  \texttt {subprocess}\mathtt {>}$ definitions have identical $\mathtt {<}  \texttt {real\_process}\mathtt {>}$ parts, but different $\mathtt {<}  \texttt {partonic\_process}\mathtt {>}$ as underlying Born processes. Also note that, since integrated dipoles are defined on a Born-like kinematics, the integration channels are constructed from the $\mathtt {<}  \texttt {partonic\_process}\mathtt {>}$ amplitude.
- **idpi**/**idpk**: these two additional types can be run exactly as the idip run_type described above. Even if they can be used both with and without the specification to the section  in the run_card.xml (see Sect. 5.2), their purpose is to guarantee the IR safety of the full result when a pole approximation is applied only to the virtual contributions (as described in Sect. 5.2.3). Note that the contribution idpk cannot be calculated in the pole approximation: If it is linked to a section  of the proc_card.xml with pole_approximation=true, the code stops.

In Listing 5 the usage of the run types real and idip is further illustrated. The example shows some subprocesses needed to compute QCD corrections of $$\mathcal {O}(\alpha _\text {s} \alpha ^6)$$ to the pure EW LO contribution to $$\text {W} \text {Z} $$ VBS. All three subprocesses with IDs real_b1, real_b2, and real_b3 define the same real squared amplitude, but differ in their $$\mathtt {<}  \texttt {partonic\_process}\mathtt {>}$$ block. The real contribution to the channel $$\text {u} \text {u} \rightarrow \text {e} ^+\nu _\text {e} \mu ^+\mu ^-\text {u} \text {d} \text {g} $$ is obtained by running only one of the three subprocesses with run type real. To properly account for all integrated dipoles, instead all three subprocesses must be run separately with run type idip. Then, the subprocess with  computes integrated QCD dipoles with underlying Born of order $$\mathtt {<}  \texttt {tree\_qcd\_order}\mathtt {>}$$$${}=0$$, while the subprocesses with  and  compute QED dipoles with two different underlying Born processes (resulting from a photon emitted from the incoming quark and entering the hard process) of order $$\mathtt {<}  \texttt {tree\_qcd\_order}\mathtt {>}$$$${}=1$$.

**Figure:**
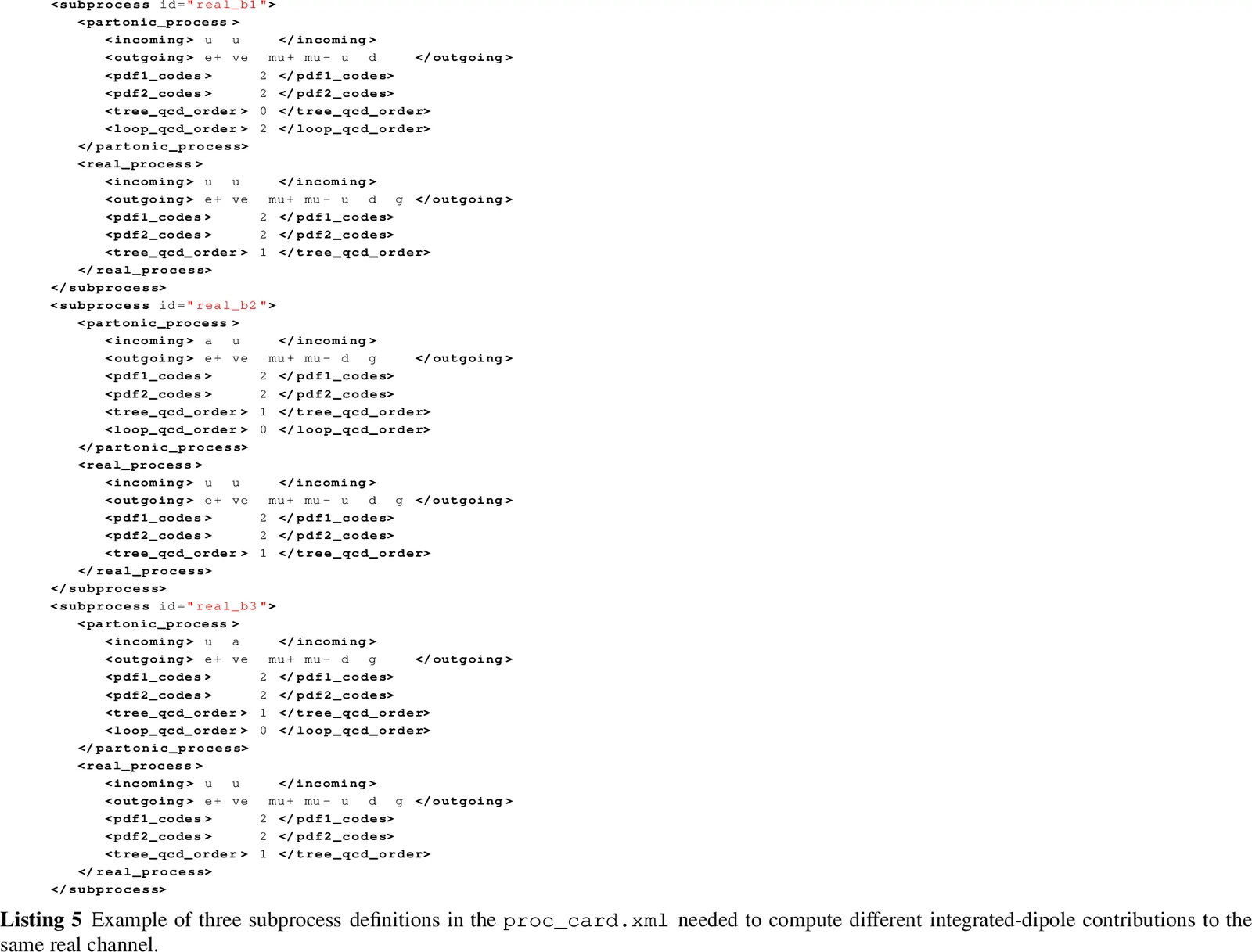

#### Merging of partonic channels

Having separate runs for different partonic channels and NLO contributions allows MoCaNLO to tune the integration channels and improve the integration efficiency. This is crucial for processes having a non-trivial resonance structure. Nonetheless, for high-multiplicity final states, the number of contributions to be computed as separate runs grows very fast and can potentially become a bottleneck. To ameliorate this problem, MoCaNLO supports two ways of computing different partonic channels together, which may be used simultaneously.

**Figure:**
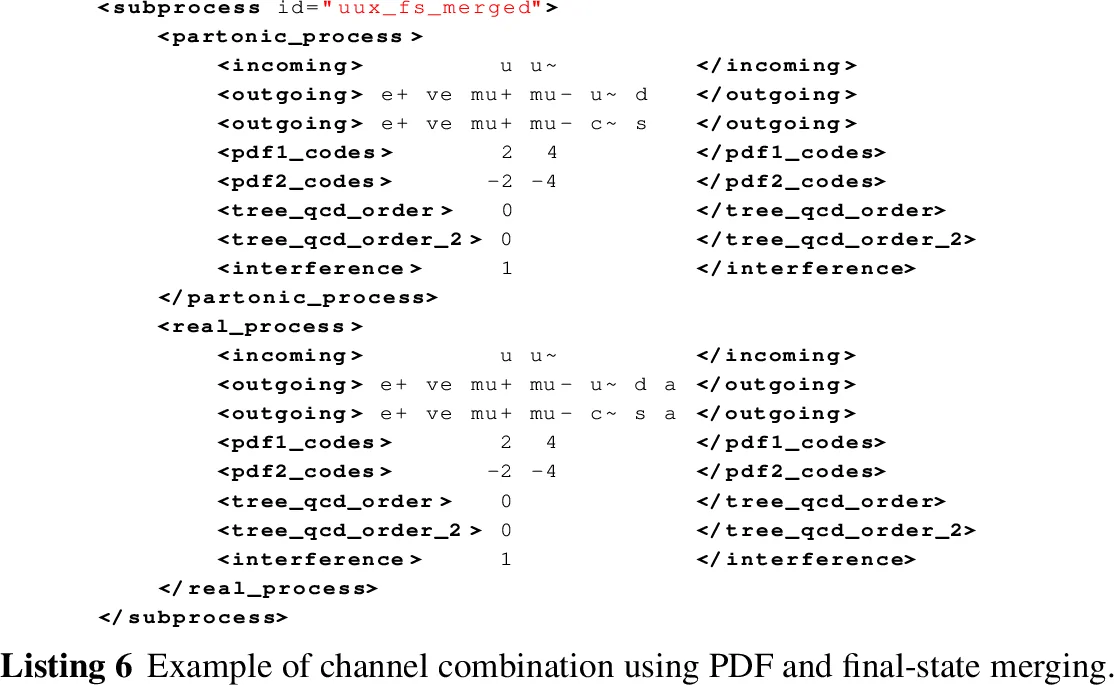

**PDF merging** The tags $\mathtt {<}  \texttt {pdf1\_codes}\mathtt {>}$ and $\mathtt {<}  \texttt {pdf2\_codes}\mathtt {>}$, which define the PDF codes for the first and second beam, can be initialised to strings of integers, like $\{i_1,\dots ,i_n\}$ and $\{j_1,\dots ,j_m\}$, respectively. Their length should be the same for both tags (i.e. n=m), since values occupying the same positions in the string enter the calculation in pairs, namely only the combinations $\{(i_1,\,j_1),\dots ,(i_n,\,j_n)\}$ are considered and no cross terms. The resulting cross section will be the sum of as many contributions as the length of the strings. Each contribution is obtained by weighting the same squared amplitude with different PDF factors. The underlying assumption is that amplitudes for the considered process are identical when exchanging the flavours $i_1\leftrightarrow i_k$ and $j_1\leftrightarrow j_k$ $\forall k\in \{2,\dots ,n\}$ both in the initial and in the final states.

The lines containing $\mathtt {<}  \texttt {pdf1\_codes}\mathtt {>}$ and $\mathtt {<}  \texttt {pdf2\_codes}\mathtt {>}$ in Listing  illustrate the usage of PDF merging for partonic processes contributing to $\text {W} ^+\text {Z}$ VBS. In the example the partonic channels $\text {u} \bar{\text {u}}\rightarrow \text {e} ^+\nu _e\mu ^+\mu ^-\bar{\text {u}}\text {d} (\gamma )$ and $\text {c} \bar{\text {c}}\rightarrow \text {e} ^+\nu _e\mu ^+\mu ^-\bar{\text {c}}\text {s} (\gamma )$ are computed in one run. This combination is allowed, since the two processes only differ by PDF factors, but have identical matrix elements if the quarks are massless and the CKM matrix is set to the identity matrix.

**Final-state merging** If the definition of a $\mathtt {<}$partonic_process$\mathtt {>}$/$\mathtt {<}  \texttt {real\_process}\mathtt {>}$ includes multiple $\mathtt {<}  \texttt {outgoing}\mathtt {>}$ tags, partonic channels sharing the same initial state and only differing in their final states are merged into one MoCaNLO run. When the process is initialised, the code generates all integration channels for the different processes and eliminates identical ones. For the born and virtual types, the list of processes in the $\mathtt {<}  \texttt {partonic\_process}\mathtt {>}$block is combined, while for the real type only the list in $\mathtt {<}  \texttt {real\_process}\mathtt {>}$ is relevant. For the idip type, both $\mathtt {<}  \texttt {partonic\_process}\mathtt {>}$ and $\mathtt {<}  \texttt {real\_process}\mathtt {>}$ are important. For each real process at a given position in the list of $\mathtt {<}  \texttt {outgoing}\mathtt {>}$ tags of $\mathtt {<}  \texttt {real\_process}\mathtt {>}$, the code computes only the dipoles which match the underlying Born at the same position in the $\mathtt {<}  \texttt {outgoing}\mathtt {>}$ tags of $\mathtt {<}  \texttt {partonic\_process}\mathtt {>}$. Both when running the real and the idip, identical dipoles and dipoles only differing in some charge/colour factors are computed once and properly accounted for in the combined result.

**Figure:**
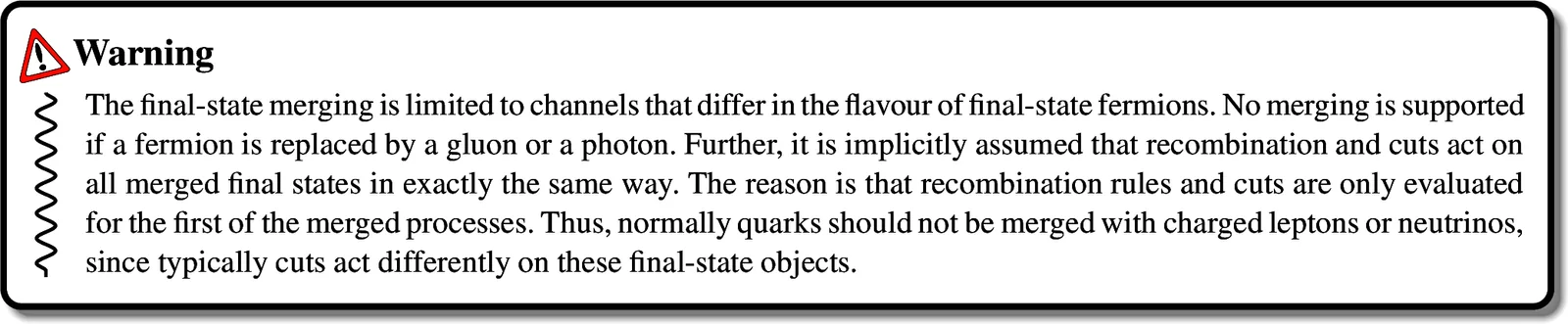

**Figure:**
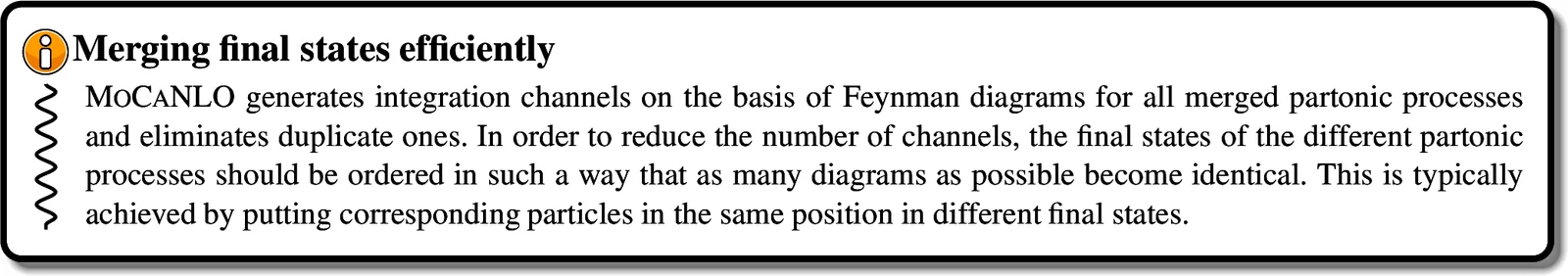

In Listing  the syntax for integrating together the channels $\text {u} \bar{\text {u}}\rightarrow \text {e} ^+\nu _e\mu ^+\mu ^-\bar{\text {u}}\text {d} (\gamma )$ and $\text {u} \bar{\text {u}}\rightarrow \text {e} ^+\nu _e\mu ^+\mu ^-\bar{\text {c}}\text {s} (\gamma )$ is explicitly shown. Since in this example the final-state merging is used in combination with the PDF merging, the channels $\text {c} \bar{\text {c}}\rightarrow \text {e} ^+\nu _e\mu ^+\mu ^-\bar{\text {c}}\text {s} (\gamma )$ and $\text {c} \bar{\text {c}}\rightarrow \text {e} ^+\nu _e\mu ^+\mu ^-\bar{\text {u}}\text {d} (\gamma )$ are also implicitly accounted for.

### Treatment of resonances

Many applications focus on contributions involving a specific set of intermediate resonances for a given process. The user can require a desired set of resonances by linking in the run_card.xml a section , where XX is the ID that denotes a particular resonance specification. This optional section must be provided in the proc_card.xml as shown in Listing 7.

**Figure:**
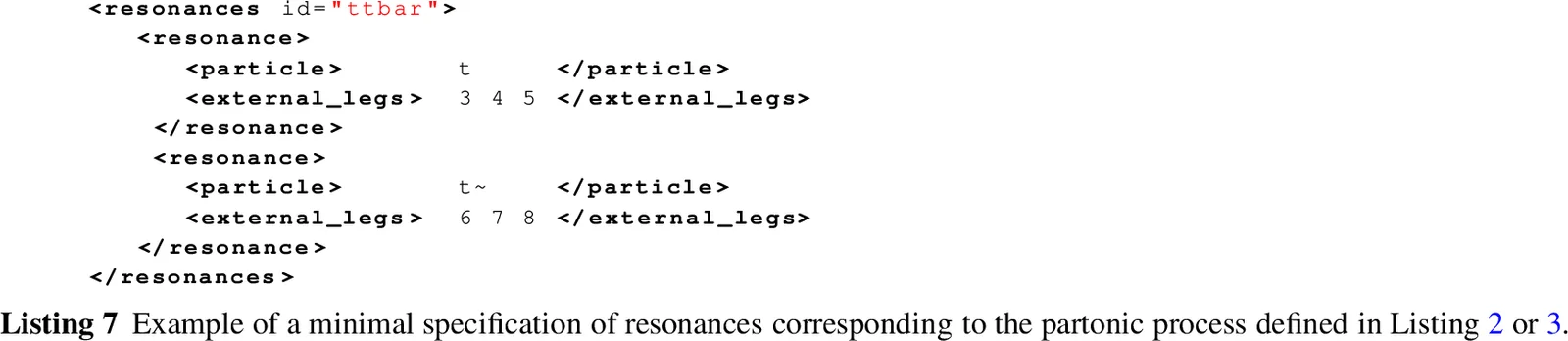

In each resonances section, multiple resonances can be required. The information on each individual resonance is enclosed in the XML subsection $\mathtt {<}  \texttt {resonance}\mathtt {>}$, whose available parameters are summarised in Table 5.

**Table 5:** Parameters of the $\mathtt {<}  \texttt {resonance}\mathtt {>}$ section of proc_card.xml and their default values

Parameter	Possible values	Default value
$\mathtt {<} \texttt {particle}\mathtt {>}$		–
$\mathtt {<} \texttt {external\_legs}\mathtt {>}$	list of integers (see text)	–
$\mathtt {<} \texttt {phase\_space\_only}\mathtt {>}$	true, false	true
$\mathtt {<} \texttt {pole\_approximation}\mathtt {>}$	true, false	false
$\mathtt {<} \texttt {polarisation\_state}\mathtt {>}$	-1, +1, 0, T, U	U

The resonance type is specified in $\mathtt {<}  \texttt {particle}\mathtt {>}$ according to its particle name as detailed in column 2 of Table 4. The tag $\mathtt {<}  \texttt {external\_legs}\mathtt {>}$ defines the decay products of the resonance in terms of their positions in the list of $\mathtt {<}  \texttt {outgoing}\mathtt {>}$ (with counting starting from 3) of the subprocess pointing to this . As long as the particle types of the decay products are consistent with the resonance, their position in the list of $\mathtt {<}  \texttt {outgoing}\mathtt {>}$ is not constrained, i.e. they are not forced to be adjacent. In the example in Listing 7, the top and antitop resonances define the requirement of the decay chain $\text {t} \rightarrow \text {e} ^+\nu _\text {e} \text {b}$ and $\bar{\text {t}}\rightarrow \mu ^-\bar{\nu }_\mu \bar{\text {b}}$, respectively, for the process $\text {p} \text {p} \rightarrow \text {e} ^+\nu _\text {e} \text {b} \mu ^-\bar{\nu }_\mu \bar{\text {b}}$.

By default, the presence of a resonances section tells MoCaNLO to construct only the integration channels that are compatible with the specified resonances, but it does not affect which contributions enter the matrix elements. This reduction of the amount of channels can speed up the convergence of the integration, because there are less channels to sample from. On the other hand, if there are significant contributions to the matrix elements in the considered fiducial region that are not sampled by the specified subset of channels, the convergence might be worse. For more information about the construction of integration channels we refer to Refs. [6–9].

Setting to false the additional tag $\mathtt {<}  \texttt {phasespace\_only}\mathtt {>}$ (default true) in a specific $\mathtt {<}  \texttt {resonance}\mathtt {>}$ subsection causes the decay chain to also affect the matrix-element computation, namely only diagrams including that resonance will be part of the calculation. This is the starting point to define squared amplitudes including one or more on-shell resonances. Nevertheless, such a definition only based on a resonant-diagram selection breaks gauge invariance. This is why $\mathtt {<}  \texttt {phasespace\_only}\mathtt {>}$ should only be set to false in combination with the $\mathtt {<}  \texttt {pole\_approximation}\mathtt {>}$ tag (default false), where the matrix element is treated with a generic pole approximation algorithm, discussed in further detail in the following subsection.

#### Pole approximation: general features

The pole approximation [29] is based on the pole scheme [26–28], which provides a gauge-invariant way to separate resonant and non-resonant contributions at the level of the matrix element. When in MoCaNLO the XML resonance section includes the $\mathtt {<}  \texttt {phasespace\_only}\mathtt {>}$ tag set to false in combination with $\mathtt {<}  \texttt {pole\_approximation}\mathtt {>}$ set to true, the pole approximation is applied. Thus, only diagrams involving the specified resonances are included in the matrix elements. In addition, an on-shell-projected kinematics is used throughout the calculation except in the evaluation of resonances’ propagators and when applying phase-space cuts, i.e. an event is still accepted or rejected according to its off-shell kinematics before on-shell projection. In Listing 8 two possible pole approximations are shown for the VBS process $\text {p} \text {p} \rightarrow \text {e} ^+\nu _\text {e} \mu ^-\bar{\nu }_\mu \text {j} \text {j}$, whose $\mathtt {<}  \texttt {incoming}\mathtt {>}$ and $\mathtt {<}  \texttt {outgoing}\mathtt {>}$ lists for a specific subprocess are given at the beginning of the same Listing.

**Figure:**
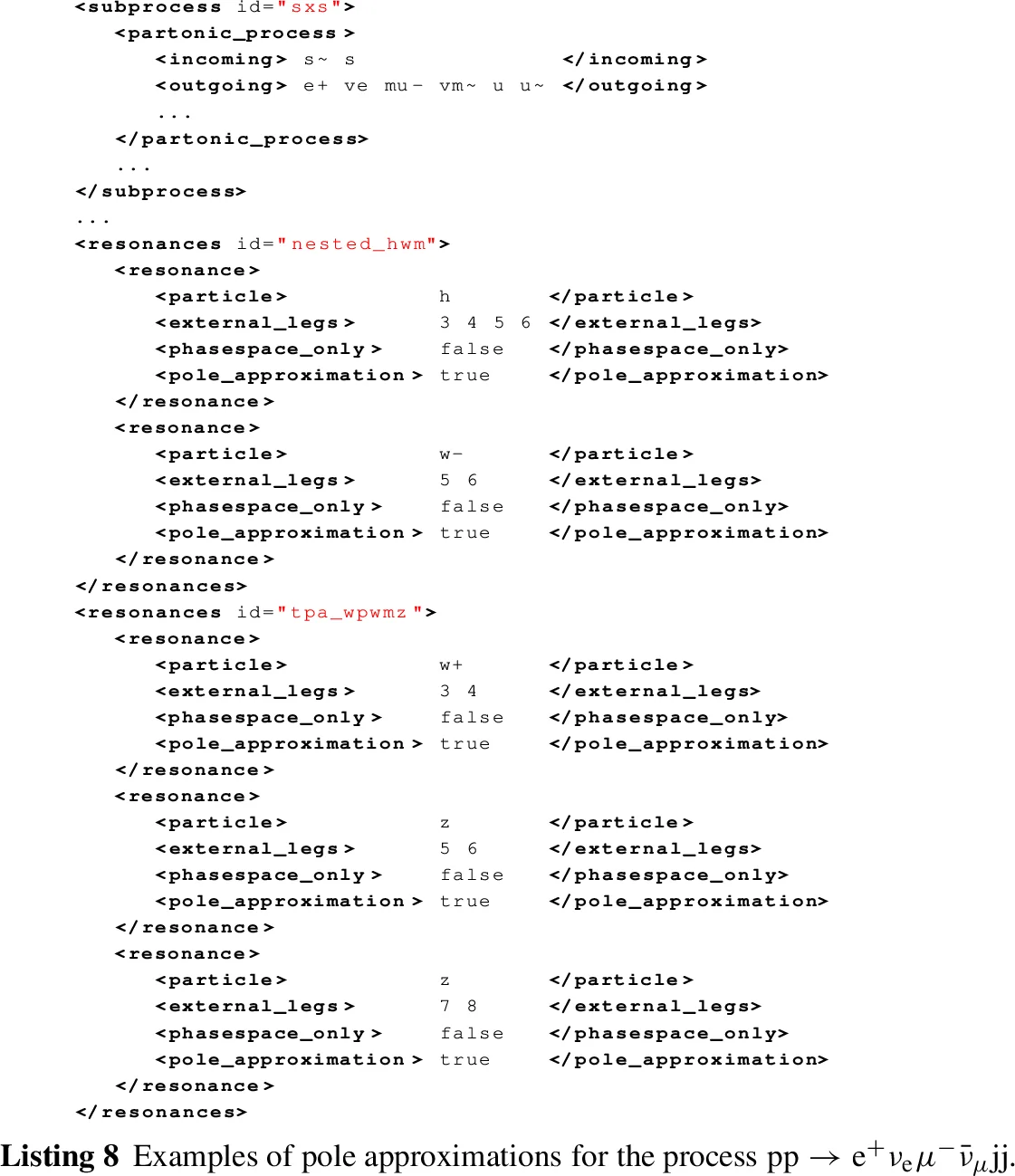

In the block , a pole approximation with a nested Higgs resonance is illustrated. There, two sets of external legs defining the Higgs and the W-boson resonances overlap. In order to deal with nested resonances within the pole approximation, MoCaNLO makes use of the general on-shell projection algorithm presented in Ref. [57]. The very same algorithm can also deal with single-pole approximations. A special treatment is only required for a few cases, which are separately discussed at the end of Sect. 5.2.2

**Table 6:** Parameters of the $\mathtt {<}  \texttt {on\_shell\_projection}\mathtt {>}$ section of proc_card.xml and their default values

Parameter	Possible values	Default value
$\mathtt {<} \texttt {preserve\_system}\mathtt {>}$	cms, cms_res	cms
$\mathtt {<} \texttt {poldef\_system}\mathtt {>}$	cms, cms_res, lab	value of $\mathtt {<} \texttt {preserve\_system}\mathtt {>}$
$\mathtt {<} \texttt {preserve\_directions}\mathtt {>}$	list of integers (see text)	–
$\mathtt {<} \texttt {keep\_resonance\_widths}\mathtt {>}$	0, 1	0
$\mathtt {<} \texttt {search\_invariants}\mathtt {>}$	0, 1	0
$\mathtt {<} \texttt {subsystem}\mathtt {>}$	full, res, nres, nres_tot,	full
	res+nres, res+nres_tot	
$\mathtt {<} \texttt {priority}\mathtt {>}$	list of resonance identifiers	h t* w* z
	(as in second column of Table 4)	
$\mathtt {<} \texttt {fudge\_factor}\mathtt {>}$	0, 1	0
$\mathtt {<} \texttt {fudge\_factor\_born}\mathtt {>}$	0, 1	0
$\mathtt {<} \texttt {fudge\_width\_range}\mathtt {>}$	real number	3.0

The behaviour of the on-shell projection can be tuned via the  block in the proc_card.xml, which is linked in the subprocess definition in the run_card.xml. The elements of this block are summarised in Table 6. The on-shell projection implemented in MoCaNLO always preserves at least one invariant, which is by default the partonic CM energy. Using the tag $\mathtt {<}  \texttt {preserve\_system}\mathtt {>}$, the system whose total momentum is preserved can be changed from the default one, i.e. the partonic CM system (cms), to the one defined by the sum of the momenta of the on-shell-projected resonances (cms_res). Note that, when running a real contribution as part of an NLO calculation, the radiated particle is always included in the preserved system if $\mathtt {<}  \texttt {preserve\_system}\mathtt {>}$ is set to cms. If instead cms_res is chosen, the radiation is included only for real corrections to the decays (osp_type${}>0$), while excluded for real corrections to the production (osp_type${}=0$). See discussion in Sect. 5.2.2.

Furthermore, the user may require to preserve additional invariants via the input tags of the sub-block $\mathtt {<}  \texttt {preserve\_invariants}\mathtt {>}$. When the tag $\mathtt {<}$search_invariants$\mathtt {>}$ (default 0) is set to 1, the code loops over all integration channels and selects the ones maximising the number of resonances that are not projected on shell. By default, Higgs bosons are given the highest priority, followed by top quarks, W bosons, and Z bosons. This behaviour can be modified using the input $\mathtt {<}  \texttt {priority}\mathtt {>}$ and providing a list of resonances as MoCaNLO identifiers (as defined in second column of Table 4). Different priorities can be required for particles and antiparticles of the same resonance using the appropriate identifiers, e.g. t/  and w+/w- for top quarks and W bosons, respectively. To give the same importance to particles and antiparticles, a star key can be used next to the resonance identifier, e.g. t* or w*. Resonances in the first entries of the list receive higher priority and integration channels with the largest number of propagators associated to them are selected. If no channel with at least one resonance of the $\mathtt {<}  \texttt {priority}\mathtt {>}$ list can be found, the code stops, since no invariant fulfilling the input criteria can be constructed. Conversely, if a subset of channels is found, the code further filters them according to its default priority criteria. Additionally, more importance is given to those channels that involve resonances decaying into a smaller amount of particles. Once an integration channel is finally selected, invariants are collected from all resonant propagators that are not set on shell. Invariants corresponding to the partonic CM energy are excluded. Moreover, since the algorithm tries to search for invariants that can be preserved both at LO and NLO, invariants including momenta from all final-state photons and/or gluons are also discarded. The final selection of preserved invariants is printed to the standard output of the run.

An additional filter on the invariants to be preserved can be activated via the flag $$\mathtt {<}  \texttt {subsystem}\mathtt {>}$$. By default (full), all invariants are kept. By choosing $$\mathtt {<}  \texttt {subsystem}\mathtt {>}$$$${}={}$$res, only invariants in the sub-system defined by the decay products of the on-shell-projected resonances are kept, while the value nres restricts the momenta of the invariants to the complementary system, i.e. all final-state momenta that are not part of on-shell-projected resonances. Invariants from both sets are chosen with the value res+nres. Moreover, using nres_tot and res+nres_tot allows to preserve the invariant of the complementary system as a whole. This latter option can be used for instance to address the invariant mass of the tag jets in the context of VBS (see Ref. [57]). Note that the set of additionally preserved invariants can only be a subset of the system defined by $$\mathtt {<}  \texttt {preserve\_system}\mathtt {>}$$, meaning that when the latter is set to cms_res, $$\mathtt {<}  \texttt {subsystem}\mathtt {>}$$ can only be set to res, otherwise the code stops.

An example of an  block is shown in Listing 9. There, the conservation of the CM energy is required explicitly using the $\mathtt {<}  \texttt {preserve\_system}\mathtt {>}$ tag. In the block $\mathtt {<}  \texttt {preserve\_invariants}\mathtt {>}$, additional invariants to be preserved are searched in the full final state, giving higher priority to the ones that can be associated to intermediate W bosons (regardless of the charge) and, with lower priority, to intermediate Z bosons.

**Figure:**
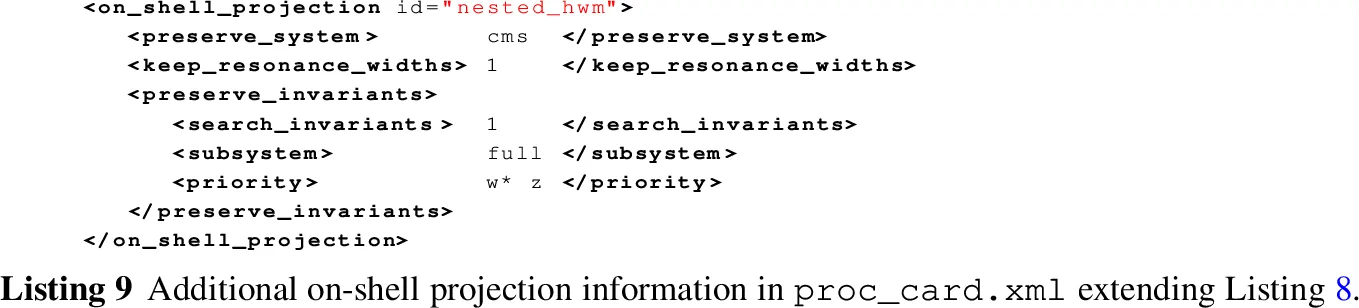

In some applications one may want to preserve the directions of a subset of final-state particles. By default, the on-shell projection described in Ref. [57] preserves all directions of the decay products of a resonance in the rest frame of the latter, but not in the laboratory frame. This behaviour can be modified via the optional $\mathtt {<}  \texttt {preserve\_directions}\mathtt {>}$ tag in the $\mathtt {<}  \texttt {on\_shell\_projection}\mathtt {>}$ section. The latter must contain a list of integers referring to the position of the final-state particles whose off-shell directions in the laboratory frame one wants to preserve. This feature, which allows to reproduce the on-shell projection introduced in Ref. [29], is only available for resonances decaying into two particles, where no more than one decay-product direction per resonance can be preserved, i.e. each integer in the $\mathtt {<}  \texttt {preserve\_directions}\mathtt {>}$ list must belong to a different resonance. Moreover, this option cannot be used for a real run type.

As part of the pole-approximation algorithm, the widths of the on-shell-projected resonances must be set to zero everywhere in the calculation except in the propagators of the specified resonances. Nevertheless, when a pole approximation with *n* resonances is performed, a process may have a larger number of resonances than the specified ones with the same types of particles. This is the case for our example subprocess , which also admits triply-resonant contributions, as evidenced by the second pole approximation  in Listing 8. When this happens, setting the decay widths of the resonances to zero causes the phase-space integration to run into singularities, unless they are excluded by cuts. Even if no general solution to the problem exists, MoCaNLO offers two ways to handle divergent resonant propagators in the context of the pole approximation.

A simple workaround is to keep the resonance widths non-zero [65]. This can be enforced by switching on the tag $\mathtt {<}  \texttt {keep\_resonance\_widths}\mathtt {>}$ in the $\mathtt {<}  \texttt {on\_shell\_projection}\mathtt {>}$ section (see Listing 9). If not set, the code works as usual and sets to zero the decay widths in the pole-approximated squared amplitudes except in the propagators of the required resonances.

**Figure:**
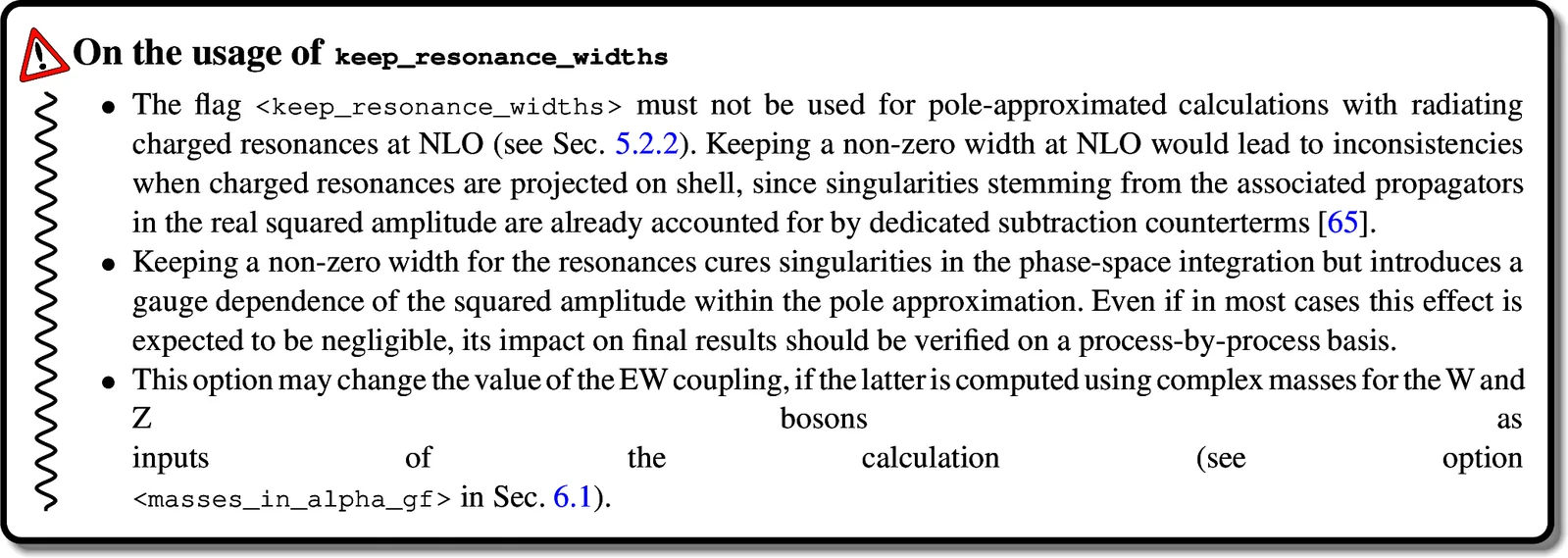

If a process in the pole approximation is protected at LO against singularities from extra resonant propagators by the definition of the fiducial phase space, so that problems can only appear at NLO, MoCaNLO offers an alternative regularisation. Its usage is controlled via the block $\mathtt {<}  \texttt {fudge\_factor\_regularisation}\mathtt {>}$. Within this block, if $\mathtt {<}  \texttt {fudge\_factor}\mathtt {>}$ is set to 1, squared amplitudes for the real and idip contributions receive correction factors that regularise all resonant propagators that appear in a given partonic process. The propagators are found by the code by looping over all integration channels and collecting all resonant propagators that are not projected on shell. For each propagator *i* a factor $\mathcal {F}_i$ is constructed,

2 $$\begin{aligned} \mathcal {F}_i(s_i)= {\left\{ \begin{array}{ll} 1 &  \quad \text {if}\quad |\sqrt{s_i}-M_i|>c_{\mathcal {F}}\,\Gamma _i\\ \frac{(s_i-M^2_i)^2}{(s_i-M^2_i)^2+\Gamma ^2_iM^2_i} &  \quad \text {if}\quad |\sqrt{s_i}-M_i|<c_{\mathcal {F}}\,\Gamma _i \end{array}\right. }\,, \end{aligned}$$

where $s_i$ refers to the invariant mass of the unregularised resonance *i* having a pole mass $M_i$ and a width $\Gamma _i$. The full fudge factor results from the products of the $\mathcal {F}_i$ for all propagators found by the algorithm. In the previous formula, the parameter $c_{\mathcal {F}}$ restricts the range of application of the fudge factor. Its value can be controlled by the input $\mathtt {<}  \texttt {fudge\_width\_range}\mathtt {>}$ (default value 3.0). Finally, even if the fudge-factor regularisation is mostly useful to deal with resonance singularities appearing at NLO, the usage of the fudge factor to regularise LO processes can also be enforced by setting $\mathtt {<}  \texttt {fudge\_factor\_born}\mathtt {>}$ to 1 (when also $\mathtt {<}  \texttt {fudge\_factor}\mathtt {>}$ has been set to 1).

**Figure:**
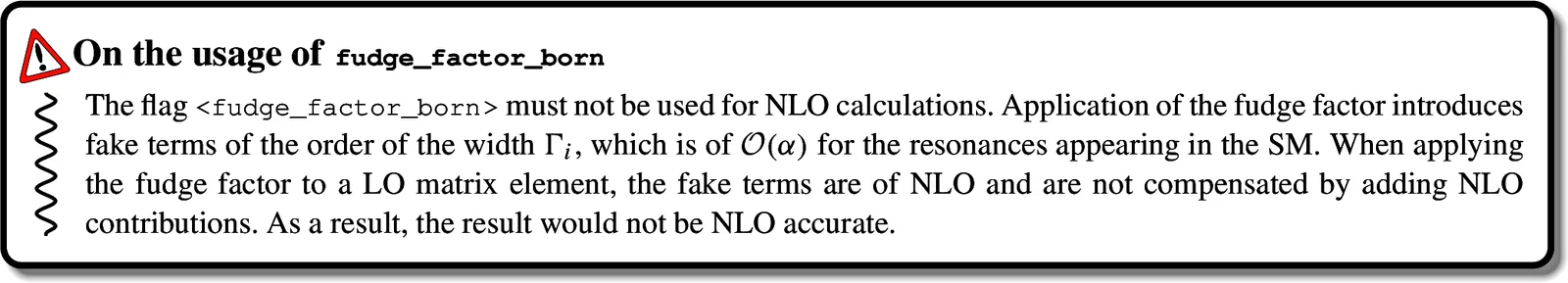

A block $\mathtt {<}  \texttt {fudge\_factor\_regularisation}\mathtt {>}$ is illustrated in Listing 10. This example must be read together with Listing 11, which defines the pole approximation for the process $\text {p} \text {p} \rightarrow \text {e} ^+\nu _\text {e} \mu ^-\bar{\nu }_\mu \text {j} \text {j}$. In the context of an NLO calculation (see Sect. 5.2.2), the section $\mathtt {<}  \texttt {on\_shell\_projection}\mathtt {>}$ of Listing 10 regularises the resonant propagators other than the ones projected on shell.

**Figure:**
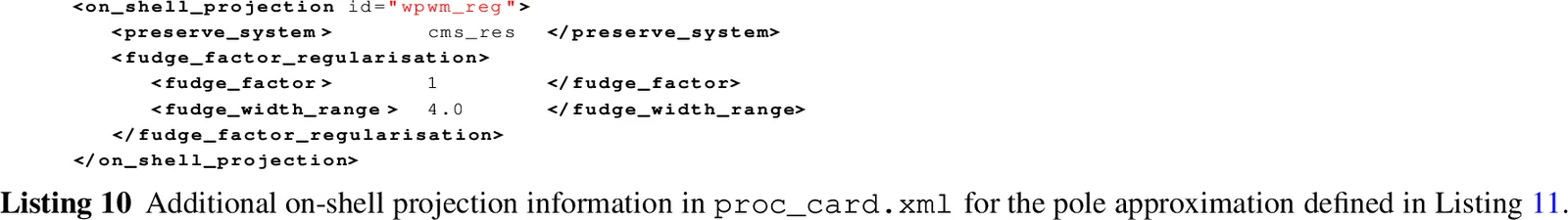

As a final observation, it is worth noting that, while the use of pole approximations is supported along with the final-state merging of different partonic processes, all merged partonic processes must have the same resonance structure. If this is not the case, the code will still compute the correct results but with a very low convergence rate.

#### Pole approximation at NLO

While MoCaNLO can deal with resonances in the pole approximation in full generality at LO, only a restricted set of processes is currently supported in pole approximation at NLO accuracy. Limitations essentially come from the treatment of the soft and collinear singularities in the real-radiation contributions, which becomes more cumbersome compared to the full off-shell description.

In particular, a fully-fledged NLO (QCD or EW) calculation in the pole approximation is only possible under the following conditions:

- The process involves *one or two weak bosons* as intermediate states, treated in the single- or double-pole approximation, respectively. Neither processes with three or more resonances, nor those with a nested resonance structure can be handled at NLO. Top quarks cannot be treated either in this regard.
- The resonances undergo *two-body leptonic decays* at LO, and the real-radiation contributions lead to resonances that decay at most into three particles, i.e. *up to three-body decays in real corrections* at NLO. Hadronic decays of resonances are not supported.
- Additional limitations arise for *charged resonances*. Since an intermediate charged resonance can also radiate, dedicated counterterms are required for a complete subtraction of singularities, when such a resonance is projected on shell (for more details see Ref. [65]). MoCaNLO only supports processes at NLO with leptonically-decaying $\text {W}$ bosons. Colour-charged resonances like top quarks cannot be handled at NLO within the pole approximation.
- As a final remark, a full treatment of *non-factorisable corrections* [31, 32] is not supported. Even if both factorisable and non-factorisable corrections can be computed for the virtual contribution (as discussed in Sect. 5.2.3), only factorisable ones are available for the real contribution. In any case, the impact of these corrections is known to be small if both the real and virtual contributions are treated in the pole approximation, since in their sum non-factorisable corrections cancel in observables that are inclusive with respect to the decay products of the resonances [30, 74–77].

An NLO calculation in the pole approximation for a given subprocess can be performed by running all relevant contributions (born, virt, idip, and real) with the linking tag  pointing to the same XML resonances block in the proc_card.xml, which selects a particular pole approximation, as explained in Sect. 5.2.1. Additionally, for the real part one needs to compute separate contributions where the radiation is emitted from the production and the decay of the *N* on-shell-projected resonances. This means that one is required to submit N+1 separate real runs for a given subprocess to obtain a complete NLO pole-approximated result. Note that the radiated particle for the real in the pole approximation is identified by comparing the $\mathtt {<}  \texttt {partonic\_process}\mathtt {>}$ and the $\mathtt {<}  \texttt {real\_process}\mathtt {>}$: This is sufficient for the cases supported by MoCaNLO , where a real configuration only admits one underlying Born. The information on the origin of the radiation is instead provided directly in the run_card.xml as part of the subprocess definition, as illustrated in Listing 11. This is done using the tag $\mathtt {<}  \texttt {osp\_type}\mathtt {>}$ (default value 0), which assumes the value 0 for the production contribution, or any number from 1 to N for the decay contributions of the *N* resonances. These numbers refer to the ordering in which resonances are listed in the shared  block.

**Figure:**
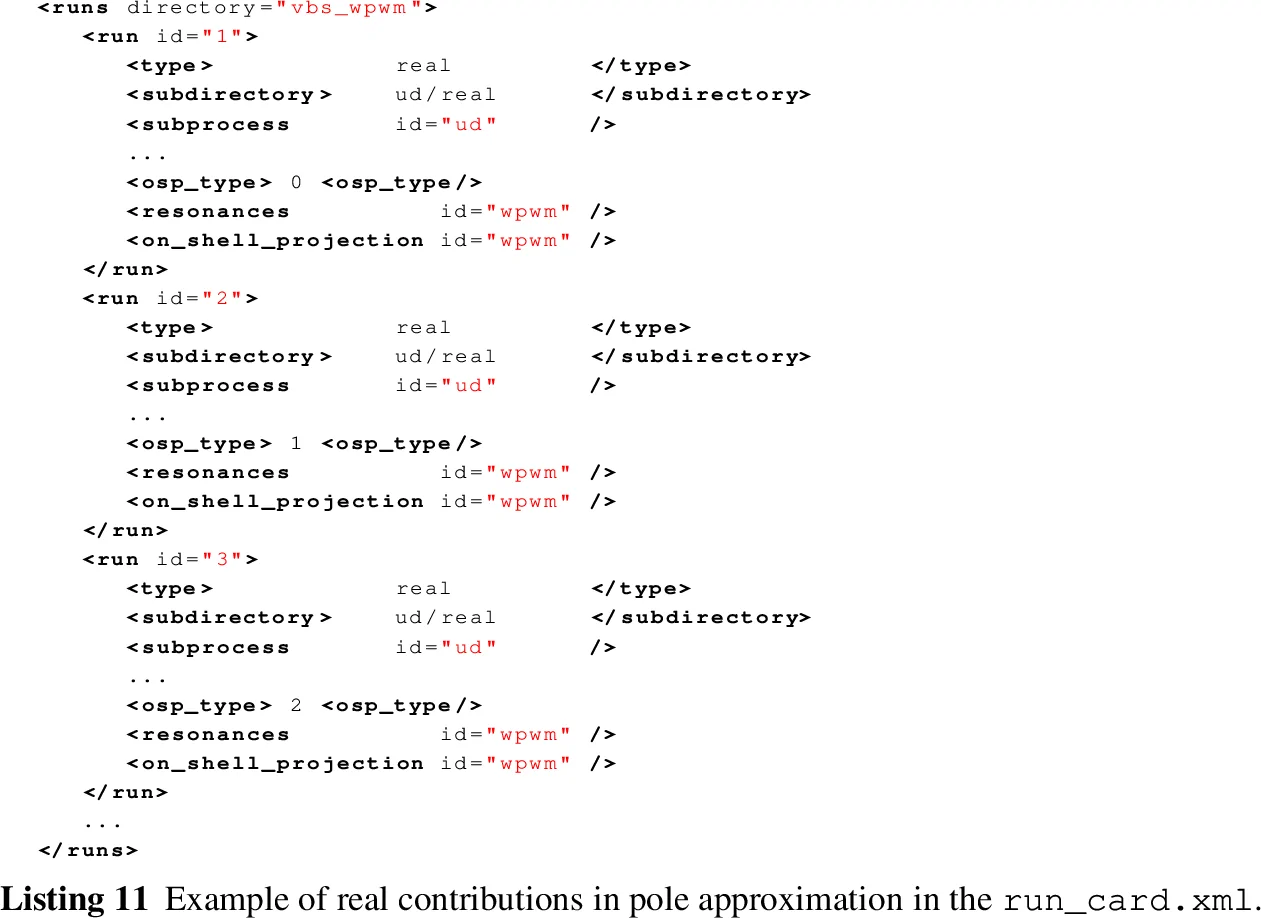

A possible proc_card.xml for the real contributions in Listing 11 is shown in Listing 12, where a double-pole approximation for the process $\text {p} \text {p} \rightarrow \text {e} ^+\nu _\text {e} \mu ^-\bar{\nu }_\mu \text {j} \text {j}$ is considered. Note that the position of the radiation in the $\mathtt {<}  \texttt {outgoing}\mathtt {>}$ list of $\mathtt {<}  \texttt {real\_process}\mathtt {>}$ (a photon in our example) does not need to be contiguous to the decay products of the on-shell-projected resonances (the leptons in our example). For illustrative purposes, the example in Listing 12 requires to preserve the total momentum of the two $\text {W}$ bosons that are projected on shell via the $\mathtt {<}  \texttt {preserve\_system}\mathtt {>}$ tag. This choice is usually more natural than the default one for specific applications, for example when studying vector-boson polarisations (see Sect. 5.2.4).

**Figure:**
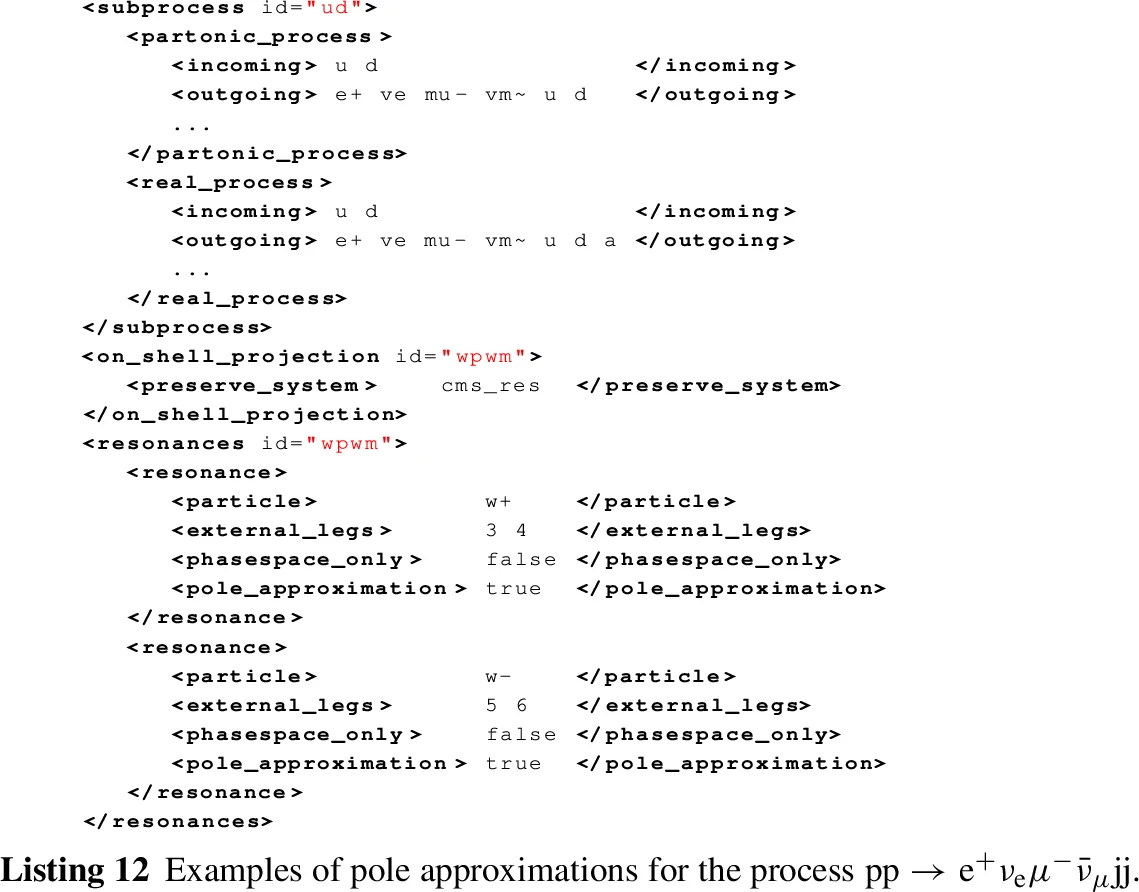

**Figure:**
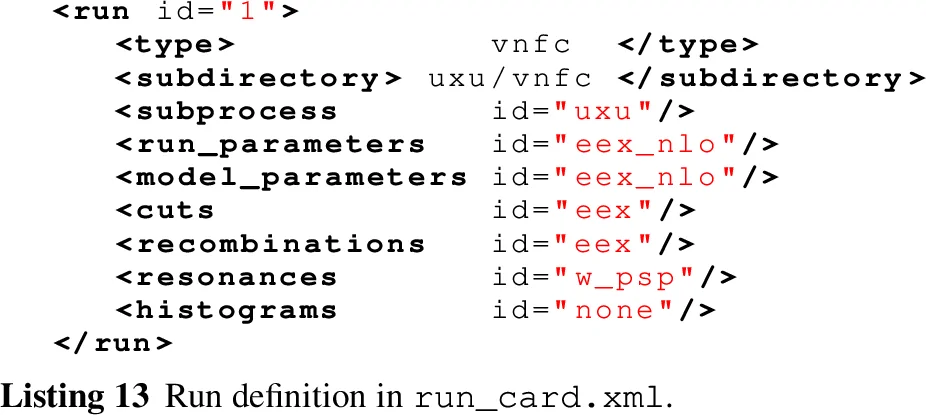

**Further remarks on the single-pole approximation** The general pole-approximation algorithm implemented in MoCaNLO [57] covers also calculations in the single-pole approximation, up to special treatments needed for a few cases.

- Two-to-two process: at variance with the general case, the on-shell projection rescales initial-state momenta and (when computing NLO corrections) the final-state momentum of the radiation, if this is not part of the decay products of the resonance of interest. The rescaling factor is equal to the ratio of the pole mass over the invariant mass of the resonance. Since in this case the on-shell projection has to modify the incoming momenta, it cannot even preserve the partonic CM energy. Therefore, the input provided via the tag $\mathtt {<}  \texttt {preserve\_system}\mathtt {>}$ is ignored.
- Single resonance with at least one additional massive external particle: the pole approximation proceeds as in the general case, but when evaluating a real contribution to the production, i.e. osp_type${}={}$0, the momentum of a bremsstrahlung particle (a photon or a gluon) is never included in the preserved system (in contrast to the default behaviour, if cms was chosen), i.e. the momentum of the radiation is never modified. For real corrections to the decays, no change in the algorithm is needed instead.

Note that the pole approximation for a single intermediate charged resonance produced in association with massless particles is ill defined close to threshold, unless the resonance’s invariant mass is constrained by kinematic cuts preventing it from becoming arbitrarily close to the pole mass. Consequently, processes with a single $\text {W}$ boson or a top quark in association with massless particles are not supported by MoCaNLO , while for instance cases with a single $\text {Z}$ boson decaying into charged-fermion pairs are supported and treated as in the general case.

#### Virtual non-factorisable corrections

Within the restrictions discussed in Sect. 5.2.2, MoCaNLO allows to compute all contributions (i.e. born, virt, idip, and real) in the pole approximation, including only factorisable corrections for the virtual and real terms. This provides a consistent NLO result in this approximation and is also the only way to define a polarised cross section, as described in Sect. 5.2.4. However, the pole approximation can also be used to speed up the computation of virtual corrections, whose evaluation is particularly expensive. A solution, which was adopted for instance in Refs. [29, 31], is to compute all contributions entering an NLO calculation exactly, with the exception of the virtual one, where a pole approximation was used. This requires in MoCaNLO to run for each subprocess a virt and a vfnc run type both specifying a  section in the run_card.xml. If both factorisable and non-factorisable virtual corrections are accounted for, their IR singularities must be cancelled applying the on-shell projection to the terms containing the *I* operator in the integrated dipole contribution in the same way. The *P*- and *K*-operator terms, on the other hand, must be evaluated with the off-shell kinematics like the real corrections. This introduces a mismatch that is of the order of the intrinsic error of the pole approximation. A proper evaluation of the *I* operator, in line with our discussion, can be achieved in MoCaNLO by replacing each idip contribution for a specific subprocess by an idpi and an idpk run pointing to the same subprocess. The idpi runs should specify a  section with pole_approximation=true, while the idpk ones must be run with off-shell kinematics. Examples of how to perform these kind of computations are provided in the folder validated_processes, in particular the processes in the double-pole approximation in the ttbar/pp_tt folder. It is worth noting that while the implementation of the non-factorisable corrections closely follows Refs. [30–32] and is therefore very general (arbitrary number of non-nested resonances), it has only been validated for double-pole approximations involving two identical resonant particles [34, 35, 38, 49].

**Figure:**
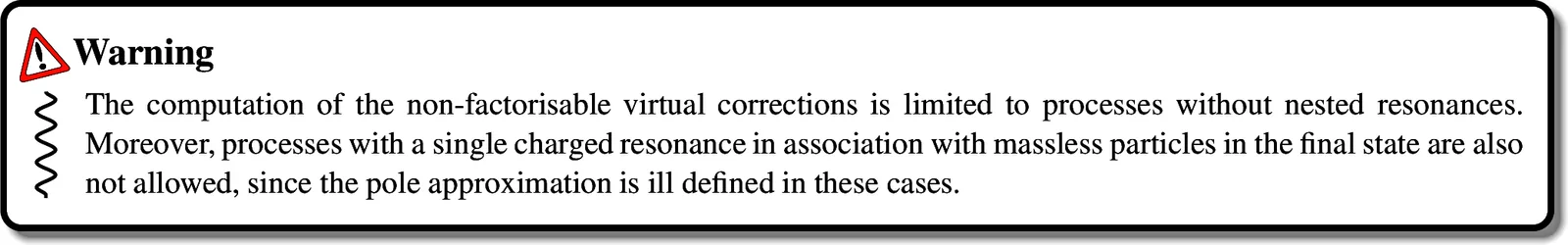

#### Selection of polarisations

MoCaNLO can compute cross sections for internal polarised vector bosons at NLO accuracy. The definition of polarised cross sections is based on the pole approximation, as described for instance in Ref. [59]. As a consequence, polarised results are only available for processes that can be calculated in this approximation (see discussion at the beginning of Sect. 5.2.2).

To run simulations for polarised vector bosons, one must add to each resonance block, where the pole approximation has been enabled, the required polarisation state via the tag $\mathtt {<}  \texttt {polarization\_state}\mathtt {>}$. Allowed values are reported in Table 7. In the example in Listing 14, two on-shell $\text {W}$ bosons having transverse and longitudinal polarisations are required.

**Table 7:** Available polarisation states

$\mathtt {<} \texttt {polarization\_state}\mathtt {>}$	**Description**
-1 / +1	Left- and right-handed
0	Longitudinal
T	Transverse
U	Unpolarised (default)

**Figure:**
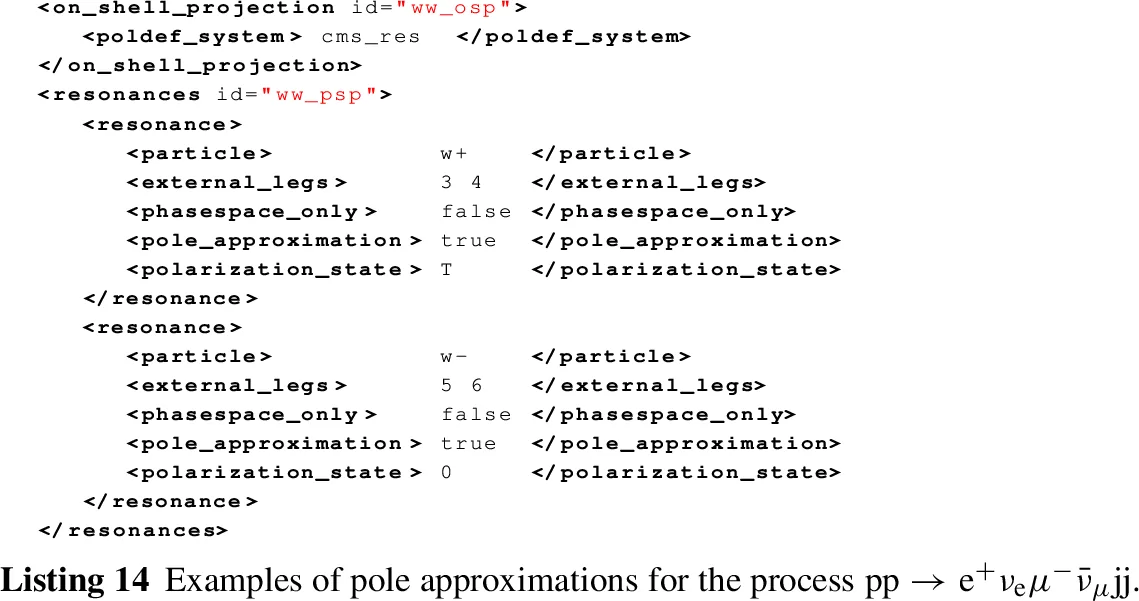

The definition of polarisation states is Lorentz-frame dependent, and consequently the result for the polarised cross sections too. By default, MoCaNLO defines polarisations in the same frame as requested in $\mathtt {<}  \texttt {preserve\_system}\mathtt {>}$ (see Sect. 5.2.1). Nonetheless, a different reference frame for the polarised states can be set in the XML section  using the $\mathtt {<}$poldef_system$\mathtt {>}$ tag. In Listing 14, the pole approximation preserves the partonic CM energy of the process (default when $\mathtt {<}  \texttt {preserve\_system}\mathtt {>}$ is not specified), but computes polarisations in the CM frame of the two vector bosons. The possible input values for $\mathtt {<}  \texttt {poldef\_system}\mathtt {>}$ (see Table 6) are those of $\mathtt {<}  \texttt {preserve\_system}\mathtt {>}$ and the value lab, which allows to define polarisations in the laboratory frame. Note that this is the default frame used for the definition of polarisations in case of a single-pole approximation.

## Specifying the simulation parameters: the param_card.xml

The parameter card param_card.xml contains two types of sections. These are labelled , where parameters of the particle model are given, and , which specifies parameters for the MC run. The contents of these two section types are discussed in the following.

A single param_card.xml can contain several subsections of the same type, each with a unique id attribute that is used for linking in the run_card.xml (see Sect. 4). This can be used to include different parameter sets, e.g. for LO and NLO calculations. The exemplary Listing 15 contains the NLO value of the top-quark width $\Gamma _\text {t}$ for the NLO calculation of the process $\text {p} \text {p} \rightarrow \text {e} ^+\nu _\text {e} \text {b} \mu ^-\bar{\nu }_\mu \bar{\text {b}}$ and is thus denoted with the attribute . An additional section  containing the LO value of $\Gamma _\text {t}$ could be present in param_card.xml. Similarly, different sections  and  could point to the usage of NLO and LO PDF sets, respectively.

### Model parameters

All parameters available in the $\mathtt {<}  \texttt {model\_parameters}\mathtt {>}$ section of param_card.xml are summarised in Tables 8 and 9 together with their default values, and Listing 15 shows an example of their use in param_card.xml.

**Table 8:** Elements of the $\mathtt {<}  \texttt {model\_parameters}\mathtt {>}$ section of param_card.xml and their defaults

Parameter	XML element	Default value
$m_\text {t}$	$\mathtt {<} \texttt {top\_mass}\mathtt {>}$	172.0
$\Gamma _\text {t}$	$\mathtt {<} \texttt {top\_width}\mathtt {>}$	1.4
$m_\text {b}$	$\mathtt {<} \texttt {bottom\_mass}\mathtt {>}$	0.0
$\Gamma _\text {b}$	$\mathtt {<} \texttt {bottom\_width}\mathtt {>}$	0.0
$m_\tau$	$\mathtt {<} \texttt {tau\_mass}\mathtt {>}$	0.0
$m_\mu$	$\mathtt {<} \texttt {muon\_mass}\mathtt {>}$	0.1056584
$m_\text {e}$	$\mathtt {<} \texttt {electron\_mass}\mathtt {>}$	$0.51099895\times 10^{-3}$
$M_{\text {Z}}^{\textrm{OS}}$	$\mathtt {<} \texttt {ons\_z0\_mass}\mathtt {>}$	91.1880
$\Gamma _\text {Z} ^{\textrm{OS}}$	$\mathtt {<} \texttt {ons\_z0\_width}\mathtt {>}$	2.4955
$M_\text {W} ^\textrm{OS}$	$\mathtt {<} \texttt {ons\_w\_mass}\mathtt {>}$	80.3692
$\Gamma _\text {W} ^\textrm{OS}$	$\mathtt {<} \texttt {ons\_w\_width}\mathtt {>}$	2.085
$M_\text {Z}$	$\mathtt {<} \texttt {z0\_mass}\mathtt {>}$	Calculated
$\Gamma _\text {Z}$	$\mathtt {<} \texttt {z0\_width}\mathtt {>}$	Calculated
$M_\text {W}$	$\mathtt {<} \texttt {w\_mass}\mathtt {>}$	Calculated
$\Gamma _\text {W}$	$\mathtt {<} \texttt {w\_width}\mathtt {>}$	Calculated
$M_\text {H}$	$\mathtt {<} \texttt {higgs\_mass}\mathtt {>}$	125.0
$\Gamma _\text {H}$	$\mathtt {<} \texttt {higgs\_width}\mathtt {>}$	$4.07\times 10^{-3}$
$G_\text {F}$	$\mathtt {<} \texttt {fermi\_constant}\mathtt {>}$	$1.166390\times 10^{-5}$
–	$\mathtt {<} \texttt {masses\_in\_alpha\_gf}\mathtt {>}$	0
–	$\mathtt {<} \texttt {scheme\_alpha}\mathtt {>}$	gf
$\alpha _{G_\mu }$	$\mathtt {<} \texttt {alphagf}\mathtt {>}$	$7.555310522369\times 10^{-3}$
α(0)	$\mathtt {<} \texttt {alpha0}\mathtt {>}$	$7.2973525\times 10^{-3}$
$\alpha (M_\text {Z} ^2)$	$\mathtt {<} \texttt {alphaz}\mathtt {>}$	$7.7983287\times 10^{-3}$
$\alpha _{\overline{{\text {MS}}}}(Q_0^2)$	$\mathtt {<} \texttt {alpha\_starting\_value}\mathtt {>}$	$7.8609176\times 10^{-3}$
$Q_0$	$\mathtt {<} \texttt {alpha\_starting\_scale}\mathtt {>}$	91.188
$\alpha _\text {s}$	$\mathtt {<} \texttt {alphas}\mathtt {>}$	none
–	$\mathtt {<} \texttt {alphas\_running}\mathtt {>}$	1 if hadrons in initial state, 2 otherwise
$\alpha _\text {s} (Q^2_0)$	$\mathtt {<} \texttt {alphas\_starting\_value}\mathtt {>}$	0.118
$Q_0$	$\mathtt {<} \texttt {alphas\_starting\_scale}\mathtt {>}$	91.188
–	$\mathtt {<} \texttt {n\_loops}\mathtt {>}$	2
$\mu _\text {R}$	$\mathtt {<} \texttt {renormalization\_scale}\mathtt {>}$	$\mu _\text {F}$ or $M_\text {Z}$
$\mu _\text {F}$	$\mathtt {<} \texttt {factorization\_scale}\mathtt {>}$	$\mu _\text {R}$ or $M_\text {Z}$
$\mu _\text {IR}$	$\mathtt {<} \texttt {infrared\_scale}\mathtt {>}$	100.0
–	$\mathtt {<} \texttt {neutrino\_species\_tagging}\mathtt {>}$	0
–	$\mathtt {<} \texttt {redefine\_jet\_types}\mathtt {>}$	–

**Table 9:** Additional technical parameters of the $\mathtt {<}  \texttt {model\_parameters}\mathtt {>}$ section of param_card.xml and their defaults

Parameter	XML element	Default value
$$m_\text {reg}$$	$$\mathtt {<} \texttt {massless\_mapping\_mass}\mathtt {>}$$	$$10^{-6} $$
*p*	$\mathtt {<} \texttt {massless\_mapping\_power}\mathtt {>}$	0.9
*q*	$\mathtt {<} \texttt {subtraction\_mapping\_power}\mathtt {>}$	0.8

**Figure:**
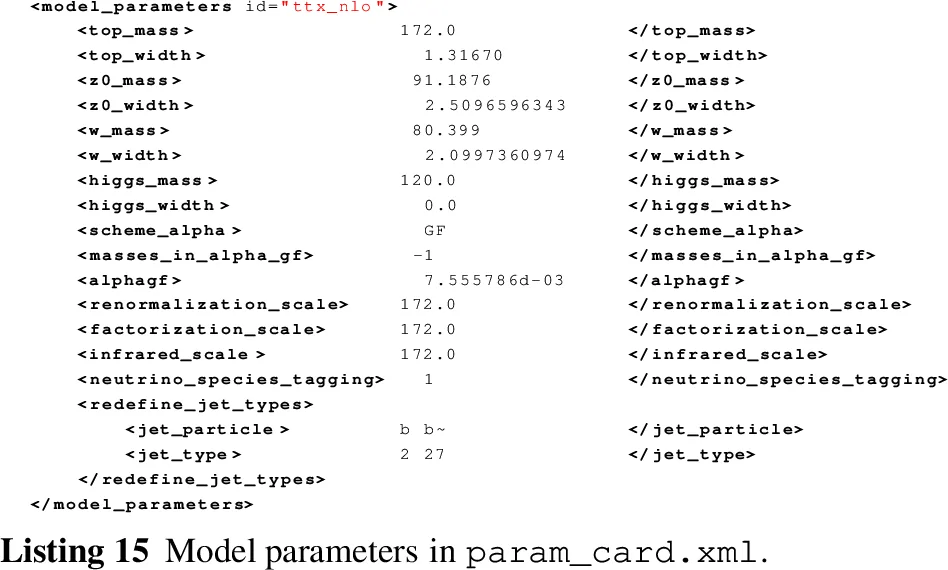

Masses and widths of particles in $\,\text {GeV}$ are set via $\mathtt {<}  \texttt {particle\_mass}\mathtt {>}$ and $\mathtt {<}  \texttt {particle\_width}\mathtt {>}$, respectively. The electron and muon masses are only used as regulators of initial-state singularities for incoming electrons/positrons or muons and are considered as massless otherwise. The four light quarks are considered as massless throughout. While the masses of the bottom quark and the τ lepton are zero by default, finite masses are allowed as long as these particles do not appear as radiating particles in NLO calculations.

MoCaNLO uses consistently pole masses *M* and widths Γ, which are related to the complex poles μ via

$$ \mu ^2 = M^2 -\textrm{i}M \Gamma . $$

Since for the weak vector bosons usually the on-shell masses $M^\textrm{OS}$ and widths $\Gamma ^\textrm{OS}$ are quoted, these can be given alternatively using the input tags $\mathtt {<}  \texttt {ons\_z0\_mass}\mathtt {>}$, $\mathtt {<}  \texttt {ons\_z0\_width}\mathtt {>}$, $\mathtt {<}  \texttt {ons\_w\_mass}\mathtt {>}$, and $\mathtt {<}  \texttt {ons\_w\_width}\mathtt {>}$. From those, the pole masses and widths are calculated via [78]

$$ M = \frac{M^\textrm{OS}}{\sqrt{1+(\Gamma ^\textrm{OS}/M^\textrm{OS})^2}} \quad \text {and} \quad \Gamma = \frac{\Gamma ^\textrm{OS}}{\sqrt{1+(\Gamma ^\textrm{OS}/M^\textrm{OS})^2}}. $$

If no input values are provided, MoCaNLO determines the pole masses from default on-shell masses. Therefore, for weak vector bosons, pole masses and widths are always recomputed by MoCaNLO, unless directly specified via input. Note that pole and on-shell values can not be provided together in the param_card.xml. If so, the code stops, asking to correct the input.

In MoCaNLO, by default the $$G_\text {F}$$ scheme is used for the renormalisation of the EW coupling α. For $$\mathtt {<}  \texttt {masses\_in\_alpha\_gf}\mathtt {>}$$$${}=-1$$, the input value $$\mathtt {<}  \texttt {alphagf}\mathtt {>}$$ is used to set α. Otherwise, two cases are possible, where the value of α is derived from the value of $$G_\text {F}$$, set via $$\mathtt {<}  \texttt {fermi\_constant}\mathtt {>}$$, and from the masses of the W and Z bosons. For $$\mathtt {<}  \texttt {masses\_in\_alpha\_gf}\mathtt {>}$$$${}=0$$ (default), α is calculated with real masses via

$$ \alpha = \frac{\sqrt{2}G_\mu }{\pi }\mathop {\textrm{Re}}\nolimits {M_\text {W} ^2}\left( 1-\frac{\mathop {\textrm{Re}}\nolimits {M_\text {W} ^2}}{\mathop {\textrm{Re}}\nolimits {M_\text {Z} ^2}}\right) , $$

while for $$\mathtt {<}  \texttt {masses\_in\_alpha\_gf}\mathtt {>}$$$${}=1$$ complex masses are used according to

$$ \alpha = \frac{\sqrt{2}G_\mu }{\pi }\left| {M_\text {W} ^2}\left( 1-\frac{{M_\text {W} ^2}}{{M_\text {Z} ^2}}\right) \right| . $$

Additional input schemes for α are available and can be chosen using the input tag $$\mathtt {<}  \texttt {scheme\_alpha}\mathtt {>}$$. For $$\mathtt {<}  \texttt {scheme\_alpha}\mathtt {>}$$$${}=1$$ or $$\mathtt {<}  \texttt {scheme\_alpha}\mathtt {>}$$$${}=\texttt {gf}$$ the $$G_\mu $$ scheme is selected as discussed above. For $$\mathtt {<}  \texttt {scheme\_alpha}\mathtt {>}$$$${}=2$$ or $$\mathtt {<}  \texttt {scheme\_alpha}\mathtt {>}$$$${}=\texttt {alpha0}$$, the input value $$\mathtt {<}  \texttt {alpha0}\mathtt {>}$$ is used to set α, while for $$\mathtt {<}  \texttt {scheme\_alpha}\mathtt {>}$$$${}=3$$ or $$\mathtt {<}  \texttt {scheme\_alpha}\mathtt {>}$$$${}=\texttt {alphaz}$$, the input value $$\mathtt {<}  \texttt {alphaz}\mathtt {>}$$ is employed. Finally, for $$\mathtt {<}  \texttt {scheme\_alpha}\mathtt {>}$$$${}=4$$ or $$\mathtt {<}  \texttt {scheme\_alpha}\mathtt {>}$$$${}=\texttt {alphamsbar}$$, the $$\overline{{\text {MS}}}$$ scheme is chosen with α set to $$\mathtt {<}  \texttt {alpha\_starting\_value}\mathtt {>}$$ and the corresponding scale set to $$\mathtt {<}  \texttt {alpha\_starting\_scale}\mathtt {>}$$. The choice of $$\mathtt {<}  \texttt {scheme\_alpha}\mathtt {>}$$ fixes the input value of α and its renormalisation. For instance, in the $$\overline{{\text {MS}}}$$ scheme the counterterm in Eq. (427) of Ref. [26] is used. More details on the renormalisation of α can be found in the Recola manual [11]. Note that the use of differently renormalised couplings for external photons is steered via the parameters $$\mathtt {<}  \texttt {tree\_e0\_order}\mathtt {>}$$ in proc_card.xml.

In MoCaNLO, the value of the strong coupling constant $\alpha _\text {s}$ can be either provided by the user or calculated using its running. A fixed value of $\alpha _\text {s}$ can be set using the tag $\mathtt {<}  \texttt {alphas}\mathtt {>}$. The corresponding renormalisation scale $\mu _\text {R}$ is then assumed to be fixed and must be set via the tag $\mathtt {<}  \texttt {renormalization\_scale}\mathtt {>}$. Even when using a fixed $\alpha _\text {s}$ value, a running of this coupling is required if a scale variation is to be performed (see Sect. 6.2). For this reason, the value of $\mathtt {<}  \texttt {renormalization\_scale}\mathtt {>}$ corresponding to the given value $\mathtt {<}  \texttt {alphas}\mathtt {>}$ must be specified.

**Table 10:** Parameters of the $\mathtt {<}  \texttt {run\_parameters}\mathtt {>}$ section of param_card.xml that fix the kind of calculation and their defaults

Parameter	Possible values	Default value
$$\mathtt {<} \texttt {energy}\mathtt {>}$$		(Group)
$\mathtt {<} \texttt {cms\_beam\_energy}\mathtt {>}$	Positive real number	None
$\mathtt {<} \texttt {energy\_beam1}\mathtt {>}$	Positive real number	None
$\mathtt {<} \texttt {energy\_beam2}\mathtt {>}$	Positive real number	None
$$\mathtt {<} \texttt {scale}\mathtt {>}$$		(Group)
$\mathtt {<} \texttt {dynamical\_scale\_type}\mathtt {>}$	0, 1	0
$\mathtt {<} \texttt {scale\_factors\_f}\mathtt {>}$	List of real numbers	1.0
$\mathtt {<} \texttt {scale\_factors\_r}\mathtt {>}$	List of real numbers	1.0
$\mathtt {<} \texttt {n\_scales\_for\_histograms}\mathtt {>}$	Integer	Maximum # of scale factors
$$\mathtt {<} \texttt {pdfs}\mathtt {>}$$		(Group)
$\mathtt {<} \texttt {beam1}\mathtt {>}$	p,e+,e-,mu+,mu-	p
$\mathtt {<} \texttt {beam2}\mathtt {>}$	p,e+,e-,mu+,mu-	p
$\mathtt {<} \texttt {pdf\_set}\mathtt {>}$	See text	–
$\mathtt {<} \texttt {pdf\_set2}\mathtt {>}$	See text	–
$\mathtt {<} \texttt {alphas\_running}\mathtt {>}$	1, 2	See text
$\mathtt {<} \texttt {pdf\_scheme}\mathtt {>}$	String	See text
$\mathtt {<} \texttt {pdf\_scheme2}\mathtt {>}$	String	See text
$\mathtt {<} \texttt {pdf\_starting\_scale}\mathtt {>}$	Positive real number	$0.511\times 10^{-3}$
$\mathtt {<} \texttt {helicity\_em}\mathtt {>}$	-1,0,1	0
$\mathtt {<} \texttt {helicity\_ep}\mathtt {>}$	-1,0,1	0
$$\mathtt {<} \texttt {dipoles}\mathtt {>}$$		(Group)
$\mathtt {<} \texttt {alpha\_fs\_fs}\mathtt {>}$	Real ∈(0,1]	1.0
$\mathtt {<} \texttt {alpha\_fs\_is}\mathtt {>}$	Real ∈(0,1]	1.0
$\mathtt {<} \texttt {alpha\_is\_fs}\mathtt {>}$	Real ∈(0,1]	1.0
$\mathtt {<} \texttt {alpha\_is\_is}\mathtt {>}$	Real ∈(0,1]	1.0
$\mathtt {<} \texttt {DIS\_scheme}\mathtt {>}$	0, 1	0
$$\mathtt {<} \texttt {integration\_termination}\mathtt {>}$$		(Group)
$\mathtt {<} \texttt {n\_target\_accepted\_events}\mathtt {>}$	Integer ≥ 1000	$10^{15}$
$\mathtt {<} \texttt {max\_generated\_events}\mathtt {>}$	Integer	$10^{15}$
$\mathtt {<} \texttt {max\_vanishing\_events}\mathtt {>}$	Integer	100000
$\mathtt {<} \texttt {target\_relative\_precision}\mathtt {>}$	Integer	3
$\mathtt {<} \texttt {target\_absolute\_precision}\mathtt {>}$	Integer	12
$\mathtt {<} \texttt {time\_wall}\mathtt {>}$	days-hours:minutes	–

In general, MoCaNLO provides two options for the running of $\alpha _\text {s}$, controlled by the run parameter $\mathtt {<}  \texttt {alphas\_running}\mathtt {>}$ (see Sect. 6.2). If $\mathtt {<}  \texttt {alphas\_running}\mathtt {>}$ is set to 1, the running provided by LHAPDF is selected. If $\mathtt {<}  \texttt {alphas\_running}\mathtt {>}$ is set to 2, the running provided by Recola is instead used. In the latter case, a starting scale $Q_0$ and the value of the coupling $\alpha _\textrm{s}(Q^2_0)$ at that scale are required and can be specified via the parameters $\mathtt {<}  \texttt {alphas\_starting\_scale}\mathtt {>}$ and $\mathtt {<}  \texttt {alphas\_starting\_value}\mathtt {>}$. The running of the coupling also depends on the loop order of the running, fixed via the parameter $\mathtt {<}  \texttt {n\_loops}\mathtt {>}$, which can be 1 or 2. Note that MoCaNLO uses the five-flavour scheme.

As noted before, the running of the coupling is required even for a fixed scale if a scale variation is performed. Therefore, also in this case a value has to be given for $\mathtt {<}  \texttt {alphas\_running}\mathtt {>}$. The default value of $\mathtt {<}  \texttt {alphas\_running}\mathtt {>}$ depends on the parameters $\mathtt {<}  \texttt {beam1}\mathtt {>}$ and $\mathtt {<}  \texttt {beam2}\mathtt {>}$ (see Sect. 6.2). If at least one of the beams is a proton beam, $\mathtt {<}  \texttt {alphas\_running}\mathtt {>}$ defaults to 1, otherwise it is set to 2.

If fixed renormalisation and factorisation scales $\mu _\text {R}$ and $\mu _\text {F}$ are employed, their values must be passed using the tags $\mathtt {<}  \texttt {renormalization\_scale}\mathtt {>}$ and $\mathtt {<}  \texttt {factorization\_scale}\mathtt {>}$. If only one of the two scales is set by the user, MoCaNLO will automatically set both to the given value. If none of the two is set, they will both be assigned the value $M_\text {Z}$. The choice of a fixed or dynamical scale is determined by the run parameter $\mathtt {<}  \texttt {dynamical\_scale\_type}\mathtt {>}$ (see Sect. 6.2). Note that the IR scale $\mu _\text {IR}$ used for the computation of virtual corrections and integrated dipoles is not assumed to be equal to any of the previously defined scales in MoCaNLO and can be independently set via the $\mathtt {<}  \texttt {infrared\_scale}\mathtt {>}$ input. Since the result must not depend on $\mu _\text {IR}$, varying this parameter offers a useful consistency check on the calculation.

The technical parameters $m_\text {reg}$, *p*, and *q* enter the mappings of massless propagators and singularities in the subtraction terms in the MC integration. Their values can be adjusted via the tags $\mathtt {<}  \texttt {massless\_mapping\_mass}\mathtt {>}$, $\mathtt {<}  \texttt {massless\_mapping\_power}\mathtt {>}$, and $\mathtt {<}$subtraction_mapping_power$\mathtt {>}$, respectively. These influence the integration performance and may be tuned, but $m_\text {reg}$ should be smaller than the lowest physical scale of the process by several orders of magnitude, and p,q≲1 should hold.

Setting $\mathtt {<}  \texttt {neutrino\_species\_tagging}\mathtt {>}$ to 1 allows to switch on different jet types for neutrino species (see Table 4 in Sect. 5.1), which can be useful for tagging or producing histograms based on specific neutrino truth momenta. If this parameter is set to 0, all neutrinos have the same jet type (6).

The subsection $\mathtt {<}  \texttt {redefine\_jet\_types}\mathtt {>}$ allows to redefine the default $\mathtt {<}  \texttt {jet\_type}\mathtt {>}$ of a subset of particles. The jet types in the list $\mathtt {<}  \texttt {jet\_type}\mathtt {>}$ ...$\mathtt {<}  \texttt {/jet\_type}\mathtt {>}$ become the jet types of the corresponding particles in the list $\mathtt {<}  \texttt {jet\_particle}\mathtt {>}$ ...$\mathtt {<}  \texttt {/jet\_particle}\mathtt {>}$. In the example in Listing 15, the jet type of the antibottom quark is set to 27, which allows to recombine and tag them independently of the bottom quark and to plot corresponding observables. As a further example, the jet types of bottom and antibottom quarks or of the photon can be set to 1 if these shall be treated as light jets.

### Run parameters

The section $\mathtt {<}  \texttt {run\_parameters}\mathtt {>}$ of param_card.xml defines several parameters controlling the MC run. These parameters together with their possible input values are listed in Tables 10 and 11.

In Listing 16, run parameters for the NLO calculation of $\text {p} \text {p} \rightarrow \text {e} ^+\nu _\text {e} \text {b} \mu ^-\bar{\nu }_\mu \bar{\text {b}}$ are displayed as an example.

**Figure:**
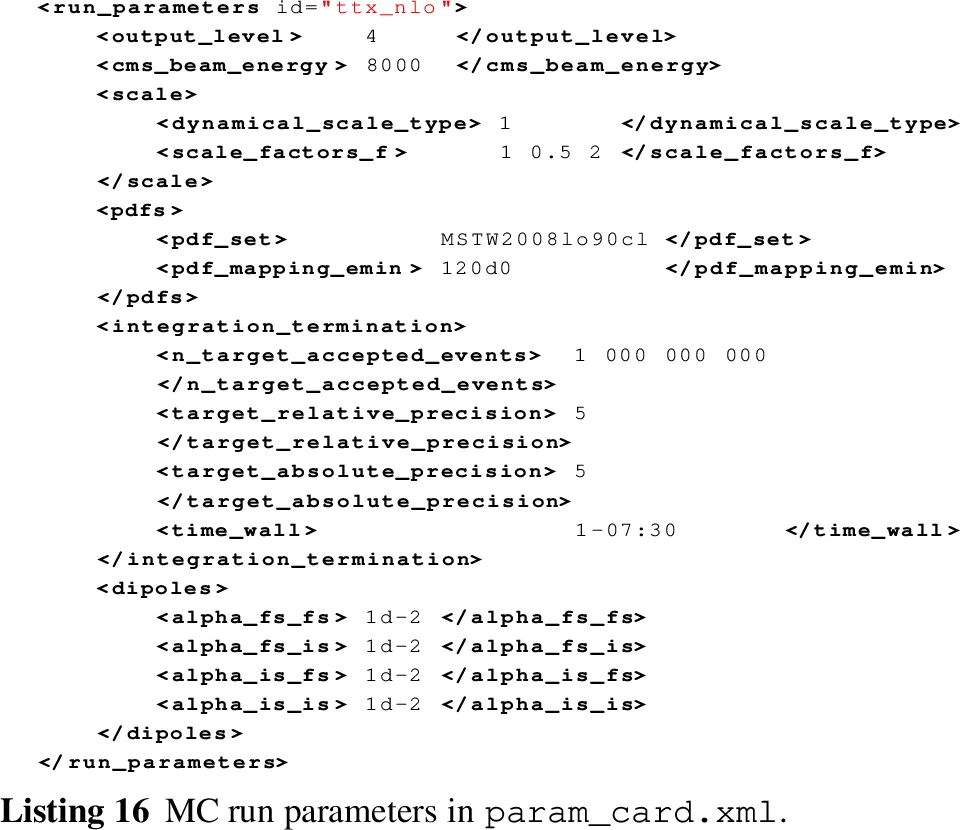

The run parameters can be grouped in param_card.xml into the subsections labelled $\mathtt {<}  \texttt {energy}\mathtt {>}$, $\mathtt {<}  \texttt {scale}\mathtt {>}$, $\mathtt {<}  \texttt {pdfs}\mathtt {>}$, $\mathtt {<}  \texttt {dipoles}\mathtt {>}$, $\mathtt {<}  \texttt {integration\_termination}\mathtt {>}$, $\mathtt {<}  \texttt {output}\mathtt {>}$, $\mathtt {<}  \texttt {intermediate\_output}\mathtt {>}$, $\mathtt {<}  \texttt {phase\_space\_generator}\mathtt {>}$, and $\mathtt {<}  \texttt {running\_options}\mathtt {>}$, but these tags are ignored by MoCaNLO.

Table 10 summarises the parameters that are needed to define the run.

The collider CMS energy is defined in $\mathtt {<}  \texttt {cms\_beam\_energy}\mathtt {>}$ in GeV. If this parameter is given, this energy is assumed to be equally distributed between both beams. For asymmetric colliders, the energies of the beams can be defined with $\mathtt {<}  \texttt {energy\_beam1}\mathtt {>}$ and $\mathtt {<}  \texttt {energy\_beam2}\mathtt {>}$ in GeV.
This subsection contains parameters related to the renormalisation and factorisation scales $$\mu _\text {R}$$ and $$\mu _\text {F}$$. The parameter $$\mathtt {<}  \texttt {dynamical\_scale\_type}\mathtt {>}$$ fixes the type of scales to be used. For $$\mathtt {<}  \texttt {dynamical\_scale\_type}\mathtt {>}$$$${}=0$$, fixed scales are chosen, whose values are passed via $$\mathtt {<}  \texttt {renormalization\_scale}\mathtt {>}$$ and $$\mathtt {<}  \texttt {factorization\_scale}\mathtt {>}$$ in the section $$\mathtt {<}  \texttt {model\_parameters}\mathtt {>}$$.
  Dynamical scales can be chosen by setting $\mathtt {<}$dynamical_scale_type$\mathtt {<}=1$. The definition of the scales itself must be specified in the file cut_card.xml (see Sect. 7.3 for details).
  The scales $\mu _\text {F}$ and $\mu _\text {R}$ can be independently re-scaled to perform an estimation of the scale uncertainty. The scaling factors are passed as lists of space-separated real numbers via the tags $\mathtt {<}  \texttt {scale\_factors\_f}\mathtt {>}$ and $\mathtt {<}  \texttt {scale\_factors\_r}\mathtt {>}$ for $\mu _\text {F}$ and $\mu _\text {R}$, respectively. The matrix elements and the PDFs are computed using the values of $\mu _\text {F}$ and $\mu _\text {R}$ as specified above times the first entry of $\mathtt {<}  \texttt {scale\_factors\_f}\mathtt {>}$ and $\mathtt {<}  \texttt {scale\_factors\_r}\mathtt {>}$, respectively. The first entry of each list corresponds to the central scale. Note that the runtime does not grow proportional to the number of scale factors. If the tags $\mathtt {<}  \texttt {scale\_factors\_f}\mathtt {>}$ and $\mathtt {<}  \texttt {scale\_factors\_r}\mathtt {>}$ are not provided, a single scaling factor equal to 1 is used for both scales. If only one list of factors is provided or if the lists have different lengths, the shorter list is extended to the length of the longer list using additional entries equal to 1.
  When a scale variation is performed, the results of each scale factor are written to different directories (see Sect. 3), whose names correspond to the position of the factors in the lists $\mathtt {<}  \texttt {scale\_factors\_f}\mathtt {>}$ and $\mathtt {<}  \texttt {scale\_factors\_r}\mathtt {>}$.
  The integer $\mathtt {<}  \texttt {n\_scales\_for\_histograms}\mathtt {>}$ defines for how many scale factors the histograms are computed at most (see Sect. 8), and its default value is the larger of the number of scales given in the lists $\mathtt {<}  \texttt {scale\_factors\_f}\mathtt {>}$ and $\mathtt {<}  \texttt {scale\_factors\_r}\mathtt {>}$. Histograms are produced for the first $\mathtt {<}  \texttt {n\_scales\_for\_histograms}\mathtt {>}$ factors in the lists $\mathtt {<}  \texttt {scale\_factors\_f}\mathtt {>}$ and $\mathtt {<}  \texttt {scale\_factors\_r}\mathtt {>}$. If $\mathtt {<}  \texttt {n\_scales\_for\_histograms}\mathtt {>}$ is larger than the length of the lists, the run stops.
This subsection contains parameters related to the incoming particles and the employed PDFs. The type of the beams is fixed via the input tags $\mathtt {<}  \texttt {beam1}\mathtt {>}$ and $\mathtt {<}  \texttt {beam2}\mathtt {>}$, which can take the values p, e+, e-, mu+ or mu-. By default, proton (p) beams are assumed.
  The PDF set is chosen via the tag $\mathtt {<}  \texttt {pdf\_set}\mathtt {>}$. For proton–proton collisions, the LHAPDF library is used (see Sect. 2).
  Different PDF sets for the second beam can be used by setting $\mathtt {<}  \texttt {pdf\_set2}\mathtt {>}$ to a value different from $\mathtt {<}  \texttt {pdf\_set}\mathtt {>}$. This way, proton–lepton collisions can be simulated. If $\mathtt {<}  \texttt {pdf\_set2}\mathtt {>}$ is not given, it is assumed to be equal to $\mathtt {<}  \texttt {pdf\_set}\mathtt {>}$.

**Figure:**
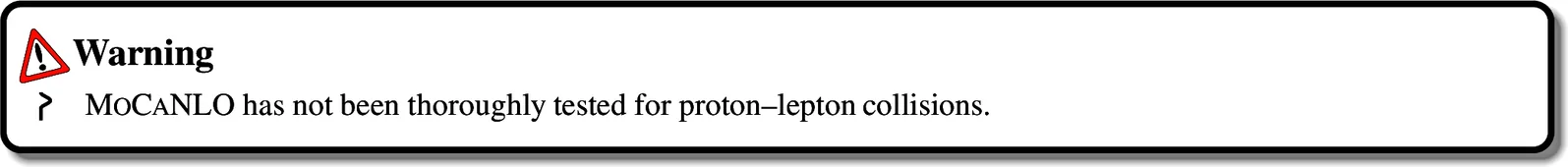

The tag $\mathtt {<}  \texttt {pdf\_scheme}\mathtt {>}$ fixes the subtraction scheme for the IR singularities in the collinear counterterms and should match the IR subtraction of the PDFs. Different schemes imply different finite contributions in the collinear counterterms. By default $\mathtt {<}  \texttt {pdf\_scheme}\mathtt {>}$ is set to MSbar, which is the only available setting for proton PDFs in MoCaNLO. Different settings are instead available for lepton PDFs, as discussed below.
  The strong coupling constant can either be taken from the PDFs or be calculated by Recola: This behaviour is controlled by the parameter $\mathtt {<}  \texttt {alphas\_running}\mathtt {>}$ in the $\mathtt {<}  \texttt {model\_parameters}\mathtt {>}$ of the param_card.xml (see Sect. 6.1). If $\mathtt {<}  \texttt {alphas\_running}\mathtt {>}$ is set to 1, $\alpha _\textrm{s}$ is obtained from the PDF set, if it is set to 2, $\alpha _\textrm{s}$ is calculated by Recola.
  The default value of $\mathtt {<}  \texttt {alphas\_running}\mathtt {>}$ depends on the parameters $\mathtt {<}  \texttt {beam1}\mathtt {>}$ and $\mathtt {<}  \texttt {beam2}\mathtt {>}$. If at least one of the beams is a proton beam, $\mathtt {<}  \texttt {alphas\_running}\mathtt {>}$ defaults to 1, otherwise it is set to 2.
  To simulate lepton–lepton collisions, the tag $\mathtt {<}  \texttt {pdf\_set}\mathtt {>}$ can be set to none. In this case, no PDFs are used and the initial-state collinear singularities are regularised with the electron or muon masses (see Sect. 6.1). Alternatively, MoCaNLO provides an internal implementation of LO lepton PDFs according to Appendix A of Ref. [79] including the $\mathcal {O}{\left( \alpha ^2\right) }$ and $\mathcal {O}{\left( \alpha ^3\right) }$ contributions as given in Appendix A of Ref. [80]. Following the nomenclature in Ref. [79], $\mathtt {<}  \texttt {pdf\_set}\mathtt {>}$ can be set to LO_beta, LO_eta, LO_mixed, and LO_collinear. The value of $\mathtt {<}  \texttt {pdf\_scheme}\mathtt {>}$ must be chosen accordingly and set to beta, eta, mixed, and collinear, respectively. The starting scale $\mu _0$ on which the LO_collinear case depends can be set via the input tag $\mathtt {<}  \texttt {pdf\_starting\_scale}\mathtt {>}$, which has the default value $0.511\times 10^{-3}$.

**Figure:**
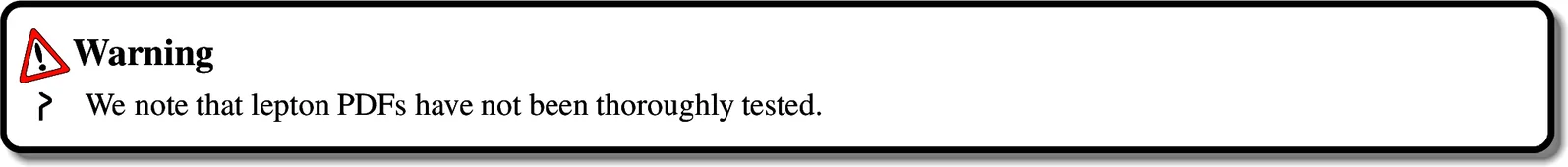

The parameters $\mathtt {<}  \texttt {helicity\_em}\mathtt {>}$ and $\mathtt {<}  \texttt {helicity\_ep}\mathtt {>}$ define the helicities of the incoming electrons and positrons, respectively. They can take the values ±1 for polarised beams. A value 0 corresponds to an unpolarised beam.
This section is only relevant for the real and integrated-dipole contributions. Here, the so-called α-parameters that control the phase-space volume of the subtraction and integrated dipoles can be set (see Ref. [81]). The α-parameters can be independently chosen using different input tags for some classes of emitter–emissus dipoles, specifically for final-state–final-state ($\mathtt {<}  \texttt {alpha\_fs\_fs}\mathtt {>}$), final-state–initial-state ($\mathtt {<}  \texttt {alpha\_fs\_is}\mathtt {>}$), initial-state–final-state ($\mathtt {<}  \texttt {alpha\_is\_fs}\mathtt {>}$), and initial-state–initial-state ($\mathtt {<}  \texttt {alpha\_is\_is}\mathtt {>}$) dipoles. The α-parameters can take values in the interval (0, 1] and their common default value is 1. Note that no α-parameters are available for subtraction dipoles related to internal resonances in the pole approximation.
  Corresponding real and integrated-dipole contributions must be calculated using the same value for the α-parameter, and the sum of these contributions must be independent of the specific value. Thus, the correctness of the subtraction procedure can be tested by varying the α-parameters.
  A further parameter controls the subtraction of collinear singularities. By choosing $$\mathtt {<}  \texttt {DIS\_scheme}\mathtt {>}$$$${}=1$$, the DIS factorisation scheme is switched on for photon PDFs, while the default is to use the $$\overline{{\text {MS}}}$$ factorisation scheme throughout. The flag $$\mathtt {<}  \texttt {DIS\_scheme}\mathtt {>}$$ has no effect for strongly interacting particles.
This section allows to set conditions for a regular termination of the integration. The integration terminates upon fulfilling at least one of the following conditions.
  The integration stops once the number of accumulated accepted, generated, or vanishing events exceeds the values given via the parameters $\mathtt {<}$n_target_accepted_events$\mathtt {>}$, $\mathtt {<}  \texttt {max\_generated\_events}\mathtt {>}$, and $\mathtt {<}$max_vanishing_events$\mathtt {>}$. Here, an event is vanishing when its weight is 0, while we refer to accepted events as those that pass the cuts (see Sect. 7.3). If the input value for $\mathtt {<}  \texttt {n\_target\_accepted\_events}\mathtt {>}$ is lower than 1000, it is replaced by 1000 and a corresponding message appears on the standard output. To ease legibility for the values of these three parameters, spaces can be used to separate groups of digits.
  A run is also terminated after a certain relative or absolute precision has been reached. These are specified with the tags $\mathtt {<}  \texttt {target\_relative\_precision}\mathtt {>}$ and $\mathtt {<}  \texttt {target\_absolute\_precision}\mathtt {>}$, whose values represent negative powers of ten. In Listing 16, for example, the target relative and absolute precisions are $10^{-5}$.
  Finally, the termination of the integration is triggered by exceeding the run time specified by the $\mathtt {<}  \texttt {time\_wall}\mathtt {>}$ parameter, whose value must have the format days-hours:minutes. This option can be useful when running on computer clusters with CPU-time limits. If no value is given to $\mathtt {<}  \texttt {time\_wall}\mathtt {>}$, this termination condition is not employed.

Table 11 summarises the parameters that control the MC integration and should only affect the results within integration errors.

**Table 11:** Elements of the $\mathtt {<}  \texttt {run\_parameters}\mathtt {>}$ section of param_card.xml affecting the details of the MC integration and their defaults

Parameter	Possible values	Default value
$\mathtt {<} \texttt {cms\_matrixelement}\mathtt {>}$	0, 1	1
$$\mathtt {<} \texttt {output}\mathtt {>}$$		(Group)
$\mathtt {<} \texttt {output\_level}\mathtt {>}$	2, 4, 6, 8	2
$\mathtt {<} \texttt {print\_feynman\_diagrams}\mathtt {>}$	true,false	true
$$\mathtt {<} \texttt {intermediate\_output}\mathtt {>}$$		(Group)
$\mathtt {<} \texttt {intermediate\_result\_start}\mathtt {>}$	Integer	1000
$\mathtt {<} \texttt {intermediate\_result\_stride}\mathtt {>}$	Integer	1000000
$\mathtt {<} \texttt {intermediate\_result\_factor}\mathtt {>}$	Positive real number	1.1
$$\mathtt {<} \texttt {phase\_space\_generator}\mathtt {>}$$		(Group)
$\mathtt {<} \texttt {use\_weight\_optimization}\mathtt {>}$	true,false	true
$\mathtt {<} \texttt {channel\_permutation}\mathtt {>}$	0, 1	0
$\mathtt {<} \texttt {channel\_elimination}\mathtt {>}$	0, 1	0
$\mathtt {<} \texttt {subtraction\_channels}\mathtt {>}$	0, 1	0
$\mathtt {<} \texttt {me\_virt\_veto\_threshold}\mathtt {>}$	Positive real number	None
$\mathtt {<} \texttt {pdf\_mapping\_emin}\mathtt {>}$	Positive real number	0.0
$\mathtt {<} \texttt {minimum\_relative\_invariant}\mathtt {>}$	Positive real number	$10^{-10}$
$\mathtt {<} \texttt {cos\_theta\_tolerance}\mathtt {>}$	Positive real number	$10^{-10}$
$\mathtt {<} \texttt {tmax\_tolerance}\mathtt {>}$	Positive real number	$6.4\times 10^{-3}$
$$\mathtt {<} \texttt {momentum\_conservation\_tolerance}\mathtt {>}$$	Positive real number	$$10^{-12} $$
$\mathtt {<} \texttt {onshellness\_tolerance}\mathtt {>}$	Positive real number	$10^{-12}$
$$\mathtt {<} \texttt {running\_options}\mathtt {>}$$		(Group)
$\mathtt {<} \texttt {store\_run\_resuming\_info}\mathtt {>}$	0, 1	0
$\mathtt {<} \texttt {writing\_mode}\mathtt {>}$	0, 1	0
$\mathtt {<} \texttt {load\_run\_resuming\_info}\mathtt {>}$	0, 1	0
$\mathtt {<} \texttt {folder\_overwriting}\mathtt {>}$	0, 1, 2	0

The matrix elements can be calculated either in the laboratory system or with momenta boosted to the CM frame to improve numerical accuracy. This behaviour is controlled by the parameter $$\mathtt {<}  \texttt {cms\_matrixelement}\mathtt {>}$$. By default, $$\mathtt {<}  \texttt {cms\_matrixelement}\mathtt {>}$$$${}=1$$ and the CM-frame momenta are employed. By setting the $$\mathtt {<}  \texttt {cms\_matrixelement}\mathtt {>}$$$${}=0$$, the use of laboratory frame momenta can be enforced.

**Figure:**
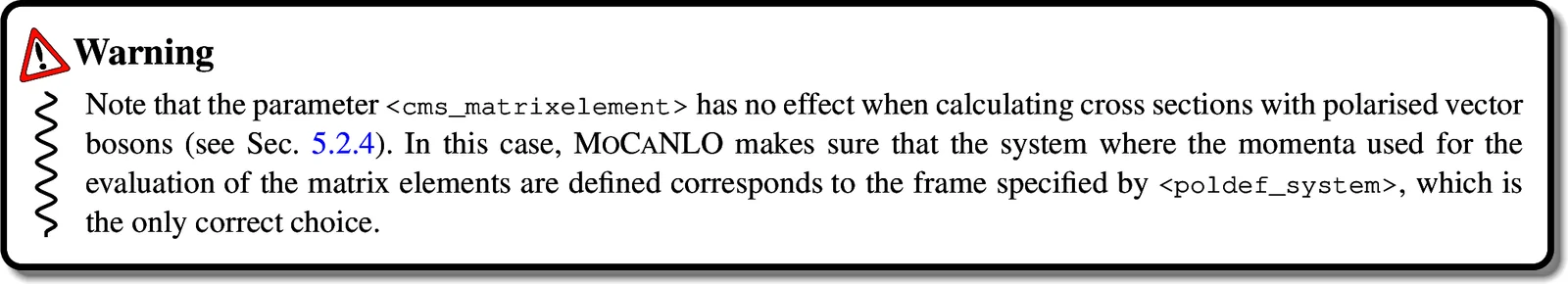

The parameter $$\mathtt {<}  \texttt {output\_level}\mathtt {>}$$ controls the amount of information for the standard output. The values $$\mathtt {<}  \texttt {output\_level}\mathtt {>}$$$${}=2,4,6,8$$ correspond to minimal, intermediate, maximal relevant, and complete output, respectively. By default, MoCaNLO prints two files collecting all Feynman diagrams that were used to construct the integration channels. The file feynman_diagrams.tex in the subdirectory run.../data/init contains the channels sorted in the order they were generated, while the file feynman_diagrams_survey.tex in the subdirectory run.../data/run contains them sorted by their importance in the integration. For complicated processes, these files can become very large and their writing very time consuming. The writing of these files can be switched off by issuing $$\mathtt {<}  \texttt {print\_feynman\_diagrams}\mathtt {>}$$$${}={}$$false.
The parameters of this section control when intermediate results of the integration are written out. The parameter $\mathtt {<}$intermediate_result_start$\mathtt {>}$ specifies after how many accepted events this occurs for the first time. Its default value is 1000. After the first time, intermediate results are written whenever the number of accepted events has increased by the value of $\mathtt {<}  \texttt {intermediate\_result\_stride}\mathtt {>}$, with default 1000000, or by a factor corresponding to the value of $\mathtt {<}  \texttt {intermediate\_result\_factor}\mathtt {>}$, with default 1.1.
This section includes parameters that are relevant for the phase-space generation. The parameter $\mathtt {<}  \texttt {use\_weight\_optimization}\mathtt {>}$ turns on or off the use of MoCaNLO ’s adaptive multi-channel weight-optimisation algorithm and should normally be set to true.

Several flags exist to control the selection of integration channels:

- If $$\mathtt {<}  \texttt {channel\_permutation}\mathtt {>}$$$${}=1$$, extra integration channels with permuted resonances are included. This is needed for processes with nested resonances, for example.
- If $$\mathtt {<}  \texttt {channel\_elimination}\mathtt {>}$$$${}=1$$, integration channels that differ from others only by internal non-resonant lines are eliminated. This can be useful to reduce the number of channels, in particular if $$\mathtt {<}  \texttt {channel\_permutation}\mathtt {>}$$$${}=1$$.
- The flag $\mathtt {<}  \texttt {subtraction\_channels}\mathtt {>}$ is relevant only for real contributions. If its value is 1, extra integration channels corresponding to Catani–Seymour dipole subtraction contributions are taken into account. This can significantly increase the total number of channels. Note that no extra channels are available for subtraction dipoles related to internal resonances in the pole approximation.

The real parameter $\mathtt {<}  \texttt {me\_virt\_veto\_threshold}\mathtt {>}$ can be set to discard events with unusually large virtual matrix elements, i.e. $2\mathop {\textrm{Re}}\nolimits \,\mathcal {M}_\text {virt}\mathcal {M}_\text {Born}^* >{}$ $\mathtt {<}$me_virt_veto_threshold$\mathtt {>}{}\cdot |\mathcal {M}_\text {Born}|^2$. Such events might result from phase-space points that are problematic for Collier, but whose removal does not impact the final result, as long as their number is limited. It is the user’s responsibility to ensure that only a few events are discarded when this parameter is activated, for instance by inspecting the output file cross_section.dat in the result/ folder of the run.

A minimal partonic CM energy can be required to avoid the generation of events which would be cut away anyhow. This value can be specified via the parameter $\mathtt {<}  \texttt {pdf\_mapping\_emin}\mathtt {>}$. We note that MoCaNLO also computes a minimal partonic CM energy from the transverse-momentum cuts provided via the cut_card.xml file (see Sect. 7.3). The value that is actually employed is the larger of the two. It needs to be larger than zero, otherwise the code stops with an error message.

The parameters $\mathtt {<}  \texttt {minimum\_relative\_invariant}\mathtt {>}$, $\mathtt {<}  \texttt {cos\_theta\_tolerance}\mathtt {>}$, $\mathtt {<}  \texttt {tmax\_tolerance}\mathtt {>}$, $\mathtt {<}$momentum_conservation_tolerance$\mathtt {>}$, and $\mathtt {<}$onshellness_tolerance$\mathtt {>}$ are technical cut parameters of the MC integration that avoid the appearance of very large unphysical weights. They can be varied (typically by factors of 10) about their default values to verify that the integration results are independent of these parameters.

This subsection contains a set of parameters that control the storing and loading of information during a computation, which can be used to resume a run after a walltime has been reached, for example. A description of the parameters in this subsection is given in Sect. 6.2.1.

#### How to perform sequential runs

MoCaNLO is capable of loading the results of a previous run and restarting a calculation that has met a termination criterion, provided the necessary information has been stored. In combination with the time_wall parameter (part of the $\mathtt {<}  \texttt {integration\_termination}\mathtt {>}$ subsection of param_card.xml), this feature can be used to perform a resumption chain in which each step is within the walltime of a cluster, but which effectively corresponds to a longer integration runtime. By using this method, a smaller integration error can be achieved with the same CPU time compared to parallel job execution. This is because the optimised channel weights are inherited by subsequent steps of the resumption chain, whereas parallel runs perform the same channel weight optimisation independently. We remark that parallel and sequential running can be combined by simply computing parallel resumption chains.

The $\mathtt {<}  \texttt {running\_options}\mathtt {>}$ subsection of the $\mathtt {<}$run_parameters$\mathtt {>}$ section in the file param_card.xml contains a set of parameters that control the storing and loading of information during a computation. Listing 17 displays the complete set of parameters of this subsection.

**Figure:**
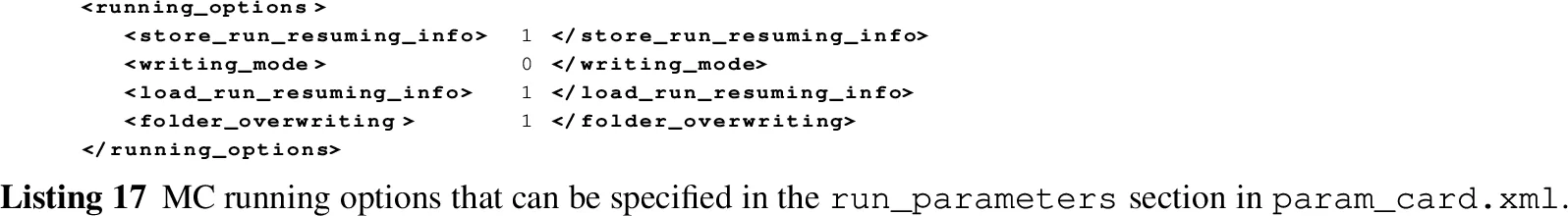

Their possible and default values are found in the lower part of Table 11.

Setting $$\mathtt {<}  \texttt {store\_run\_resuming\_info}\mathtt {>}$$$${}=1$$ enables the storage of the information necessary to resume that run later. If $$\mathtt {<}  \texttt {store\_run\_resuming\_info}\mathtt {>}$$$${}=0$$, no storage will take place.

The parameter $$\mathtt {<}  \texttt {writing\_mode}\mathtt {>}$$ controls at which points of the run the storage of information necessary for resumption takes place. By default ($$\mathtt {<}  \texttt {writing\_mode}\mathtt {>}$$$${}=0$$), the storage occurs when a MoCaNLO termination condition is met (see subsection $$\mathtt {<}  \texttt {integration\_termination}\mathtt {>}$$ in Sect. 6.2). If $$\mathtt {<}  \texttt {writing\_mode}\mathtt {>}$$$${}=1$$, the storage occurs each time MoCaNLO writes intermediate results (see the beginning of Sect. 3.3).

Setting $$\mathtt {<}  \texttt {load\_run\_resuming\_info}\mathtt {>}$$$${}= 1$$ tells the program that the user intends to resume a run and that it should load the necessary information. In this case, the run directory of the run that is to be resumed must be passed as an argument (see the example below for further details). If $$\mathtt {<}  \texttt {load\_run\_resuming\_info}\mathtt {>}$$$${}= 0$$ (default), no loading will take place. By choosing both $$\mathtt {<}  \texttt {load\_run\_resuming\_info}\mathtt {>}$$$${}= 1$$ and $$\mathtt {<}  \texttt {store\_run\_resuming\_info}\mathtt {>}$$$${}=1$$, intermediate steps in a chain of resumptions can be performed.

The parameter $$\mathtt {<}  \texttt {folder\_overwriting}\mathtt {>}$$ determines how the results of a resumption run are stored, which is relevant for subsequent run resumptions. The default value $$\mathtt {<}  \texttt {folder\_overwriting}\mathtt {>}$$$${}= 0$$ makes a backup of the results of the run that will be resumed and stores the results of the resumption under the name of the original run directory. If one sets $$\mathtt {<}  \texttt {folder\_overwriting}\mathtt {>}$$$${}= 1$$, the results of previous steps of a resumption chain are kept, but the results of a step are overwritten if that step is performed again. Finally, the value $$\mathtt {<}  \texttt {folder\_overwriting}\mathtt {>}$$$${}= 2$$ prevents any overwriting, so that the results of all intermediate and parallel steps are kept. An exemplary usage of this parameter can be found at the end of this subsection.

In the following, details on the storage and resumption procedures are given using the example introduced in Sect. 3.3. There, a MoCaNLO run for a specific subprocess was started

**Figure:**
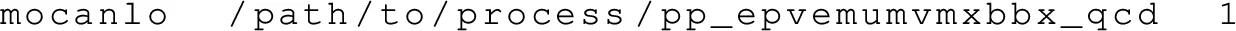

where the two arguments are the path to the process directory, which contains the cards directory, and the run ID of the subprocess. If this run is performed with $$\mathtt {<}  \texttt {store\_run\_resuming\_info}\mathtt {>}$$$${}=0$$, the run’s output directory contains the data subdirectory, with information about the initialisation and the run phase, as well as the result subdirectory, where cross sections and distributions are saved. If the information storage is activated by setting $$\mathtt {<}  \texttt {store\_run\_resuming\_info}\mathtt {>}$$$${}=1$$, an additional subdirectory named carbon_copy is created:

**Figure:**
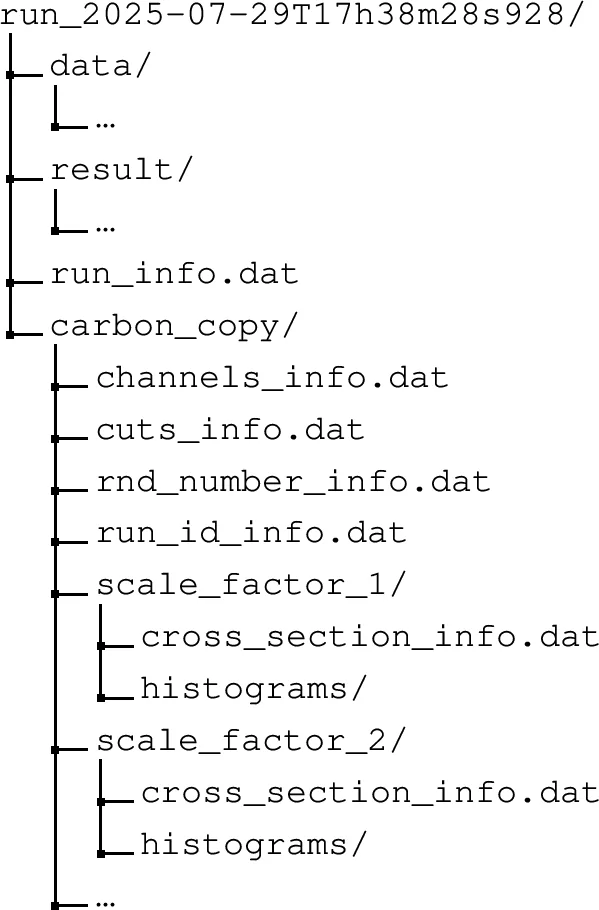

The files in carbon_copy contain the required information for the run resumption. In the file run_id_info.dat general information on how the run was performed is written, like its run_type, run_parameters, and any other tag specified for the run ID in the run_card.xml. This information is used when resuming a run to check that the code is restarted with the same set of parameters.

In rnd_number_info.dat, a set of 25 random numbers is stored that uniquely specifies the current point in the RANLUX pseudo-random number sequence [82]. Using this information, the event generation chain can restart from the exit point of the previous run. In channels_info.dat, information on the integration channels is stored, like the weight-optimisation step, together with the channels’ weights and variances, the respective contribution to the integrals and so on. This way, the program can benefit from a run in which an optimisation of the weights has occurred.

In order to consistently restore a run, information on the hits of the different cuts specified in cut_card.xml is saved in cuts_info.dat. This allows the cumulative counting of the hits for each cut.

Depending on the number of scales specified by $\mathtt {<}  \texttt {scale\_factors\_f}\mathtt {>}$ and $\mathtt {<}  \texttt {scale\_factors\_r}\mathtt {>}$, an equal number of scale subdirectories is stored within the carbon_copy subdirectory (if only one scale is provided, no additional scale directory is produced). Therein, one can find all information to restart the cross section computation at the integrated level, in cross_section_info.dat, and even differentially, in the subdirectory histograms, containing information for all required distributions in separate files named after the observable name specified by the user. Moreover, in cross_section_info.dat, the maximum weight and the sum of the absolute weights computed so far are also contained.

In order to resume a previous run that contains a carbon_copy subdirectory, like the one of our example, whose output has been stored in run_2025-07-29T17h38m28s928, one has to set the parameter $\mathtt {<}  \texttt {load\_resuming\_run\_info}\mathtt {>}$ to 1 and start a new run as

**Figure:**
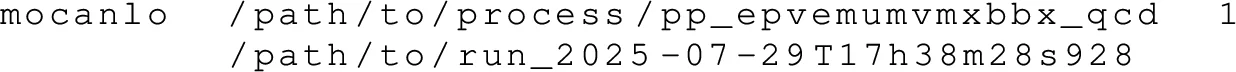

where the second argument is the run ID of the subprocess in question and the third argument is the path to the directory that contains the results of the run to be resumed.

An additional argument containing the random number seed before or after the name of the directory to resume is tolerated but not used, since all initialisation conditions for the random number chain and the integral computation are loaded from run_2025-07-29T17h38m28s928 /carbon_copy. If a run is restarted with a different set of relevant parameters, the code stops with an error message pointing to a file containing the conflicting lines of the cards that triggered the error. This file is located in the subdirectory data/init/user_input/ of the current run and named diff_user_input_ *section*.dat, where *section* can be cuts, histograms, monte_carlo, on_shell_projection, partonic_process, recombinations, resonances, scales, standard_model, or weight_opts depending on which section caused the error. These files are temporary and are removed if the run resumption succeeds. The parameters contained in the subsections $\mathtt {<}  \texttt {running\_}\mathtt {>}$ $\mathtt {<}  \texttt {options}\mathtt {>}$ and $\mathtt {<}  \texttt {integration\_termination}\mathtt {>}$ of param_card.xml are ignored when comparing the input parameters of subsequent runs, so that their values can change without triggering an error.

The options $\mathtt {<}  \texttt {load\_run\_resuming\_info}\mathtt {>}$ and $\mathtt {<}$store_run_resuming_info$\mathtt {>}$ can be activated at the same time to perform chains of run resumptions. Depending on the value of $\mathtt {<}  \texttt {folder\_overwriting}\mathtt {>}$ (discussed above), the name of the directory containing the results of a run resumption might not contain the time at which the directory was created, as opposed to the case of a fresh run. Therefore, this information is printed into a run_info.dat file, together with the name of the directory from which the data for the resumption was loaded. This file can be found directly in the directory of the resuming run, at the same level of the data, results and carbon_copy subdirectories.

The parameter $\mathtt {<}  \texttt {folder\_overwriting}\mathtt {>}$ controls the location of the output of a resumed run. Setting $\mathtt {<}$folder_overwriting$\mathtt {>}=2$ and executing the restart command above, the results of the restarted run are located in run_2025-07-29T17h38m28s928. Note that the name of this run directory comprises the original directory’s name from which the data have been loaded, the number of times N the run has been resumed (as stepN), and a new time stamp with the time at which the run has been restarted. By executing again the restart command above, a directory with a different time stamp, e.g. run_2025-07-29T17h38m28s928 _step1_2025-07-30T18h14m01s811, is created, containing results from a new resumption of the same run, thus carrying a different (second) timestamp.

If the flag $\mathtt {<}  \texttt {store\_run\_resuming\_info}\mathtt {>}$ was set to 1, this run can be further resumed by running

**Figure:**
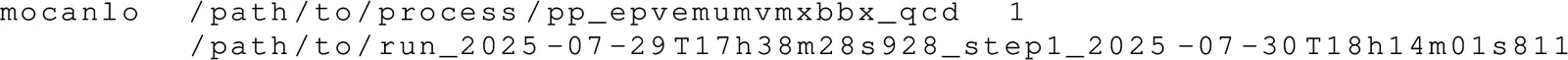

which creates run_2025-07-29T17h38m28s928 _step2_2025-07-31T18h17m28s388 directory, for instance. Note that the timestamp of the original run (step 0) is kept in the name at the first position, while the second timestamp corresponds to the last performed step (step2 in this case). The directory run_2025-07-29T17h38m28s928 _step1_2025-07-30T18h14m01s811, which contains the results of the intermediate step1, as well as the directory run_2025-07-29T17h38m28s928_step1_2025-07-30T17h42m25s330, which contains the results of a step1 that were not used for a run resumption, remain.

In order to avoid the proliferation of intermediate-result directories, $$\mathtt {<}  \texttt {folder\_overwriting}\mathtt {>}$$ can be set to 1. This causes the result directory of stepN to be replaced if a new stepN, i.e.  the same step, is performed using the same loaded information. This option also affects the name of the result directories, in that no second timestamp is included. In our example, setting $$\mathtt {<}  \texttt {folder\_overwriting}\mathtt {>}$$$${}=1$$ and executing the first restart command stores the results into a folder run_2025-07-29T17h38m28s928 _step1. Re-executing the command again will overwrite the step1 directory. Resuming the step1 of the run with the command

**Figure:**
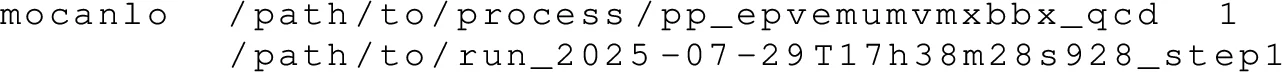

creates a directory run_2025-07-29T17h38m28s928 _step2 and so on. The directory run_2025-07-29T17h38m28s928_step1 remains. Therefore, only the most recent results of each step are stored.

By setting $$\mathtt {<}  \texttt {folder\_overwriting}\mathtt {>}$$$${}=0$$, the storage can be restricted to the two directories containing the results of the last two steps. By executing the first restart command, the original directory run_2025-07-29T17h38m28s928 is renamed into run_2025-07-29T17h38m28s928_bckp and the results of this first resumption are saved under the previous name of the original directory, i.e. run_2025-07-29T17h38m28s928. Issuing the restart command again, the second step is performed and its results are saved under the name run_2025-07-29T17h38m28s928, while run_2025-07-29T17h38m28s928 _bckp now contains the results of the first step.

#### Photon–photon ultra-peripheral collisions

MoCaNLO is able to simulate initial-state photons originating from heavy ions in ultra-peripheral collisions. To that end, it relies on the external program gamma-UPC [71].

To steer all the possible options of the library, several flags are available in param_card.xml in the $\mathtt {<}  \texttt {pdfs}\mathtt {>}$ section.

**Table 12:** Additional parameters for ultra-peripheral collisions in the param_card.xml and their default values

Parameter	Possible values	Default value
$\mathtt {<} \texttt {alpha\_UPC}\mathtt {>}$	Positive real number	1/137.036
$\mathtt {<} \texttt {UPC\_photon\_flux}\mathtt {>}$	0, 1	0
$\mathtt {<} \texttt {nuclear\_A\_beam1}\mathtt {>}$	Integer	208
$\mathtt {<} \texttt {nuclear\_A\_beam2}\mathtt {>}$	Integer	208
$\mathtt {<} \texttt {nuclear\_Z\_beam1}\mathtt {>}$	Integer	82
$\mathtt {<} \texttt {nuclear\_Z\_beam2}\mathtt {>}$	Integer	82

An example to run Pb–Pb collisions using the electric-dipole form factor (EDFF) [83] (instead of the charge (ChFF) form factor [84]) is:

**Figure:**
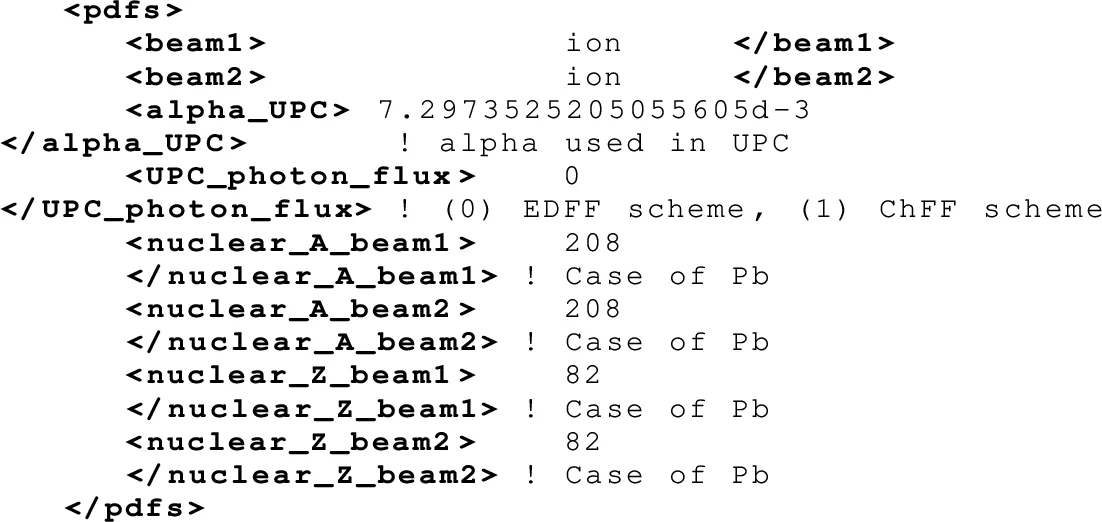

The value of α used in UPC can be changed independently of the one used in the matrix element. The default values of the UPC parameters can be found in Table 12.

### Setting a-priori weights for the Monte Carlo integration

By default, the a-priori weights of the different MC integration channels are all set to the same value, equal to the inverse of the total number of integration channels for the process of interest. They represent initialisation values for the multi-channel optimisation, which adapts them during the integration [85]. In order to improve the convergence of the result for processes dominated by diagrams containing resonances, it can be useful to tune the a-priori weights at initialisation. This can be achieved by linking in the run_card.xml a section , where XX is an ID that denotes a particular a-priori weight setting. This section contains one or more subsections $\mathtt {<}  \texttt {weight\_opt}\mathtt {>}$ that independently specify the information on the resonant contributions to be enhanced. Therefore, the a-priori weights of the integration channels corresponding to Feynman diagrams containing at least $\mathtt {<}  \texttt {min\_n\_resonant\_particles}\mathtt {>}$ and at most $\mathtt {<}  \texttt {max\_n\_resonant\_particles}\mathtt {>}$ particles specified by $\mathtt {<}  \texttt {particle}\mathtt {>}$ as an *s*-channel resonance are adjusted. They are multiplied by a factor $\mathtt {<}  \texttt {mult\_factor}\mathtt {>}$ for each resonance found in the associated Feynman diagram. The global tag $\mathtt {<}  \texttt {total\_max\_n\_resonant\_particles}\mathtt {>}$ limits the total number of enhancement factors to the specified number. Thus, a channel with more resonances than $\mathtt {<}  \texttt {total\_max\_n\_resonant\_particles}\mathtt {>}$ is not enhanced at all.

An exemplary application is shown in Listing 18 for $\text {t} \text {Z} \text {j}$ production, where a-priori weights can be enhanced by up to a factor of 30, i.e. by a factor of 10 if they belong to integration channels corresponding to diagrams with exactly one top quark, and by an additional factor of three if their integration channels also include one $\text {Z}$ boson. Note that in the example there are no a-priori weights that can be multiplied by a factor higher than 30, as the global tag $\mathtt {<}  \texttt {total\_max\_n\_resonant\_particles}\mathtt {>}$ limits the number of enhancement factors to 2. Therefore, there are four distinct situations in which a-priori weights can be enhanced: for channels with one top quark (multiplied by 10), one Z boson (multiplied by 3), two Z bosons (multiplied by 9), and one top quark and one Z boson (multiplied by 30).

**Figure:**
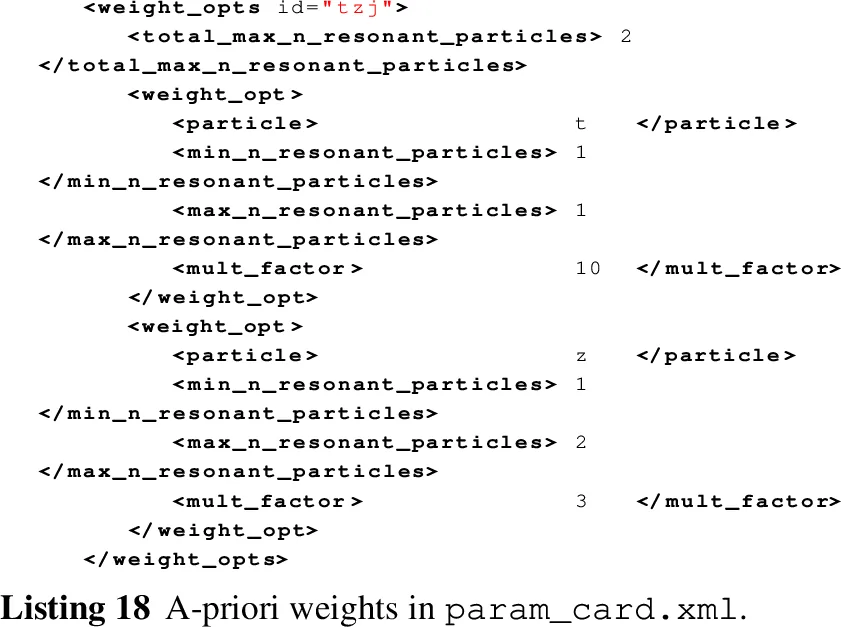

Few additional comments on the $\mathtt {<}  \texttt {particle}\mathtt {>}$ tag are in order. First of all, note that particles and antiparticles are distinguished. In order to treat them on the same footing, the MoCaNLO identifier of the resonance (see second column of Table 4) must be accompanied by a star key, i.e. t* indicates either a t or a , and similarly w* either a w+ or a w-. Moreover, a list of particles can be specified in $\mathtt {<}  \texttt {particle}\mathtt {>}$: particles in the list are treated on the same level as far as the assignment of an enhancement factor of the a-priori weight is concerned.

## Recombination, cut, and scale definitions: the cut_card.xml

The cut_card.xml defines the sections $\mathtt {<}  \texttt {recombinations}\mathtt {>}$, $\mathtt {<}  \texttt {cuts}\mathtt {>}$, and $\mathtt {<}  \texttt {scales}\mathtt {>}$ for setting the recombination parameters for the clustering algorithm, defining the acceptance function, and fixing the dynamical scales, respectively.

After recombination, clustered objects are stored in *sorted clusters*, which consist of lists of different jet types, each ordered by transverse momentum (see Sect. 7.3). The cut procedure operates on these sorted clusters by modifying and extending them. The definition of scales and of observables of histograms is based on the sorted clusters that result from the cut procedure.

### Recombinations

The settings in the section $\mathtt {<}  \texttt {recombinations}\mathtt {>}$ of the cut_card.xml configure the jet algorithm. The scheme allows for different steps of recombinations, which can be executed consecutively or in parallel. The recombination may involve several levels, at which the parallel recombinations are merged. The input of the recombination is the final-state particles of the considered process, while the output is a list of clusters, to be used as input for the cut routines. Note that the final list of clusters includes only objects that pass through all recombination levels, even if they are not subject to recombination.

The parameters, with their possible and default values, of the section $\mathtt {<}  \texttt {recombinations}\mathtt {>}$, the subsections $\mathtt {<}  \texttt {recombination\_step}\mathtt {>}$, and their subsections $\mathtt {<}  \texttt {recombination}\mathtt {>}$ are listed in Tables 13, 14, and 15, respectively.

A simple example with only one recombination step and thus one recombination level is shown in Listing 19,

**Figure:**
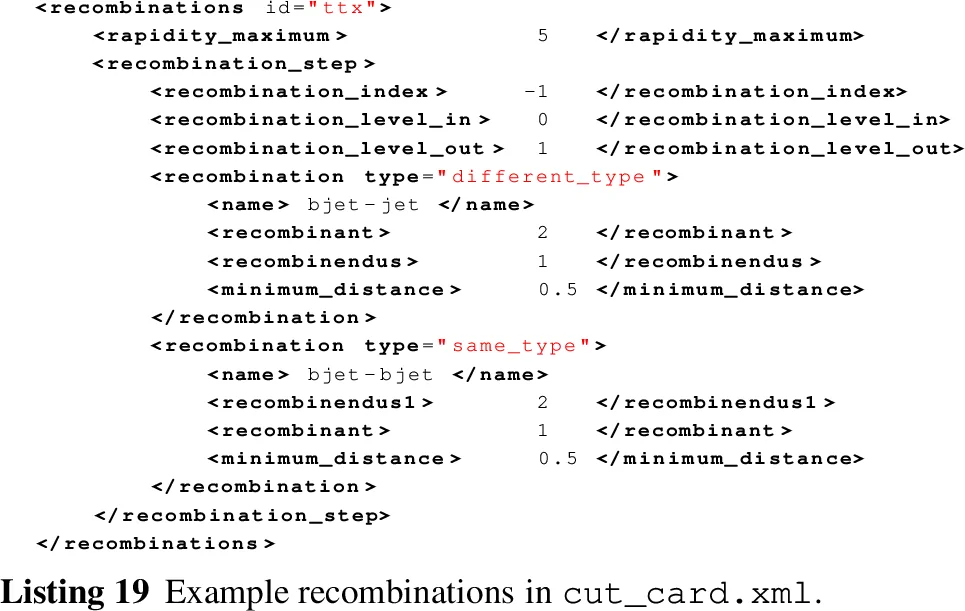

while a more complicated example with three recombination steps and two recombination levels is given in Listing 20.

**Figure:**
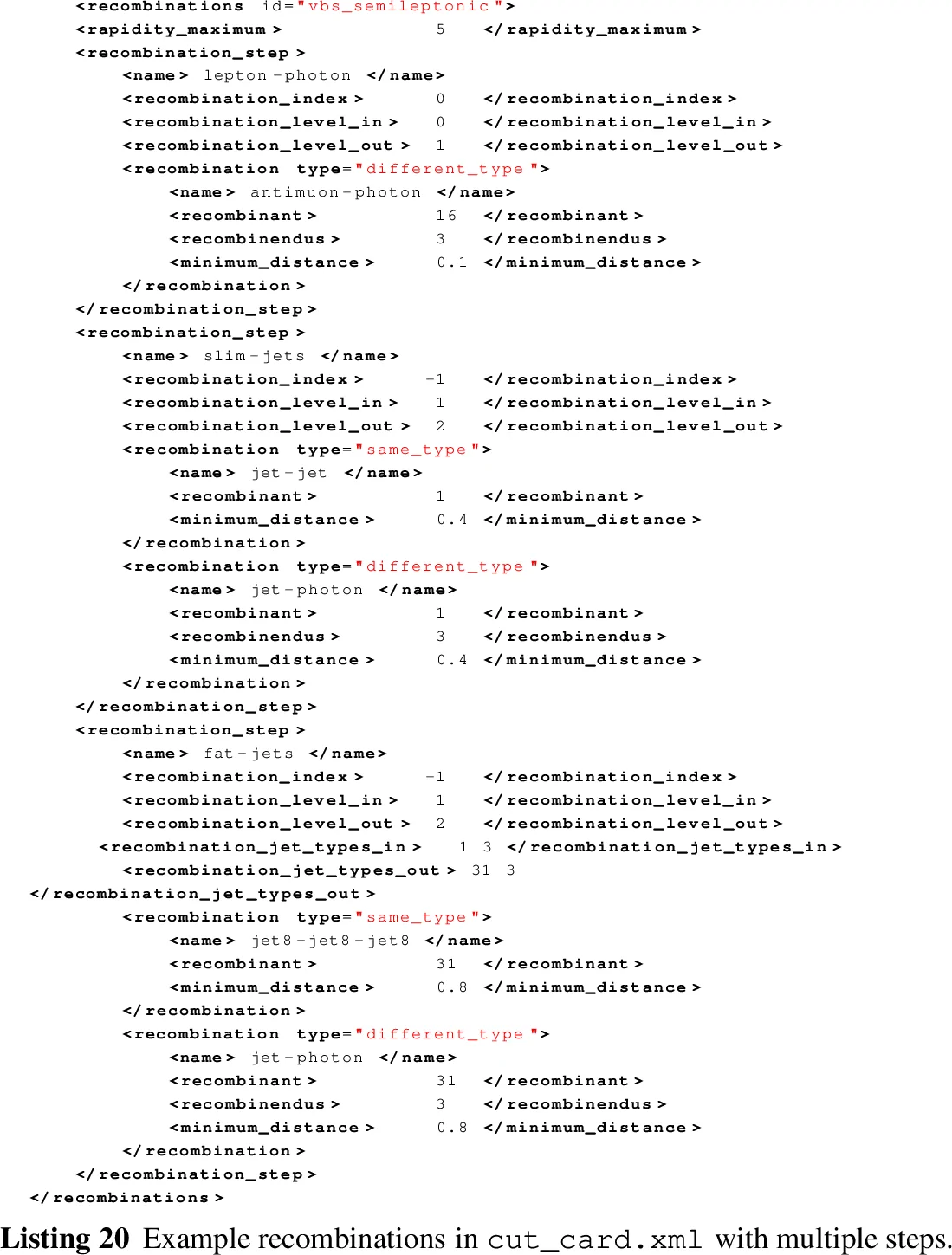

The section , linked via the ID in the run_card.xml, contains one or more $\mathtt {<}  \texttt {recombination\_step}\mathtt {>}$ subsections as well as the further parameters $\mathtt {<}  \texttt {rapidity\_maximum}\mathtt {>}$ and $\mathtt {<}$recombination_index$\mathtt {>}$. Particles with a rapidity larger than $\mathtt {<}$rapidity_maximum$\mathtt {>}$ are disregarded in the recombination algorithm. Since neutrinos do not undergo any recombination, they are not affected by it. The parameter $\mathtt {<}  \texttt {recombination\_index}\mathtt {>}$ defines the default clustering algorithm and takes the values -1, 0, and 1 for the anti-$k_\textrm{t}$ [86], the Cambridge–Aachen [87, 88], and the $k_\textrm{t}$ algorithm [89, 90], respectively.

The parameters of a $\mathtt {<}  \texttt {recombination\_step}\mathtt {>}$ are summarised in Table 14. The optional tag $\mathtt {<}  \texttt {name}\mathtt {>}$ is only used for output purposes. The optional parameter $\mathtt {<}$recombination_index$\mathtt {>}$ allows to overwrite, for the corresponding $\mathtt {<}  \texttt {recombination\_step}\mathtt {>}$, the value of $\mathtt {<}$recombination_index$\mathtt {>}$ defined for the  section, discussed above. Note that only one algorithm can be chosen within a single $\mathtt {<}  \texttt {recombination\_step}\mathtt {>}$.

**Table 13:** Parameters and possible values of the $\mathtt {<}  \texttt {recombinations}\mathtt {>}$ section

Parameter	Possible values	Default value
$\mathtt {<} \texttt {rapidity\_maximum}\mathtt {>}$	Real number	100.0
$\mathtt {<} \texttt {recombination\_index}\mathtt {>}$	Real number	-1
$\mathtt {<} \texttt {recombination\_step}\mathtt {>}$	–	–

**Table 14:** Parameters and possible values of the $\mathtt {<}  \texttt {recombinations\_step}\mathtt {>}$ section

Parameter	Possible values	Default value
$\mathtt {<} \texttt {name}\mathtt {>}$	String	None
$\mathtt {<} \texttt {recombination\_index}\mathtt {>}$	Real number	-1
$\mathtt {<} \texttt {recombination\_level\_in}\mathtt {>}$	Non-negative integer	0
$\mathtt {<} \texttt {recombination\_level\_out}\mathtt {>}$	Positive integer	1
$\mathtt {<} \texttt {recombination\_jet\_types\_in}\mathtt {>}$	List of jet types	None
$\mathtt {<} \texttt {recombination\_jet\_types\_out}\mathtt {>}$	List of jet types	None
$\mathtt {<} \texttt {recombination}\mathtt {>}$	–	–

**Table 15:** Parameters and possible values of the $\mathtt {<}  \texttt {recombination}\mathtt {>}$ section

Parameter	Possible values	Default value
$\mathtt {<} \texttt {name}\mathtt {>}$	String	None
$\mathtt {<} \texttt {recombinant}\mathtt {>}$	Integer	0
$\mathtt {<} \texttt {recombinendus}\mathtt {>}$	Integer	0
$\mathtt {<} \texttt {recombinendus1}\mathtt {>}$	Integer	$\mathtt {<} \texttt {recombinant}\mathtt {>}$
$\mathtt {<} \texttt {minimum\_distance}\mathtt {>}$	Real number	0.0
$\mathtt {<} \texttt {jet\_type\_selector}\mathtt {>}$	Real number	0.0
$\mathtt {<} \texttt {jet\_isolation\_distance}\mathtt {>}$	Real number	0.0
$\mathtt {<} \texttt {jet\_isolation\_parameter}\mathtt {>}$	Real number	0.0
$\mathtt {<} \texttt {jet\_isolation\_exponent}\mathtt {>}$	Real number	1.0

Since multiple steps of recombination are allowed, one must specify the level in the recombination chain on which a recombination step acts via the tag $\mathtt {<}$recombination_level_in$\mathtt {>}$. Recombination steps with the same $\mathtt {<}$recombination_level_in$\mathtt {>}$ values are run in parallel. The tags recombination_level_in> and $\mathtt {<}  \texttt {recombination\_level\_out}\mathtt {>}$ define the input and output level of the recombination step, respectively, and by default equal 0 (all particles) and 1. Internally, the clusters of level $\mathtt {<}  \texttt {recombination\_level\_in}\mathtt {>}$ are copied to clusters of level $\mathtt {<}  \texttt {recombination\_level\_out}\mathtt {>}$ (to allow for parallel recombination with different jet resolution parameters for instance), and thereafter the recombination step is performed on clusters of level $\mathtt {<}$recombination_level_out$\mathtt {>}$. Once all steps for an output level have been done, the resulting clusters are merged to a new list of clusters, which can serve as input for subsequent, not necessarily consecutive, levels. The merged clusters of the final level are the output of the recombination procedure.

The list $\mathtt {<}  \texttt {recombination\_jet\_types\_in}\mathtt {>}$ allows to restrict the jet types on which the recombination step acts. If this list is specified, all recombined objects get the jet type given by the corresponding list $\mathtt {<}$recombination_jet_types_out$\mathtt {>}$. If $\mathtt {<}  \texttt {recombination\_jet\_types\_in}\mathtt {>}$ is not specified or empty, all visible jet types, i.e. those within $\mathtt {<}  \texttt {rapidity\_maximum}\mathtt {>}$, and all neutrinos are used as input and, if not recombined, passed to the output. Note that each jet type that should appear in the final output of the recombination routine must also be present in the input at every recombination level. This requirement can be satisfied by defining a sequence of recombination steps without the $\mathtt {<}  \texttt {recombination\_jet\_types\_in}\mathtt {>}$ parameter, thereby propagating all jet types through all levels.

Each recombination step necessarily contains different recombination rules, defined either for jets of the same type by the tag  or for jets of different type by the tag . An arbitrary name can be set for both types via $\mathtt {<}  \texttt {name}\mathtt {>}$, which is only used for output purposes. The jet-resolution parameter is fixed by $\mathtt {<}  \texttt {minimum\_distance}\mathtt {>}$, corresponding to the rapidity–azimuthal-angle distance, and the jet type of the result of the clustering by $\mathtt {<}  \texttt {recombinant}\mathtt {>}$.

For same-type-jet recombination, the type of the jets to cluster is defined by $\mathtt {<}  \texttt {recombinendus1}\mathtt {>}$. If $\mathtt {<}  \texttt {recombinendus1}\mathtt {>}$ is not specified, it is assumed to be equal to $\mathtt {<}  \texttt {recombinant}\mathtt {>}$. In the example in Listing 19, the same-type recombination with the name bjet-bjet defines the clustering of b jets (jet type 2) with a jet resolution parameter R=0.5 into flavourless jets (jet type 1).

A different-type recombination requires the definition of two different types of jets to be recombined via $\mathtt {<}  \texttt {recombinendus1}\mathtt {>}$ and $\mathtt {<}  \texttt {recombinendus}\mathtt {>}$. While $\mathtt {<}  \texttt {recombinendus}\mathtt {>}$ needs to be specified, $\mathtt {<}  \texttt {recombinendus1}\mathtt {>}$ is assumed to be equal to $\mathtt {<}  \texttt {recombinant}\mathtt {>}$, if not given. In Listing 19, the different-type recombination with the name bjet-jet defines the clustering of b jets and jets (jet type 1) into b jets with a jet resolution parameter R=0.5.

**Figure:**
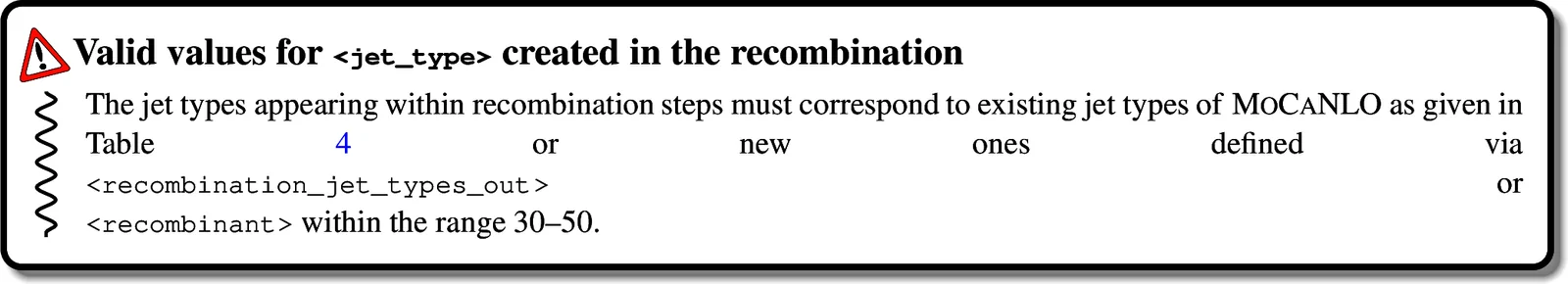

In the example Listing 20, in the first recombination step photons are clustered with antimuons, using the Cambridge–Aachen algorithm with jet resolution parameter R=0.1. This step passes all jets to level 1. At this level recombination steps 2 and 3 cluster jets of type 1 (and photons) twice in parallel using the anti-$$k_{\textrm{T}}$$ algorithm with resolution parameters 0.4 (slim-jets) and 0.8 (fat-jets). While the slim jets keep their jet types (if not clustered), the fat jets get jet type 31 via the parameter $$\mathtt {<}  \texttt {recombination\_jet\_types\_out}\mathtt {>}$$$${}=31$$ before clustering. Note that also photons (jet type 3) have to be present in the lists $$\mathtt {<}  \texttt {recombination\_jet\_types\_in}\mathtt {>}$$ and $$\mathtt {<}  \texttt {recombination\_jet\_types\_out}\mathtt {>}$$ to allow their clustering with fat jets. At level 2, which is the last one in the example, the outputs of recombination steps 2 and 3 are internally merged. Thus, the lists of jets in the output of the recombination involve the same objects twice. This has to be rectified by using appropriate cuts.

### Treatment of photons

The treatment of photons in the final state (via isolation, fragmentation, and conversion to jets) is determined to a large extent by the recombination rules.

#### Frixione isolation

Photons can be isolated via the Frixione isolation scheme [19]. To this end, a recombination rule as shown in Listing 21 should be added to the corresponding recombination step in the run card.

**Figure:**
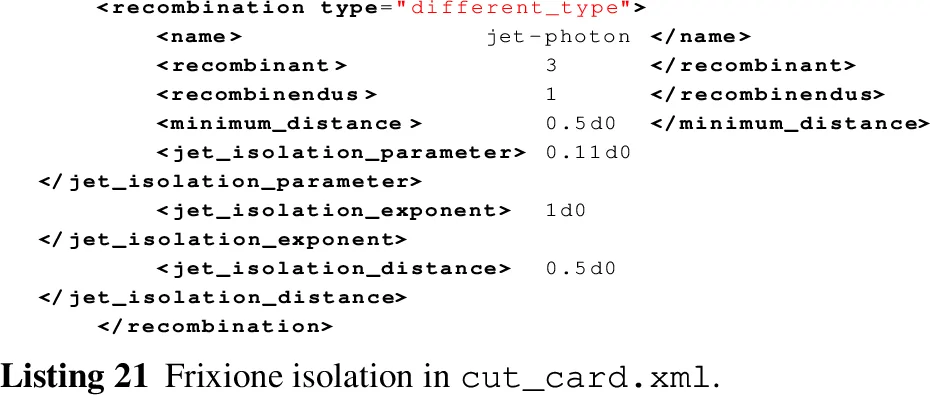

If the rapidity–azimuthal-angle distance ΔR between the photon (=$\mathtt {<}  \texttt {recombinant}\mathtt {>}$) and the jet (=$\mathtt {<}  \texttt {recombinendus}\mathtt {>}$) is smaller than the cone size $R_0$ (=$\mathtt {<}  \texttt {jet-isolation-distance}\mathtt {>}$), the two objects $\mathtt {<}  \texttt {recombinant}\mathtt {>}$ and $\mathtt {<}  \texttt {recombinendus}\mathtt {>}$ are discarded, unless they respect the relation

3 $$\begin{aligned} p_{{\textrm{T}},\textrm{recombinendus}} < \epsilon p_{{\textrm{T}},\gamma } \left( \frac{1-\cos (\Delta R_{\gamma ,\textrm{recombinendus}})}{1-\cos (R_0)}\right) ^n, \end{aligned}$$

where $$p_{{\textrm{T}},\textrm{recombinendus}}$$ and $$p_{{\textrm{T}},\gamma }$$ are the transverse momenta of the jet and the photon, respectively. If $$\Delta R < R_0$$ and Eq. (3) is fulfilled, the two objects are merged into one of type $$\mathtt {<}  \texttt {recombinant}\mathtt {>}$$, i.e. a photon. For $$\Delta R > R_0$$, both objects are kept independently of Eq. (3). The prefactor ϵ (=$$\mathtt {<}  \texttt {jet\_isolation\_parameter}\mathtt {>}$$) controls the allowed range of the energy fraction of the recombinendus in the cone around the photon and the exponent *n* (=$$\mathtt {<}  \texttt {jet\_isolation\_exponent}\mathtt {>}$$) the approach to the limit. Both should be positive parameters of order one and have default values $$\mathtt {<}  \texttt {jet\_isolation\_parameter}\mathtt {>}$$$${}=0$$ and $$\mathtt {<}  \texttt {jet\_isolation\_exponent}\mathtt {>}$$$${}=1$$.

**Figure:**
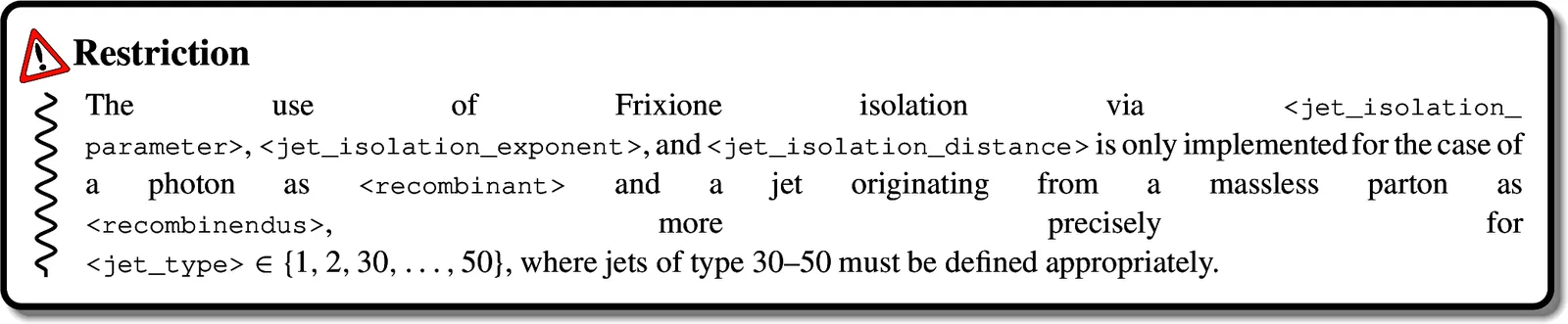

#### Quark-to-photon fragmentation function

Alternatively, isolated photons can be treated by employing a cut on the energy fraction of the photon within a recombined quark–photon pair. To this end, a recombination rule as shown in Listing 22 should be added to the corresponding recombination step in the run card.

**Figure:**
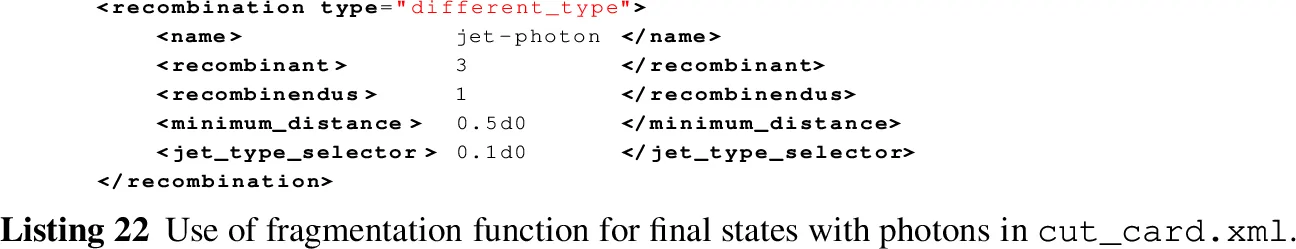

If $$\mathtt {<}  \texttt {jet\_type\_selector}\mathtt {>}$$$${}=0.0$$ (default), $$\mathtt {<}  \texttt {recombinant}\mathtt {>}$$ and $$\mathtt {<}  \texttt {recombinendus}\mathtt {>}$$ are recombined to $$\mathtt {<}  \texttt {recombinant}\mathtt {>}$$ as usual. For $$\mathtt {<}  \texttt {jet\_type\_selector}\mathtt {>}$$$${}>0.0$$, the recombination is modified as follows. If the energy fraction of the $$\mathtt {<}  \texttt {recombinendus}\mathtt {>}$$

$$ z = \frac{E_{\textrm{recombinendus}}}{E_{\textrm{recombinendus}}+E_{\textrm{recombinant}}} $$

is larger than $z_\textrm{cut}$ (=$\mathtt {<}  \texttt {jet\_type\_selector}\mathtt {>}$), both jets are recombined into a jet of type $\mathtt {<}  \texttt {recombinendus}\mathtt {>}$, otherwise they are recombined into a jet of type $\mathtt {<}  \texttt {recombinant}\mathtt {>}$.

A recombination depending on the energy fraction of the $\mathtt {<}  \texttt {recombinendus}\mathtt {>}$ must be accompanied by a fragmentation-function contribution in order to ensure IR-finite results. A quark-to-photon fragmentation function has been introduced in Refs. [20, 91] and the non-perturbative fit parameters entering it were obtained in Ref. [92] and read

4 $$\begin{aligned} \qquad \quad \mu _0 = 0.14 \,\text {GeV} \quad \text {and} \quad C = -\,13.26 . \end{aligned}$$

The quark-to-photon fragmentation function has been implemented in MoCaNLO following Refs. [21, 26, 93–95].

The quark-to-photon fragmentation function is needed in two different situations. The first case appears in the calculation of EW corrections (real photon radiation) to a LO process containing a final-state gluon. An example is the process $\text {p} \text {p} \rightarrow \text {Z} \text {j}$ [94], which contains the partonic process $\text {q} \bar{\text {q}} \rightarrow \text {Z} \text {g}$. When considering real photonic corrections, a hard photon can be recombined with a soft jet. Such a configuration leads to a QCD IR singularity that is not cancelled by any standard dipole but needs a quark-to-photon fragmentation function. The appropriate recombination rule for this case is given in Listing 23.

**Figure:**
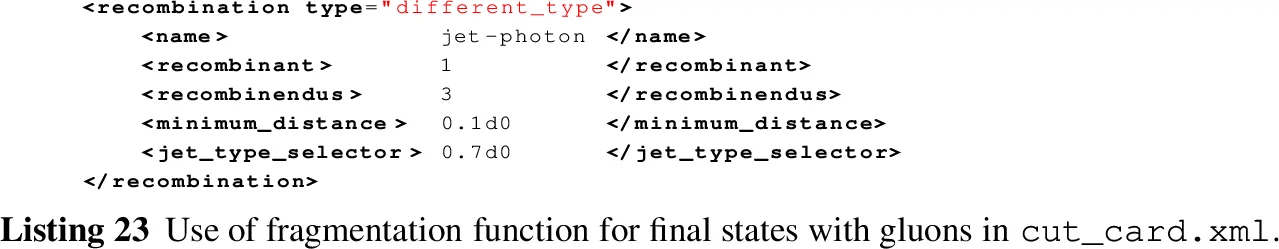

If the energy fraction of the photon ($\mathtt {<}  \texttt {recombinendus}\mathtt {>}$)

$$ z_\gamma = \frac{E_\textrm{recombinendus}}{E_\textrm{recombinendus}+E_\textrm{recombinant}} $$

is larger than $z_\textrm{cut}$ (=$\mathtt {<}  \texttt {jet\_type\_selector}\mathtt {>}$), the two objects are recombined into an object of type photon, otherwise they are recombined into a jet of type $\mathtt {<}  \texttt {recombinant}\mathtt {>}$. The real and integrated dipole-subtraction terms inherit the splitting cut $z_\textrm{cut}$ from the recombination rule (parameter $\mathtt {<}  \texttt {jet\_type\_selector}\mathtt {>}$) and the corresponding integrated dipoles, i.e. the ones with a fermionic emitter and a massless vector boson as emissus, take the quark-to-photon fragmentation function properly into account.

In the second case, QCD corrections are computed for a LO process containing a final-state photon. An example is the process $\text {p} \text {p} \rightarrow \text {Z} \gamma$ [96]. When considering QCD corrections, a soft photon can be recombined with a hard quark jet, for instance in the process $\text {u} \text {g} \rightarrow \text {Z} \gamma \text {u}$. This gives rise to a collinear singularity that is compensated by a contribution of the quark-to-photon fragmentation function in combination with the LO process $\text {u} \text {g} \rightarrow \text {Z} \text {u}$, as discussed in Refs. [22, 96]. The appropriate recombination rule for this case is given in Listing 22. If the energy fraction of the jet ($\mathtt {<}  \texttt {recombinendus}\mathtt {>}$)

$$ \qquad z_\text {j} = \frac{E_\textrm{recombinendus}}{E_\textrm{recombinendus}+E_\textrm{recombinant}} $$

is larger than $z_\textrm{cut}$ (=$\mathtt {<}  \texttt {jet\_type\_selector}\mathtt {>}$), the objects are recombined into a jet of the type $\mathtt {<}  \texttt {recombinendus}\mathtt {>}$, otherwise they are recombined into an object of type photon ($\mathtt {<}  \texttt {recombinant}\mathtt {>}$). As in the previous case, the real and integrated dipole-subtraction terms inherit the splitting cut from the recombination rule (parameter $\mathtt {<}  \texttt {jet\_type\_selector}\mathtt {>}$) and the integrated dipoles, i.e. the ones with a fermionic emitter and a massless vector boson as emissus, take the quark-to-photon fragmentation function properly into account.

**Figure:**
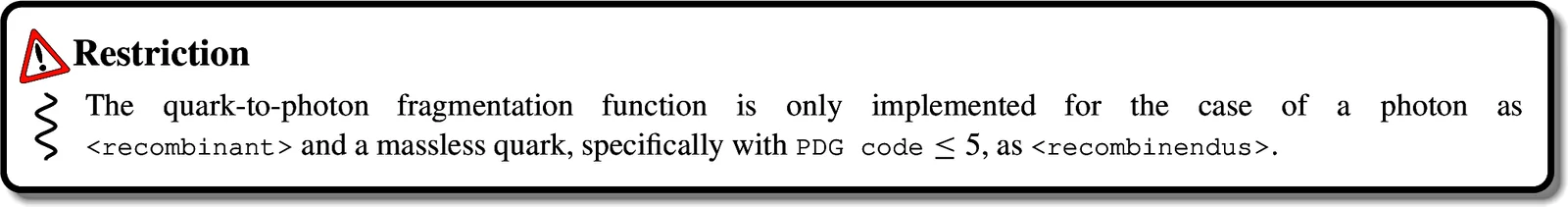

#### Photon-to-jet conversion function

The second type of IR singularities associated with photons concerns the splitting of a virtual photon into a quark–antiquark pair, $\gamma ^* \rightarrow \text {q} \bar{\text {q}}$, in the final state. These singularities are absorbed in MoCaNLO by using a photon-to-jet conversion function as discussed in detail in Refs. [23, 51]. In MoCaNLO the photon-to-jet conversion function is switched on by default in integrated dipoles for the splitting of a photon into a quark–antiquark pair. The implementation is based on the 5-flavour scheme, and the finite contributions are distributed according to their squared charges to the five light quarks. The value for $\Delta \alpha ^{(5)}_{\text {had}}(M_\text {Z} ^2)$ is taken from Ref. [97] and hard-coded:

$$ \qquad \Delta \alpha ^{(5)}_{\text {had}}(M_\text {Z} ^2) = 276.11\times 10^{-4}. $$

#### DIS factorisation scheme for photon PDFs

For photon PDFs in hadron collisions, the DIS factorisation scheme can also be used. It can be switched on adding the entry

**Figure:**
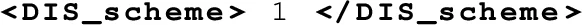

to the $$\mathtt {<}  \texttt {run\_parameters}\mathtt {>}$$ section of the param_card.xml. For the default value $$\mathtt {<}  \texttt {DIS\_scheme}\mathtt {>}$$$${}=0$$, the $$\overline{{\text {MS}}}$$ factorisation scheme is used, while $$\mathtt {<}  \texttt {DIS\_scheme}\mathtt {>}$$$${}=1$$ turns on the DIS factorisation scheme only for photon PDFs.

### Cuts

In MoCaNLO, the cuts are not only used for event selection, but they also serve to tag particles, order them, and create reconstructed objects. These cuts take the form of a list of operations to be applied sequentially to the final-state clusters resulting from the recombination. It is important to note that the order in which the cuts are applied is crucial.

The cuts operate on *sorted clusters*, which contain for each jet type a list of *recombinants*, resulting from the recombination. Recombinants, jets, and clusters are used as synonyms in the following, even if these can be any kind of clusters of particles or single particles like leptons. The recombinants are objects of a specific $\mathtt {<}  \texttt {jet\_type}\mathtt {>}$, as defined in column 5 of Table 4, or of new jet types within the range 30–50 generated by the recombination or cut procedure. Each jet in a list of recombinants has two additional attributes, a $\mathtt {<}  \texttt {jet\_tag}\mathtt {>}$ and a $\mathtt {<}  \texttt {jet\_order}\mathtt {>}$, which might be changed by applying a cut. By default, after recombination and before applying any cut, $\mathtt {<}  \texttt {jet\_tag}\mathtt {>}$ and $\mathtt {<}  \texttt {jet\_order}\mathtt {>}$ are equal to 0, and the lists of recombinants of each $\mathtt {<}  \texttt {jet\_type}\mathtt {>}$ are ordered according to their transverse momenta. The cuts do not change the entries in the list of recombinants, but only modify their associated parameters $\mathtt {<}  \texttt {jet\_tag}\mathtt {>}$ and $\mathtt {<}  \texttt {jet\_order}\mathtt {>}$. Besides the lists of recombinants, sorted clusters contain two further lists for each $\mathtt {<}  \texttt {jet\_type}\mathtt {>}$: the *ordered recombinants* and the *reconstructed recombinants*, which are both initially empty. The reconstructed recombinants contain the reconstructed objects created by the *reconstruction cuts*, and the lists of ordered recombinants are filled subsequently whenever a cut *orders* jets.

As mentioned above, the cuts allow to tag and order the jets, to apply cuts on jets with specific $\mathtt {<}  \texttt {jet\_tag}\mathtt {>}$ and $\mathtt {<}  \texttt {jet\_order}\mathtt {>}$, and to create new types of jets upon combining existing jets. Tagging a jet sets the corresponding $\mathtt {<}  \texttt {jet\_tag}\mathtt {>}$ to one. Ordering a jet adds this jet to the list of ordered recombinants and sets its $\mathtt {<}  \texttt {jet\_order}\mathtt {>}$ to a value equal to the position of the object in the list of ordered recombinants for the corresponding $\mathtt {<}  \texttt {jet\_type}\mathtt {>}$. The same jet is still present in the list of recombinants, but now with parameter $\mathtt {<}  \texttt {jet\_order}\mathtt {>}$ different from zero. Reconstruction cuts create new jets upon combining or cloning existing ones and add them to the lists of reconstructed recombinants. Upon ordering them, they also enter the corresponding lists of ordered recombinants.

Besides eliminating events and jets, the cuts define the jets that enter the histograms and the definition of dynamical scales. To this end, the usage of the lists of ordered recombinants is recommended, because only these are uniquely defined. Thus, all jets intended for histogram filling or scale definition should be tagged and ordered. Once a given type of clusters has been ordered, no further cuts must be applied that could remove or reorder any of the ordered jets of this type. Otherwise, subsequent access to these jets, for example by additional cuts, scale definitions, or histogram calls, may lead to run-time errors.

Even if a mechanism has been implemented to warn the user in case of inconsistencies in the input, not all problematic cases might be catched. In order to further check the correct implementation of the cuts in the cut_card.xml, it might be useful to compile the code with the flag DEBUG_CUTS switched on in include/debug_flags.h, to run it with a small number of events, e.g. using the tag $\mathtt {<}  \texttt {max\_generated\_events}\mathtt {>}$, and to check whether the cuts act as intended.

After this introduction, we turn to a more detailed description of the cut implementation. In a typical application, at a given stage of the cut sequence, tagged jets ($$\mathtt {<}  \texttt {jet\_tag}\mathtt {>}$$$${}=1$$), which are distinguished by their order, tagged jets without an order ($$\mathtt {<}  \texttt {jet\_order}\mathtt {>}$$$${}=0$$), and untagged jets ($$\mathtt {<}  \texttt {jet\_tag}\mathtt {>}$$$${}=0$$), which are unordered, are present simultaneously. In addition, there can be discarded jets with $$\mathtt {<}  \texttt {jet\_tag}\mathtt {>}$$$${}=-\,1$$. Some examples of cuts are shown in Listing 24 and explained in detail later in this section.

**Figure:**
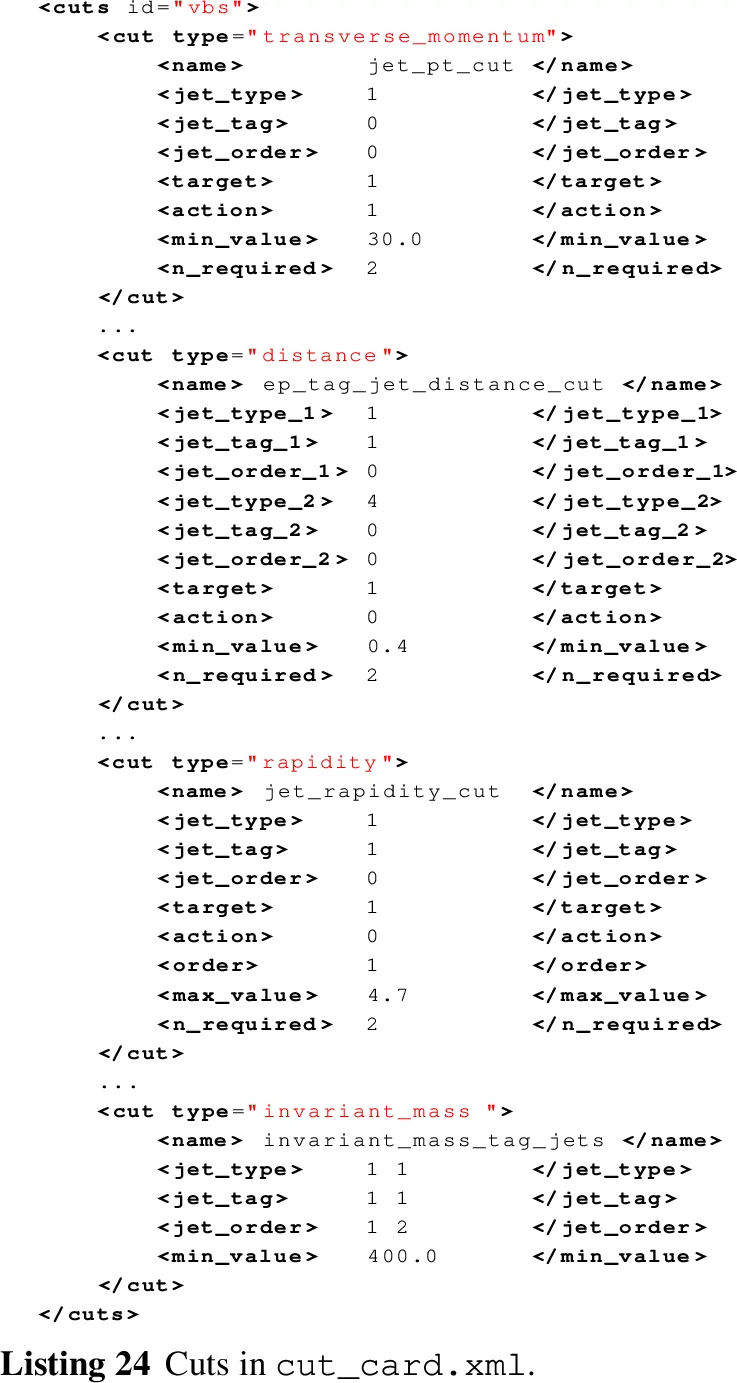

All cuts are defined in the section $\mathtt {<}  \texttt {cuts}\mathtt {>}$ in cut_card.xml. Each cut is set via the XML tag $\mathtt {<}  \texttt {cut}\mathtt {>}$ and can be given an arbitrary name with the tag $\mathtt {<}  \texttt {name}\mathtt {>}$, which is only used for output purposes. Cuts always come with a type, which is defined by the tag attribute type. There are four classes of cuts: single-particle, pair-particle, multi-particle, and reconstruction cuts. All these are explained in the following.

Each cut acts on a single jet or on a set of jets, characterised by their type, tag, and order status. If no matching jets are found, the cut either has no effect or results in the removal of the event, depending on its definition. The latter is to a large extent determined by three more tags: the $\mathtt {<}  \texttt {target}\mathtt {>}$, the $\mathtt {<}  \texttt {action}\mathtt {>}$, and the $\mathtt {<}  \texttt {order}\mathtt {>}$ of a cut.

The $$\mathtt {<}  \texttt {target}\mathtt {>}$$ tag specifies the object the cut acts on. It can take the values $$\mathtt {<}  \texttt {target}\mathtt {>}$$$${}=0$$, if acting on the whole event, $$\mathtt {<}  \texttt {target}\mathtt {>}$$$${}=1$$, if on the jet of $$\mathtt {<}  \texttt {jet\_type}\mathtt {>}$$ or $$\mathtt {<}  \texttt {jet\_type\_1}\mathtt {>}$$, or $$\mathtt {<}  \texttt {target}\mathtt {>}$$$${}=2$$, if on the jet of $$\mathtt {<}  \texttt {jet\_type\_2}\mathtt {>}$$ in a pair-particle cut, and even larger values in multi-particle or reconstruction cuts. By default, MoCaNLO sets $$\mathtt {<}  \texttt {target}\mathtt {>}$$$${}=0$$ for all cuts apart from the reconstruction cuts, where the default is $$\mathtt {<}  \texttt {target}\mathtt {>}$$$${}=1$$. In a reconstruction cut, for $$\mathtt {<}  \texttt {target}\mathtt {>}$$$${}=2$$ the tagging applies to both lists $$\mathtt {<}  \texttt {jet\_type\_1}\mathtt {>}$$ and $$\mathtt {<}  \texttt {jet\_type\_2}\mathtt {>}$$ (if present), while the cut acts always on the last object (most right) in $$\mathtt {<}  \texttt {jet\_type\_1}\mathtt {>}$$.

The tag $$\mathtt {<}  \texttt {action}\mathtt {>}$$ defines the action of the cut, with a default value $$\mathtt {<}  \texttt {action}\mathtt {>}$$$${}=-\,1$$. For $$\mathtt {<}  \texttt {action}\mathtt {>}$$$${}=-\,1$$ and $$\mathtt {<}  \texttt {target}\mathtt {>}$$$${}=0$$, the event is discarded, while for $$\mathtt {<}  \texttt {action}\mathtt {>}$$$${}=-\,1$$ but $$\mathtt {<}  \texttt {target}\mathtt {>}$$$${}>0$$, the corresponding target of the cut is eliminated, if the value of the corresponding observable is not between $$\mathtt {<}  \texttt {min\_value}\mathtt {>}$$ and $$\mathtt {<}  \texttt {max\_value}\mathtt {>}$$. For instance, if $$\mathtt {<}  \texttt {target}\mathtt {>}$$$${}=1$$, the jet of $$\mathtt {<}  \texttt {jet\_type}\mathtt {>}$$ or $$\mathtt {<}  \texttt {jet\_type\_1}\mathtt {>}$$ is removed, i.e. its parameter $$\mathtt {<}  \texttt {jet\_tag}\mathtt {>}$$ is set to -1, if the value of the corresponding observable is outside the cut range. For $$\mathtt {<}  \texttt {action}\mathtt {>}$$$${}=1$$ and $$\mathtt {<}  \texttt {target}\mathtt {>}$$$${}=0$$, the cut acts as a veto, i.e. if the variable is within the cut range the event is discarded. For $$\mathtt {<}  \texttt {action}\mathtt {>}$$$${}=1$$ and $$\mathtt {<}  \texttt {target}\mathtt {>}$$=1, the jet is tagged, i.e. its parameter $$\mathtt {<}  \texttt {jet\_tag}\mathtt {>}$$ is set to 1, if it passes the cut, i.e. if the value of the corresponding observable is in the range between $$\mathtt {<}  \texttt {min\_value}\mathtt {>}$$ and $$\mathtt {<}  \texttt {max\_value}\mathtt {>}$$. Conversely, for $$\mathtt {<}  \texttt {action}\mathtt {>}$$$${}=0$$ and $$\mathtt {<}  \texttt {target}\mathtt {>}$$$${}=1$$, the jet is untagged, i.e. its parameter $$\mathtt {<}  \texttt {jet\_tag}\mathtt {>}$$ is set to 0, if it does not pass the cut. The tagging and untagging acts on all jets with specified $$\mathtt {<}  \texttt {jet\_type}\mathtt {>}$$, $$\mathtt {<}  \texttt {jet\_tag}\mathtt {>}$$, and $$\mathtt {<}  \texttt {jet\_order}\mathtt {>}$$. For $$\mathtt {<}  \texttt {action}\mathtt {>}$$$${}=-1$$, all jets with specified $$\mathtt {<}  \texttt {jet\_type}\mathtt {>}$$ and $$\mathtt {<}  \texttt {jet\_order}\mathtt {>}$$, but with tag parameter greater or equal to $$\mathtt {<}  \texttt {jet\_tag}\mathtt {>}$$, are discarded.

Finally, cuts also come with an $$\mathtt {<}  \texttt {order}\mathtt {>}$$ tag, whose meaning depends on the cut type. All single-particle cuts with the tag $$\mathtt {<}  \texttt {order}\mathtt {>}$$$${}=1$$ and pair-particle cuts with the tag $$\mathtt {<}  \texttt {order\_1}\mathtt {>}$$$${}=1$$ or $$\mathtt {<}  \texttt {order\_2}\mathtt {>}$$$${}=1$$ order all respective tagged jets after application of the cut by setting their parameter $$\mathtt {<}  \texttt {jet\_order}\mathtt {>}$$ to consecutive values larger than zero. The default is $$\mathtt {<}  \texttt {order}\mathtt {>}$$$${}=0$$, which means do not order. Multi-particle cuts and reconstruction cuts with $$\mathtt {<}  \texttt {order}\mathtt {>}$$$${}=1$$ set the order of the tagged jets to the number of previously tagged jets plus one.

**Figure:**
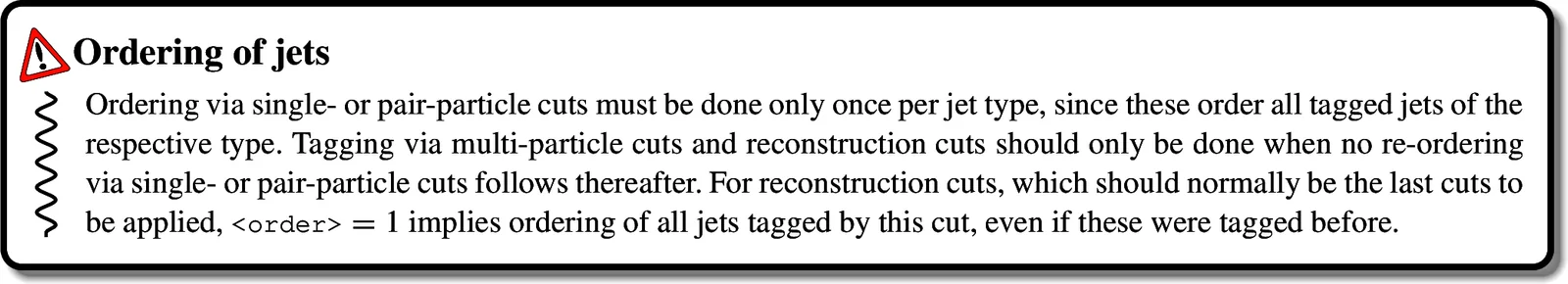

A **single-particle cut** is defined for the observable of a single particle, e.g. a cut on its transverse momentum. The corresponding parameters, possible values, and default values are summarised in Table 16. A single-particle cut requires the tag $\mathtt {<}  \texttt {jet\_type}\mathtt {>}$ specifying the single jet the cut is applied to, a minimum and/or a maximum value in $\mathtt {<}  \texttt {min\_value}\mathtt {>}$ and/or $\mathtt {<}  \texttt {max\_value}\mathtt {>}$, respectively, and the number of required particles $\mathtt {<}  \texttt {n\_required}\mathtt {>}$ of this jet type in the final state. Optionally, $\mathtt {<}  \texttt {jet\_tag}\mathtt {>}$ and $\mathtt {<}  \texttt {jet\_order}\mathtt {>}$ can be specified. Otherwise, these are assumed to be zero, meaning that the cut applies to all untagged and unordered jets. For a sensible cut, at least the tags $\mathtt {<}  \texttt {jet\_type}\mathtt {>}$ and either $\mathtt {<}  \texttt {min\_value}\mathtt {>}$ or $\mathtt {<}  \texttt {max\_value}\mathtt {>}$ have to be provided. The action of single-particle cuts is summarised in Table 17.

**Table 16:** Parameters and possible values of single-particle cuts. For a cut of type rapidity or pseudorapidity, the default value of $\mathtt {<}  \texttt {min\_value}\mathtt {>}$ is the negative of $\mathtt {<}  \texttt {max\_value}\mathtt {>}$

Parameter	Possible values	Default value
$\mathtt {<} \texttt {name}\mathtt {>}$	String	None
$\mathtt {<} \texttt {jet\_type}\mathtt {>}$	1,2,…,50	0
$\mathtt {<} \texttt {jet\_tag}\mathtt {>}$	0, 1	0
$\mathtt {<} \texttt {jet\_order}\mathtt {>}$	0,1,⋯	0
$\mathtt {<} \texttt {target}\mathtt {>}$	0, 1	0
$\mathtt {<} \texttt {action}\mathtt {>}$	-1,0,1	-1
$\mathtt {<} \texttt {order}\mathtt {>}$	0, 1	0
$\mathtt {<} \texttt {n\_required}\mathtt {>}$	0,1,…	0
$\mathtt {<} \texttt {min\_value}\mathtt {>}$	Real number	0.0
$\mathtt {<} \texttt {max\_value}\mathtt {>}$	Real number	Huge(0.0)

**Table 17:** Action of single-particle cuts

$\mathtt {<} \texttt {target}\mathtt {>}$	$\mathtt {<} \texttt {action}\mathtt {>}$	Result
0	-1	Remove event if less than $\mathtt {<} \texttt {n\_required}\mathtt {>}$ jets pass the cut
0	1	Remove event if the cut variable for at least one jet is within $\mathtt {<} \texttt {min\_value}\mathtt {>}$ and $\mathtt {<} \texttt {max\_value}\mathtt {>}$ (veto). (parameter $\mathtt {<} \texttt {n\_required}\mathtt {>}$ not allowed!)
1	1	Tag jet if it passes the cut and remove event if less than $\mathtt {<} \texttt {n\_required}\mathtt {>}$ tagged jets pass the cut
1	0	Untag jet if it does not pass the cut and remove event if less than $\mathtt {<} \texttt {n\_required}\mathtt {>}$ tagged jets pass the cut
1	-1	Remove jet if it does not pass the cut and remove event if less than $\mathtt {<} \texttt {n\_required}\mathtt {>}$ (tagged or untagged) jets pass the cut. Jets are removed irrespective of their tagging status

$\mathtt {<} \texttt {order}\mathtt {>}$	Result	
1	Order tagged jets according to $p_{\textrm{T}}$ and add them to the list of ordered recombinants	

Available single-particle cut types are:

- transverse_momentum,    $$p_{\textrm{T}}=\sqrt{p_x^2+p_y^2}$$,
- energy,    $$E=p_0$$,
- rapidity,    $$\displaystyle y = \frac{1}{2}\ln \left( \frac{p_0+p_z}{p_0-p_z}\right) $$,
- pseudorapidity,    $$\displaystyle \eta = \frac{1}{2}\ln \left( \frac{|\vec {p}|+p_z}{|\vec {p}|-p_z}\right) $$,
- theta_angle,    $$\displaystyle \frac{180}{\pi }\arccos \left( \frac{p_z}{|\vec {p}|}\right) $$,
- single_invariant_mass,    $$M=\sqrt{p^2}$$.

Note that the cut single_invariant_mass acts on the invariant mass of a single jet.

In the example in Listing 24, the first cut acts on untagged and unordered jets of $$\mathtt {<}  \texttt {jet\_type}\mathtt {>}$$$${}=1$$ and requires a transverse-momentum of $$p_{\text {T},\text {j}} >30\,\text {GeV} $$. It tags jets that fulfil this criterion and requires two tagged jets in the final state. The third cut in Listing 24 is a rapidity cut on tagged jets with $$|y_{\text {j}}|<4.7\,\text {GeV} $$, which untags jets and orders the remaining tagged jets.

A **pair-particle cut** is defined for an observable of two clusters, e.g. their $$\Delta R_{ij}$$ distance or their invariant mass $$m_{ij}$$. A pair-particle cut requires two jet types via $$\mathtt {<}  \texttt {jet\_type\_1}\mathtt {>}$$ and $$\mathtt {<}  \texttt {jet\_type\_2}\mathtt {>}$$, possibly being the same type, corresponding jet tags $$\mathtt {<}  \texttt {jet\_tag\_1}\mathtt {>}$$ and $$\mathtt {<}  \texttt {jet\_tag\_2}\mathtt {>}$$, and jet orders $$\mathtt {<}  \texttt {jet\_order\_1}\mathtt {>}$$ and $$\mathtt {<}  \texttt {jet\_order\_2}\mathtt {>}$$. Jet tags and orders that are not specified are assumed to be zero. The associated cut observable is evaluated for all cluster pairs with the two jet types, jet tags, and jet orders in the final state, excluding pairs of identical clusters. The complete set of parameters for pair-particle cuts is listed in Table 18, while the actions of the cuts are summarised in Table 19. The parameter $$\mathtt {<}  \texttt {n\_required}\mathtt {>}$$ is not allowed for $$\mathtt {<}  \texttt {target}\mathtt {>}$$$${}=0$$. For $$\mathtt {<}  \texttt {target}\mathtt {>}$$$${}=1$$ or $$\mathtt {<}  \texttt {target}\mathtt {>}$$$${}=2$$, $$\mathtt {<}  \texttt {n\_required}\mathtt {>}$$ applies to $$\mathtt {<}  \texttt {jet\_type\_1}\mathtt {>}$$ or $$\mathtt {<}  \texttt {jet\_type\_2}\mathtt {>}$$, respectively. Tagging and ordering are restricted to jets within $$\mathtt {<}  \texttt {jet\_type\_1}\mathtt {>}$$ for $$\mathtt {<}  \texttt {target}\mathtt {>}$$$${}=1$$ and to jets within $$\mathtt {<}  \texttt {jet\_type\_2}\mathtt {>}$$ for $$\mathtt {<}  \texttt {target}\mathtt {>}$$$${}=2$$.

**Table 18:** Parameters and possible values of pair-particle cuts

Parameter	Possible values	Default value
$\mathtt {<} \texttt {name}\mathtt {>}$	String	None
$\mathtt {<} \texttt {jet\_type\_1}\mathtt {>}$	1,2,…,50	0
$\mathtt {<} \texttt {jet\_tag\_1}\mathtt {>}$	0, 1	0
$\mathtt {<} \texttt {jet\_order\_1}\mathtt {>}$	0,1,⋯	0
$\mathtt {<} \texttt {jet\_type\_2}\mathtt {>}$	1,2,…,50	0
$\mathtt {<} \texttt {jet\_tag\_2}\mathtt {>}$	0, 1	0
$\mathtt {<} \texttt {jet\_order\_2}\mathtt {>}$	0,1,⋯	0
$\mathtt {<} \texttt {target}\mathtt {>}$	0, 1, 2	0
$\mathtt {<} \texttt {action}\mathtt {>}$	-1,0,1	-1
$\mathtt {<} \texttt {order\_1}\mathtt {>}$	0, 1	0
$\mathtt {<} \texttt {order\_2}\mathtt {>}$	0, 1	0
$\mathtt {<} \texttt {n\_required}\mathtt {>}$	0,1,…	0
$\mathtt {<} \texttt {min\_value}\mathtt {>}$	Real number	0.0
$\mathtt {<} \texttt {max\_value}\mathtt {>}$	Real number	Huge(0.0)

**Table 19:** Action of pair-particle cuts

$\mathtt {<} \texttt {target}\mathtt {>}$	$\mathtt {<} \texttt {action}\mathtt {>}$	Result
0	-1	Remove event if at least one specified jet pair does not pass the cut
0	1	Remove event if at least one specified jet pair passes the cut (veto)
1	1	Tag jet of type $\mathtt {<} \texttt {jet\_type\_1}\mathtt {>}$ if it passes the cut and remove event if less than $\mathtt {<} \texttt {n\_required}\mathtt {>}$ tagged jets of type $\mathtt {<} \texttt {jet\_type\_1}\mathtt {>}$ pass the cut
1	0	Untag jet of type $\mathtt {<} \texttt {jet\_type\_1}\mathtt {>}$ if it does not pass the cut and remove event if less than $\mathtt {<} \texttt {n\_required}\mathtt {>}$ tagged jets of type $\mathtt {<} \texttt {jet\_type\_1}\mathtt {>}$ pass the cut
1	-1	Remove jet of type $\mathtt {<} \texttt {jet\_type\_1}\mathtt {>}$ if it does not pass the cut and remove event if less than $\mathtt {<} \texttt {n\_required}\mathtt {>}$ (tagged or untagged) jets of type $\mathtt {<} \texttt {jet\_type\_1}\mathtt {>}$ pass the cut
2	1	Tag jet of type $\mathtt {<} \texttt {jet\_type\_2}\mathtt {>}$ if it passes the cut and remove event if less than $\mathtt {<} \texttt {n\_required}\mathtt {>}$ tagged jets of type $\mathtt {<} \texttt {jet\_type\_2}\mathtt {>}$ pass the cut
2	0	Untag jet of type $\mathtt {<} \texttt {jet\_type\_2}\mathtt {>}$ if it does not pass the cut and remove event if less than $\mathtt {<} \texttt {n\_required}\mathtt {>}$ tagged jets of type $\mathtt {<} \texttt {jet\_type\_2}\mathtt {>}$ pass the cut
2	-1	Remove jet of type $\mathtt {<} \texttt {jet\_type\_2}\mathtt {>}$ if it does not pass the cut and remove event if less than $\mathtt {<} \texttt {n\_required}\mathtt {>}$ (tagged or untagged) jets of type $\mathtt {<} \texttt {jet\_type\_2}\mathtt {>}$ pass the cut

$\mathtt {<} \texttt {target}\mathtt {>}$	$\mathtt {<} \texttt {order\_1}\mathtt {>}$	Result
1	1	Order tagged jets of type $\mathtt {<} \texttt {jet\_type\_1}\mathtt {>}$ according to $p_{\textrm{T}}$ and add them to the list of ordered recombinants

$\mathtt {<} \texttt {target}\mathtt {>}$	$\mathtt {<} \texttt {order\_2}\mathtt {>}$	Result
2	1	Order tagged jets of type $\mathtt {<} \texttt {jet\_type\_2}\mathtt {>}$ according to $p_{\textrm{T}}$ and add them to the list of ordered recombinants

Available pair-particle cut types are:

- distance,    $$\Delta R_{12}=\sqrt{(y_1-y_2)^2 + (\Delta \phi )^2}$$,    $$\Delta \phi = \min (|\phi _1-\phi _2|,2\pi -|\phi _1-\phi _2|)$$,
- pseudodistance,    $$\Delta r_{12}=\sqrt{(\eta _1-\eta _2)^2 + (\Delta \phi )^2}$$,
- pair_invariant_mass,    $$M_{12}=\sqrt{(p_1+p_2)^2}$$,
- pair_transverse_momentum,    $$p_{\textrm{T},12} =\sqrt{(p_{x,1}+p_{x,2})^2+(p_{y,1}+p_{y,2})^2}$$,
- angular_separation, $$\displaystyle \Delta \theta _{12}=\frac{180}{\pi }\arccos \left( \frac{\vec {p}_1\cdot \vec {p}_2}{|\vec {p}_1||\vec {p}_2|}\right) $$,
- rapidity_separation,    $$\Delta y_{12}=|y_1-y_2|$$,
- pseudorapidity_separation,    $$\Delta \eta _{12}=|\eta _1-\eta _2|$$,
- rapidity_product,    $$y_1\times y_2$$.

The second cut defined in Listing 24 is a distance cut between all positrons and tagged jets in the final state of ΔR>0.4, which is an isolation criterion so that jets too close to the lepton are removed from the list of jets.

A **multi-particle** cut involves an arbitrary number of particles. In contrast to the single-particle cuts, $$\mathtt {<}  \texttt {jet\_type}\mathtt {>}$$ and the optional parameters $$\mathtt {<}  \texttt {jet\_tag}\mathtt {>}$$ and $$\mathtt {<}  \texttt {jet\_order}\mathtt {>}$$ are lists of integers. If the latter two lists are not specified or shorter than the list $$\mathtt {<}  \texttt {jet\_type}\mathtt {>}$$, they are complemented with zero entries. The cut function depends on all specified jet entries, an example being the invariant mass of the sum of all momenta of the specified jets. The complete set of parameters for multi-particle cuts is listed in Table 20 and their actions in Table 21. For $$\mathtt {<}  \texttt {target}\mathtt {>}$$$${}=0$$, the action can be an ordinary cut if $$\mathtt {<}  \texttt {action}\mathtt {>}$$$${}=-1$$ or a veto cut if $$\mathtt {<}  \texttt {action}\mathtt {>}$$$${}=1$$. A $$\mathtt {<}  \texttt {target}\mathtt {>}$$$${}>0$$ is only allowed for specific cases mentioned below. The parameter $$\mathtt {<}  \texttt {n\_required}\mathtt {>}$$ is not supported for multi-particle cuts.

**Table 20:** Parameters and possible values of multi-particle cuts

Parameter	Possible values	Default value	Remark
$\mathtt {<} \texttt {name}\mathtt {>}$	String	None	
$\mathtt {<} \texttt {jet\_type}\mathtt {>}$	List of jet types	None	$n_{\textrm{jet}}$
$\mathtt {<} \texttt {jet\_tag}\mathtt {>}$	Corresponding list of jet tags	[0,...,0]	$\le n_{\textrm{jet}}$
$\mathtt {<} \texttt {jet\_order}\mathtt {>}$	Corresponding list of jet orders	[0,...,0]	$\le n_{\textrm{jet}}$
$\mathtt {<} \texttt {target}\mathtt {>}$	$0,1,\ldots ,n_{\textrm{jet}}$	0	>0 only for specific cuts
$\mathtt {<} \texttt {action}\mathtt {>}$	-1,1	-1	-1 only for $\mathtt {<} \texttt {target}\mathtt {>}$ = 0
$\mathtt {<} \texttt {order}\mathtt {>}$	0, 1	0	Only for specific cuts
$\mathtt {<} \texttt {min\_value}\mathtt {>}$	Real number	0.0	
$\mathtt {<} \texttt {max\_value}\mathtt {>}$	Real number	Huge(0.0)	
$\mathtt {<} \texttt {mid\_value}\mathtt {>}$	Real number	0.0	Only for specific cuts

**Table 21:** Action of multi-particle cuts

$\mathtt {<} \texttt {target}\mathtt {>}$	$\mathtt {<} \texttt {action}\mathtt {>}$	Result
0	-1	Remove event if cut not passed
0	1	Remove event if cut passed (veto)
n>0	1	Tag first *n* jets of list $\mathtt {<} \texttt {jet\_type}\mathtt {>}$ if the cut is passed and remove event if cut not passed

$\mathtt {<} \texttt {order}\mathtt {>}$	Result	
1	Set order of jets tagged by this cut and append them to the list of ordered recombinants (only for specific cuts)	

Available multi-particle cut types are:

- missing_transverse_momentum,    $p_{\text {T},\text {miss}} = \left| \sum _i \vec {p}_{\text {T},\nu _i}\right|$, (sum over all neutrinos),
- missing_energy,    $E_{\text {miss}} = \left| \sum _i E_{\nu _i}\right|$, (sum over all neutrinos),
- invariant_mass,    $M = \sqrt{(\sum _i p_i)^2}$, (sum over all specified jets),
- mt_jets_neutrinos,    $$M_\mathrm T =\sqrt{2p_{\text {T},\textrm{jets}} p_{\textrm{ T,miss}} [1 - \cos \Delta \phi (p_{\textrm{jets}},p_{\textrm{miss}})]}$$,
                            ($p_{\textrm{jets}}={}$momentum sum of all specified jets),
- zeppenfeld_variable,    $$\displaystyle z = \frac{2y_1 -y_2-y_3}{y_2-y_3}$$,
- jet_number,    $N_{\textrm{j}} = \sum _i 1$, (sum over all specified jets),
- max_invariant_mass,    $M_{\text {max}} = \max _{i_k}\sqrt{(\sum _k p_{i_k})^2}$,                           (maximum over all allowed jets $i_k$ for each specified type *k*),
- mid_invariant_mass,    $M = M_{\text {mid}}\pm \min _{i_k}| \sqrt{(\sum _k p_{i_k})^2}-M_{\text {mid}}|$, (minimum over all allowed jets $i_k$ for each specified type *k*),
- leading_transverse_momentum,    $p_{\text {T},\text {lead}} = \max _i \vec {p}_{\text {T},i}$, (maximum over all specified jets).

The first two cuts apply to the vector sum of the transverse momenta and energies of all neutrinos, respectively. No $\mathtt {<}  \texttt {jet\_type}\mathtt {>}$ input is needed, since all jets of type 6 and 21–26 are considered as neutrinos. The cut invariant_mass applies to the invariant mass of all jets of the list $\mathtt {<}  \texttt {jet\_type}\mathtt {>}$, and mt_jets_neutrinos to the transverse mass obtained from the sum of all neutrino momenta and the sum of the momenta of all specified jets. The latter quantity depends on the azimuthal angle $\Delta \phi$ between the sum of the jet momenta and the sum of the neutrino momenta. The cut zeppenfeld_variable acts on the Zeppenfeld variable (or centrality), defined via the rapidities of the three(!) listed jets, and the cut jet_number restricts the total number of jets of the specified types via the input values $\mathtt {<}  \texttt {min\_value}\mathtt {>}$ and $\mathtt {<}  \texttt {max\_value}\mathtt {>}$.

Among all multi-particle cuts, the ones discussed in this paragraph support tagging and ordering. The cut max_invariant_mass acts on the maximal invariant mass calculated from all possible jets of the specified types, e.g. for $$\mathtt {<}  \texttt {jet\_type}\mathtt {>}$$$${}= 1~5$$ on the largest invariant mass of all jet–electron pairs, while for $$\mathtt {<}  \texttt {jet\_type}\mathtt {>}$$$${}= 1~1~1$$ on the largest invariant mass of all combinations of three QCD jets. The cut mid_invariant_mass works analogously, but applies to the invariant mass closest to the value indicated in $$\mathtt {<}  \texttt {mid\_value}\mathtt {>}$$. These two cuts are particularly useful for tagging, as they determine the jets that form the maximal invariant mass or invariant mass closest to some given value. For $$\mathtt {<}  \texttt {target}\mathtt {>}$$$${}= n$$, they tag the first *n* jets of the list $$\mathtt {<}  \texttt {jet\_type}\mathtt {>}$$ that contribute to the invariant mass relevant for the cut. Note that *n* must be equal to or smaller than the number of specified jet types, which allows to tag all jets that form the invariant mass or only a subset. Only one jet per entry in $$\mathtt {<}  \texttt {jet\_type}\mathtt {>}$$ is tagged. Finally, for the cut leading_transverse_momentum, the entry with the leading transverse momentum is tagged, if *n* is greater than or equal to the position of the corresponding jet type in the list $$\mathtt {<}  \texttt {jet\_type}\mathtt {>}$$. The cut acts on the transverse momentum of the jet with the highest transverse momentum among the specified jet types. For $$\mathtt {<}  \texttt {target}\mathtt {>}$$$${}>0$$, only $$\mathtt {<}  \texttt {action}\mathtt {>}$$$${}=1$$ is allowed. For $$\mathtt {<}  \texttt {order}\mathtt {>}$$$${}=1$$, the jets tagged by this cut are appended to the existing list of ordered jets. This is also the case if the jets were already tagged but would be tagged by the multi-particle cut as well. If no jets fulfilling the tagging criterion are found, the event is cut.

A **reconstruction cut** serves to define reconstructed objects, e.g. reconstructed neutrinos or resonances, so that it does not necessarily act as a cut. If, however, values for $\mathtt {<}  \texttt {max\_value}\mathtt {>}$ and $\mathtt {<}  \texttt {min\_value}\mathtt {>}$ are provided, the corresponding cut acts on the first reconstructed object. The full set of parameters for a reconstruction cut is listed in Table 22.

**Table 22:** Parameters and possible values of reconstruction cuts

Parameter	Possible values	Default value	Remark
$\mathtt {<} \texttt {name}\mathtt {>}$	String	None	
$\mathtt {<} \texttt {jet\_type\_i}\mathtt {>}$	List of jet types	None	$n_i$
$\mathtt {<} \texttt {jet\_tag\_i}\mathtt {>}$	Corresponding list of jet tags	[0,...,0]	$\le n_i$
$\mathtt {<} \texttt {jet\_order\_i}\mathtt {>}$	Corresponding list of jet orders	[0,...,0]	$\le n_i$
$\mathtt {<} \texttt {parameter\_i}\mathtt {>}$	List of parameters for the cut	None	
$\mathtt {<} \texttt {reconstructed\_particle\_i}\mathtt {>}$	Reconstructed particle name	None	
$\mathtt {<} \texttt {target}\mathtt {>}$	$0,1,\ldots ,n_i-1$	1	
$\mathtt {<} \texttt {action}\mathtt {>}$	-1,0,1	0	
$\mathtt {<} \texttt {order}\mathtt {>}$	0, 1	0	
$\mathtt {<} \texttt {n\_required}\mathtt {>}$	0,1,…	1	
$\mathtt {<} \texttt {min\_value}\mathtt {>}$	Real number	0.0	
$\mathtt {<} \texttt {max\_value}\mathtt {>}$	Real number	Huge(0.0)	

A reconstruction cut involves an arbitrary number of particles, organised in (up to two) groups. As for the multi-particle cuts, $\mathtt {<}  \texttt {jet\_type\_i}\mathtt {>}$ and the optional parameters $\mathtt {<}  \texttt {jet\_tag\_i}\mathtt {>}$ and $\mathtt {<}  \texttt {jet\_order\_i}\mathtt {>}$, i=1,2, are lists of integers. If the latter two lists are not specified or shorter than the list $\mathtt {<}  \texttt {jet\_type\_i}\mathtt {>}$, they are complemented with zero entries. While the last (rightmost) entry in each list, denoted as $n_i$th entry in the following, defines the reconstructed object(s), the other entries specify the clusters used to define the reconstructed object(s) and are selected as for the multi-particle cuts above. More precisely, the last entry of $\mathtt {<}  \texttt {jet\_type\_i}\mathtt {>}$ defines the jet type of the reconstructed object, which can be either an existing type or a new type in the range 30–50, the last entry of $\mathtt {<}  \texttt {jet\_tag\_i}\mathtt {>}$ specifies whether the reconstructed object is tagged, and the last entry of $\mathtt {<}  \texttt {jet\_order\_i}\mathtt {>}$ determines whether the reconstructed tagged object is ordered and thus added to the list of ordered objects. The jet order of the reconstructed object is initially equal to zero. For cases where more than one object is reconstructed, a value of the last entry of $\mathtt {<}  \texttt {jet\_order\_i}\mathtt {>}$ larger than zero indicates how many of them should be ordered.

The properties of the reconstructed object can be defined in two alternative ways, either with the (real) values of $\mathtt {<}  \texttt {parameter\_i}\mathtt {>}$ or with the (string) values of $\mathtt {<}$reconstructed_particle_i$\mathtt {>}$. When indicating its name using $\mathtt {<}  \texttt {reconstructed\_particle\_i}\mathtt {>}$, the reconstructed object inherits the properties of an existing particle. Otherwise, its mass and width can be defined via the first two entries of the list $\mathtt {<}  \texttt {parameter\_i}\mathtt {>}$.

The cut parameters $\mathtt {<}  \texttt {action}\mathtt {>}$ and $\mathtt {<}  \texttt {order}\mathtt {>}$ work as for the multi-particle cuts, but apply only to the clusters used for reconstruction and not to the reconstructed ones. Note also that only jets that are tagged by reconstruction cuts are ordered by them. This is also the case if the jets were already tagged but would be tagged by the reconstruction cut as well. It is worth emphasising that, as for any other cut, the clusters used for reconstruction will either be tagged or untagged by a reconstruction cut (but not necessarily left in the same tagging state as before the cut). The action of the reconstruction cuts is summarised in Table 23. Depending on the cut, one or more reconstructed objects can be present. The event is discarded, if less than $\mathtt {<}  \texttt {n\_required}\mathtt {>}$ objects are reconstructed for the first group $\mathtt {<}  \texttt {jet\_type\_1}\mathtt {>}$, independently of the value of the cut observable, or if the latter fulfils the cut condition (see below). Default values for the cut parameters $\mathtt {<}  \texttt {target}\mathtt {>}$, $\mathtt {<}  \texttt {action}\mathtt {>}$, $\mathtt {<}  \texttt {order}\mathtt {>}$, and $\mathtt {<}  \texttt {n\_required}\mathtt {>}$ are 1, 0, 0, and 1, meaning that the clusters used for reconstruction are untagged and not ordered, and at least one recombined object must be present.

**Table 23:** Action of reconstruction cuts. Only the listed combinations of $\mathtt {<}  \texttt {target}\mathtt {>}$ and $\mathtt {<}  \texttt {action}\mathtt {>}$ are allowed

$\mathtt {<} \texttt {target}\mathtt {>}$	$\mathtt {<} \texttt {action}\mathtt {>}$	Result on event and all but last jets specified by
0	-1	Remove event if cut not passed, no tagging/ordering
0	1	Remove event if cut passed (veto), no tagging/ordering
n>0	1	Tag all but last jets of list(s) $\mathtt {<} \texttt {jet\_type\_i}\mathtt {>}$ with i≤n if the cut is passed and remove event if the cut is not passed
n>0	0	Untag all but last jets of list(s) $\mathtt {<} \texttt {jet\_type\_i}\mathtt {>}$ with i≤n if the cut is passed and remove event if the cut is not passed
$\mathtt {<} \texttt {order}\mathtt {>}$		Result on all but last jets specified by
1		Set order of jets tagged by this cut and append them to the list of ordered recombinants, if $$\mathtt {<} \texttt {target}\mathtt {>}$$$${}>0$$
$$\mathtt {<} \texttt {jet\_type\_i}\mathtt {>}$$$$_{\textrm{last}}$$		Result on last jet specified by , if $$\mathtt {<} \texttt {target}\mathtt {>}$$$${}>0$$
*n*		Set type of reconstructed object to *n* (use an existing particle type or a new one between 30 and 50)
$$\mathtt {<} \texttt {jet\_tag\_i}\mathtt {>}$$$$_{\textrm{last}}$$		Result on last jet specified by , if $$\mathtt {<} \texttt {target}\mathtt {>}$$$${}>0$$
1/0		Tag/do not tag reconstructed object
$$\mathtt {<} \texttt {jet\_order\_i}\mathtt {>}$$$$_{\textrm{last}}$$		Result on last jet specified by , if $$\mathtt {<} \texttt {target}\mathtt {>}$$$${}>0$$
1/0		Order/do not order reconstructed objects, i.e. add them to the list of tagged jets
n>0		Generate new list of *n* ordered objects,
		Only for cut transverse_momentum_ordering
$\mathtt {<} \texttt {n\_required}\mathtt {>}$		Result on last jet specified by
*n*		Require at least *n* reconstructed objects for the first group
		(not necessarily passing the cut condition)

**Figure:**
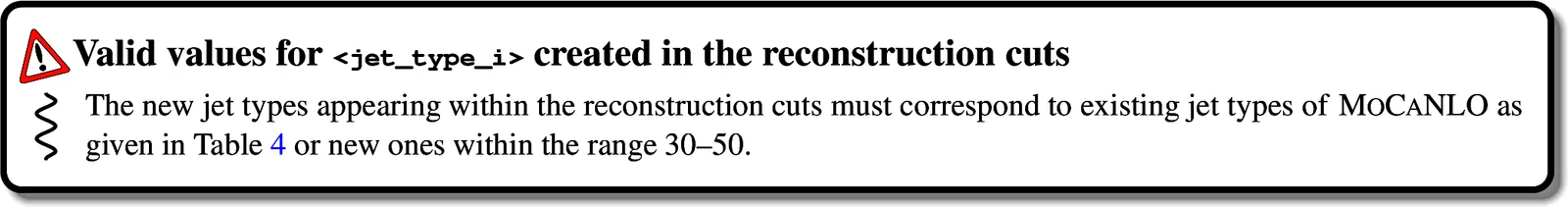

Available reconstruction cut types are:

- single_neutrino_pt_reconstruction,
- staggered_max_pt_tagging,
- two_resonances_likelihood_reconstruction,
- resonance_mass_reconstruction,    
- resonance_trivial_reconstruction,    
- cluster_cloning,    
- transverse_momentum_ordering.

Only the cut two_resonances_likelihood_reconstruction requires two lists of jet types, $\mathtt {<}  \texttt {jet\_type\_1}\mathtt {>}$ and $\mathtt {<}  \texttt {jet\_type\_2}\mathtt {>}$, while all other reconstruction cuts expect one list $\mathtt {<}  \texttt {jet\_type\_1}\mathtt {>}$ (together with corresponding list(s) for the tags $\mathtt {<}  \texttt {jet\_tag\_i}\mathtt {>}$, $\mathtt {<}  \texttt {jet\_order\_i}\mathtt {>}$, $\mathtt {<}  \texttt {parameter\_i}\mathtt {>}$, and $\mathtt {<}  \texttt {reconstructed\_particle\_i}\mathtt {>}$).

The cut single_neutrino_pt_reconstruction allows to reconstruct a massless object, more precisely its longitudinal momentum $p_1^z$, from the transverse component of the momentum $p_1$ of the first entry in list $\mathtt {<}  \texttt {jet\_type\_1}\mathtt {>}$ and the momenta $p_i$ of the $n_1-2$ subsequent entries of the same list. The equation

$$m_1^2 = \left( \sum _{i=1}^{n_1-1} p_i \right) ^2$$

is solved for the unknown $p_1^z$, where $m_1$ is the mass of the cluster specified via the first entry of $\mathtt {<}  \texttt {parameter\_1}\mathtt {>}$ or alternatively via $\mathtt {<}  \texttt {reconstructed\_particle\_1}\mathtt {>}$. The reconstructed object, with the reconstructed longitudinal momentum $p_1^z$, is put in the $n_1$th entry of $\mathtt {<}  \texttt {jet\_type\_1}\mathtt {>}$. If two solutions exist, reconstructed objects are generated for both, the first entry being the one with smaller longitudinal momentum. If the solutions are complex, the real part is selected. No cut is applied if at least one reconstructed neutrino is found.

The cut staggered_max_pt_tagging creates a new jet type with exactly one jet in its recombinant list from the list of all jet types specified by the first $n_1-1$ entries of $\mathtt {<}  \texttt {jet\_type\_1}\mathtt {>}$. The new object is the one with largest transverse momentum of the first type in $\mathtt {<}  \texttt {jet\_type\_1}\mathtt {>}$. If this list is empty, the one with largest transverse momentum of the second type in $\mathtt {<}  \texttt {jet\_type\_1}\mathtt {>}$ is searched. This is continued until one object is found or all $n_1-1$ types have been searched. The cut acts on the transverse momentum of the resulting object.

The cut two_resonances_likelihood_reconstruction reconstructs two resonances from the objects in the first $n_1-1$ and $n_2-1$ entries of the lists $\mathtt {<}  \texttt {jet\_type\_1}\mathtt {>}$ and $\mathtt {<}  \texttt {jet\_type\_2}\mathtt {>}$, respectively. From all allowed clusters with fitting tags and orders, those objects are selected that maximise the likelihood

$$ \mathcal {L}= \frac{1}{(M_1^2 - m_1^2)^2 + (m_1\Gamma _1)^2} \, \frac{1}{(M_2^2 - m_2^2)^2 + (m_2\Gamma _2)^2} , $$

where

$$ M_1^2=\left( \sum _{i_1=1}^{n_1-1} p_{i_1}\right) ^2, \qquad M_2^2=\left( \sum _{i_1=1}^{n_2-1} p_{i_2}\right) ^2, $$

and $$m_i$$ and $$\Gamma _i$$ are the masses and widths of the two resonances specified via the first two entries of $$\mathtt {<}  \texttt {parameter\_i}\mathtt {>}$$ or via $$\mathtt {<}  \texttt {reconstructed\_particle\_i}\mathtt {>}$$. For $$\mathtt {<}  \texttt {target}\mathtt {>}$$$${}=1$$, the tagging applies to all clusters that reconstruct the first resonance, for $$\mathtt {<}  \texttt {target}\mathtt {>}$$$${}=2$$, it applies to all clusters that reconstruct the two resonances. The tagging of the resonances is steered by the values of the last entries in $$\mathtt {<}  \texttt {jet\_tag\_i}\mathtt {>}$$. A possible cut acts on the invariant mass of the reconstructed resonance in the first list $$\mathtt {<}  \texttt {jet\_type\_1}\mathtt {>}$$.

The cuts resonance_mass2_reconstruction and resonance_mass_reconstruction reconstruct a resonance from the objects in the first $n_1-1$ entries of the list $\mathtt {<}  \texttt {jet\_type\_1}\mathtt {>}$. From all allowed clusters with fitting tags and orders, those objects are selected that minimise the distances

$$ \Delta = |M^2_1 - m^2_1| \quad \text { or }\quad \Delta = |M_1 - m_1|, $$

respectively, where

$$ M_1^2=\left( \sum _{i_1=1}^{n_1-1} p_{i_1}\right) ^2, $$

and $m_1$ is the mass of the resonance specified via the first entry of $\mathtt {<}  \texttt {parameter\_1}\mathtt {>}$ or via $\mathtt {<}  \texttt {reconstructed\_}\mathtt {>}$ $\mathtt {<}  \texttt {particle\_1}\mathtt {>}$. Tagging applies to all clusters that reconstruct the resonance. A possible cut acts on the invariant mass of the reconstructed resonance upon setting the $\mathtt {<}  \texttt {min\_value}\mathtt {>}$ and/or $\mathtt {<}  \texttt {max\_value}\mathtt {>}$.

The cut resonance_trivial_reconstruction reconstructs an object from those in the first $n_1-1$ entries of the list $\mathtt {<}  \texttt {jet\_type\_1}\mathtt {>}$. For each entry, the first allowed cluster with fitting tags and orders is taken. Tagging applies to all clusters that reconstruct the resonance. A possible cut acts on the invariant mass of the reconstructed resonance.

The cut cluster_cloning clones the objects in the first $n_1-1$ entries of the list $\mathtt {<}  \texttt {jet\_type\_1}\mathtt {>}$ into the new jet type. For each entry, all allowed clusters with fitting tags and orders are taken. No cut is applied. This cut does not change the tagging status of the input jets as opposed to the other reconstruction cuts. It can be used to combine objects with different jet types into one new jet type to be used in subsequent cuts, histograms or scales.

The cut transverse_momentum_ordering serves to order objects of different jet types according to their transverse momenta. Up to the number of objects specified by the $n_1$th entry of $\mathtt {<}  \texttt {jet\_order\_1}\mathtt {>}$ fitting to the first $n_1-1$ entries of $\mathtt {<}  \texttt {jet\_type\_1}\mathtt {>}$ are copied into a list for a new jet type (defined by this cut) and ordered by their transverse momenta. This can for instance be used to order leptons of different types (electrons, positrons, muons, ...) according to their transverse momenta. A possible cut acts on the transverse momentum of the selected object with largest transverse momentum. This cut does not change the tagging of the input jets.

Examples for the use of reconstruction cuts are provided in Listing 25.

**Figure:**
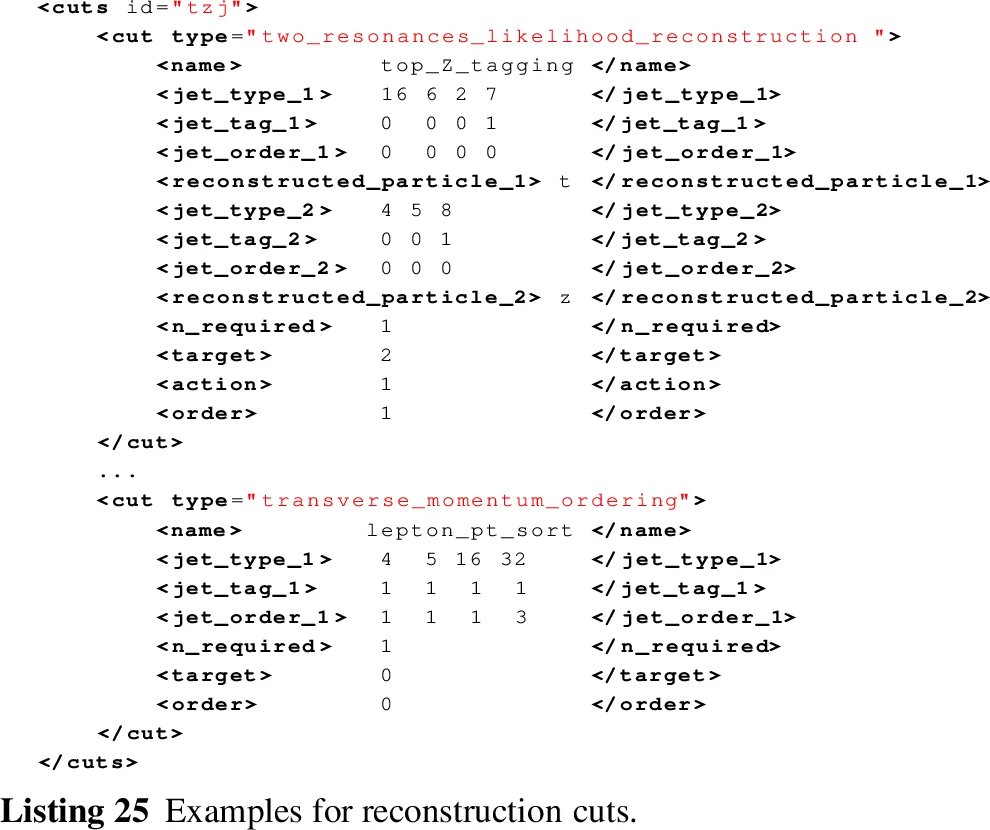

The first cut, named top_Z_tagging, reconstructs a top quark ($$\mathtt {<}  \texttt {jet\_type}\mathtt {>}$$$${}=7$$) from all untagged positive muons (16), neutrinos (6), and bottom jets (2), and a Z boson (8) from all untagged electrons (4) and positrons (5), and tags and orders all objects that reconstruct the top quark and the Z boson. The parameters for the masses and widths of the top quark and Z boson are taken from the values for the corresponding particles. The second cut, named lepton_pt_sort, sorts all tagged electrons, positrons and positively charged muons with $$\mathtt {<}  \texttt {jet\_order}\mathtt {>}$$ equal to 1 according to their transverse momenta and puts the first three objects into the $$\mathtt {<}  \texttt {jet\_type}\mathtt {>}$$ 32.

The typical use of the cut scheme for a VBS process with jet tagging can be found in Listing 24. The first cut of type transverse_momentum acts on all jets ($$\mathtt {<}  \texttt {jet\_type}\mathtt {>}$$$${}=1$$) and tags them if their transverse momentum is above $$30\,\text {GeV} $$. The following cut of type distance acts on tagged jets and untags them if their distance to positrons ($$\mathtt {<}  \texttt {jet\_type}\mathtt {>}$$$${}=4$$) is below 0.4. The third cut of type rapidity acts on tagged jets, untags them if their rapidity is outside the interval [-4.7,4.7], and orders the tagged jets. Finally, the cut of type invariant_mass acts on tagged jets with specified order, i.e. the two leading transverse-momentum jets, and discards events with $$M_{\text {j} _1\text {j} _2}<400\,\text {GeV} $$. Note that the order of the cuts is crucial.

### Fixing scales via the cut card

Dynamical scales can be defined via the cut card (cut_card.xml) by using generic scale types generated from selected final-state jets. To this end, the tag $\mathtt {<}  \texttt {dynamical\_scale\_type}\mathtt {>}$ (in the $\mathtt {<}  \texttt {run\_parameters}\mathtt {>}$ section of the param_card.xml) must be set to 1 and the definition of scales must be provided in the cut card. This requires a corresponding tag <scales id="..."> in run_card.xml. If this tag is not found, the code will stop with an error message.

The central scale can be constructed by multiplicatively combining several scale sums *l*, which in turn are sums of individual scales *k*. The implemented types for individual scales are listed in Table 24 and the corresponding parameters in Table 25.

**Table 24:** Implemented individual scale types. The final state jets *i* are selected via the input tags $\mathtt {<}  \texttt {jet\_type}\mathtt {>}$, $\mathtt {<}  \texttt {jet\_tag}\mathtt {>}$, and $\mathtt {<}  \texttt {jet\_order}\mathtt {>}$. The $\mathtt {<}  \texttt {power}\mathtt {>}$ of the scale is denoted my *m*

Observable	Type
$\left( \prod _{i=1}^n p_{\text {T},i}\right) ^{1/n}$	transverse_momentum_product
$(\sum _{i=1}^n p^m_{\text {T},i})^{1/m}$	transverse_momentum_sum
$\max _i p_{\text {T},i}$	transverse_momentum_maximum
$\left( \prod _{i=1}^n E_{\text {T},i}\right) ^{1/n}$	transverse_energy_product
$(\sum _{i=1}^n E^m_{\text {T},i})^{1/m}$	transverse_energy_sum
$\max _i E_{\text {T},i}$	transverse_energy_maximum
$\left( \prod _{i=1}^n M_{\text {T},i}\right) ^{1/n}$	transverse_mass_product
$(\sum _{i=1}^n M^m_{\text {T},i})^{1/m}$	transverse_mass_sum
$\max _i M_{\text {T},i}$	transverse_mass_maximum
$\left( \prod _{i=1}^n M_{i}\right) ^{1/n}$	invariant_mass_product
$\left( \sum _{i=1}^n M^m_{i}\right) ^{1/m}$	invariant_mass_sum
$\max _i M_{i}$	invariant_mass_maximum
$\left( \prod _{i=1}^n m_{i}\right) ^{1/n}$	particle_mass_product
$\left( \sum _{i=1}^n m^m_{i}\right) ^{1/m}$	particle_mass_sum

**Table 25:** Parameters of an individual scale type (some parameters are not available for all types)

Parameter	Possible values	Default value
$\mathtt {<} \texttt {name}\mathtt {>}$	String	None
$\mathtt {<} \texttt {jet\_type}\mathtt {>}$	List of jet types	None
$\mathtt {<} \texttt {jet\_tag}\mathtt {>}$	Corresponding list of jet tags	None
$\mathtt {<} \texttt {jet\_order}\mathtt {>}$	Corresponding list of jet orders	None
$\mathtt {<} \texttt {jet\_mass}\mathtt {>}$	Real number	0.0
$\mathtt {<} \texttt {jet\_particle}\mathtt {>}$	Particle name	None
$\mathtt {<} \texttt {offset}\mathtt {>}$	Real number	0.0
$\mathtt {<} \texttt {factor}\mathtt {>}$	Real number	1.0
$\mathtt {<} \texttt {power}\mathtt {>}$	Real number	1.0
$\mathtt {<} \texttt {weight}\mathtt {>}$	Real number	1.0

We define the transverse energy for a momentum *p* with transverse part $p_{\text {T}}$ via

5 $$\begin{aligned} \qquad \quad E_{\text {T}} = \sqrt{p^2+p_{\text {T}}^2} \end{aligned}$$

and the transverse mass via

6 $$\begin{aligned} \qquad \quad M_{\text {T}} = \sqrt{m^2+p_{\text {T}}^2} , \end{aligned}$$

where *m* is a mass associated to the corresponding jet (see below). The individual scales are calculated by taking the geometric average, the sum, or the maximum of the corresponding observable for a selection of final-state objects specified via the tags $$\mathtt {<}  \texttt {jet\_type}\mathtt {>}$$, $$\mathtt {<}  \texttt {jet\_tag}\mathtt {>}$$, and $$\mathtt {<}  \texttt {jet\_order}\mathtt {>}$$$${}>0$$. Note that the jets are selected according to the relevant tagging scheme as described at the end of Sect. 8.

The jets can be associated a mass indicated via the tag $\mathtt {<}  \texttt {jet\_mass}\mathtt {>}$ or the mass of the particle specified via the tag $\mathtt {<}  \texttt {jet\_particle}\mathtt {>}$, which can take the values in column 2 of Table 4. In addition, the resulting quantity can be increased by an offset $\mathtt {<}  \texttt {offset}\mathtt {>}$ (typically obtained from the masses of certain particles) and multiplied by a factor $\mathtt {<}  \texttt {factor}\mathtt {>}$. An individual scale of type *xxx*_product, where xxx refers to the observables in Table 24, is calculated via

$$ scale_k = \textit{factor}_k * \left( \prod _{i=1}^n (observable_{k,i})^{1/n} + \textit{offset}_k\right) $$

and an individual scale of type *xxx*_sum via

$$\begin{aligned} scale_k = \textit{factor}_k * \left[ \left( \sum _{i=1}^n (observable_{k,i})^{\textit{power}_k}\right) ^{1/{\textit{power}_k}} + \textit{offset}_k\right] , \end{aligned}$$

where *power* is fixed by the tag $\mathtt {<}  \texttt {power}\mathtt {>}$, and an individual scale of type *xxx*_max via

$$ scale_k = \textit{factor}_k * (\max _i observable_{k,i} + \textit{offset}_k). $$

Individual scales can be combined in a scale sum indicated by the tag $\mathtt {<}  \texttt {scale\_sum}\mathtt {>}$ as

$$ scale\_sum_l = \left[ \sum _k (scale_k)^{\textit{power}_l}\right] ^{1/{\textit{power}_l}}, $$

where $\mathtt {<}  \texttt {power}\mathtt {>}$ is the parameter of the scale sum *l* (see Table 26).

A scale sum can receive a further parameter $\mathtt {<}  \texttt {weight}\mathtt {>}$, which is relevant for the calculation of the final scale according to Eq. (7). A $\mathtt {<}  \texttt {scale}\mathtt {>}$ that is not part of a $\mathtt {<}  \texttt {scale\_sum}\mathtt {>}$ environment is considered as a scale sum with just this scale. The $\mathtt {<}  \texttt {weight}\mathtt {>}$ of this scale is passed to the corresponding $\mathtt {<}  \texttt {scale\_sum}\mathtt {>}$. The tag $\mathtt {<}  \texttt {weight}\mathtt {>}$ of the individual scale is ignored for a $\mathtt {<}  \texttt {scale}\mathtt {>}$ within a $\mathtt {<}  \texttt {scale\_sum}\mathtt {>}$.

The final scale is combined from all scale sums defined in the input card via

7 $$\begin{aligned} final\_scale = global\_factor * \left[ \prod _l scale\_sum_l^{weight_l}\right] ^{\frac{1}{\sum _l weight_l}}, \end{aligned}$$

where $global\_factor$ is the value of the tag $\mathtt {<}  \texttt {global\_factor}\mathtt {>}$ of the complete list of scales.

If $\mathtt {<}  \texttt {global\_factor}\mathtt {>}$ and the $\mathtt {<}  \texttt {weight}\mathtt {>}$ of all $\mathtt {<}  \texttt {scale\_sum}\mathtt {>}$ subsections contain two entries each, the first set of entries is used to construct the factorisation scale and the second one the renormalisation scale. Thus, $\mathtt {<}  \texttt {scale\_sum}\mathtt {>}$ sections with $\mathtt {<}  \texttt {weight}\mathtt {>}$ w1 0 $\mathtt {<}  \texttt {/weight}\mathtt {>}$ define the factorisation scale, while the ones with $\mathtt {<}  \texttt {weight}\mathtt {>}$ 0 w2 $\mathtt {<}  \texttt {/weight}\mathtt {>}$ define the renormalisation scale. Sections with $\mathtt {<}  \texttt {weight}\mathtt {>}$ w1 w2 $\mathtt {<}  \texttt {/weight}\mathtt {>}$ enter the definition of both scales.

**Table 26:** Parameters of a scale sum

Parameter	Possible values	Default value
$\mathtt {<} \texttt {power}\mathtt {>}$	Real number	1
$\mathtt {<} \texttt {weight}\mathtt {>}$	Real number	1

An example for the definition of a scale used for $\text {p} \text {p} \rightarrow \text {t} \bar{\text {t}}\text {b} \bar{\text {b}}\rightarrow \mu ^-\bar{\nu }_\mu \text {e} ^+\nu _\text {e} \bar{\text {b}}\text {b} \bar{\text {b}}\text {b}$ is given in Listing 26.

**Figure:**
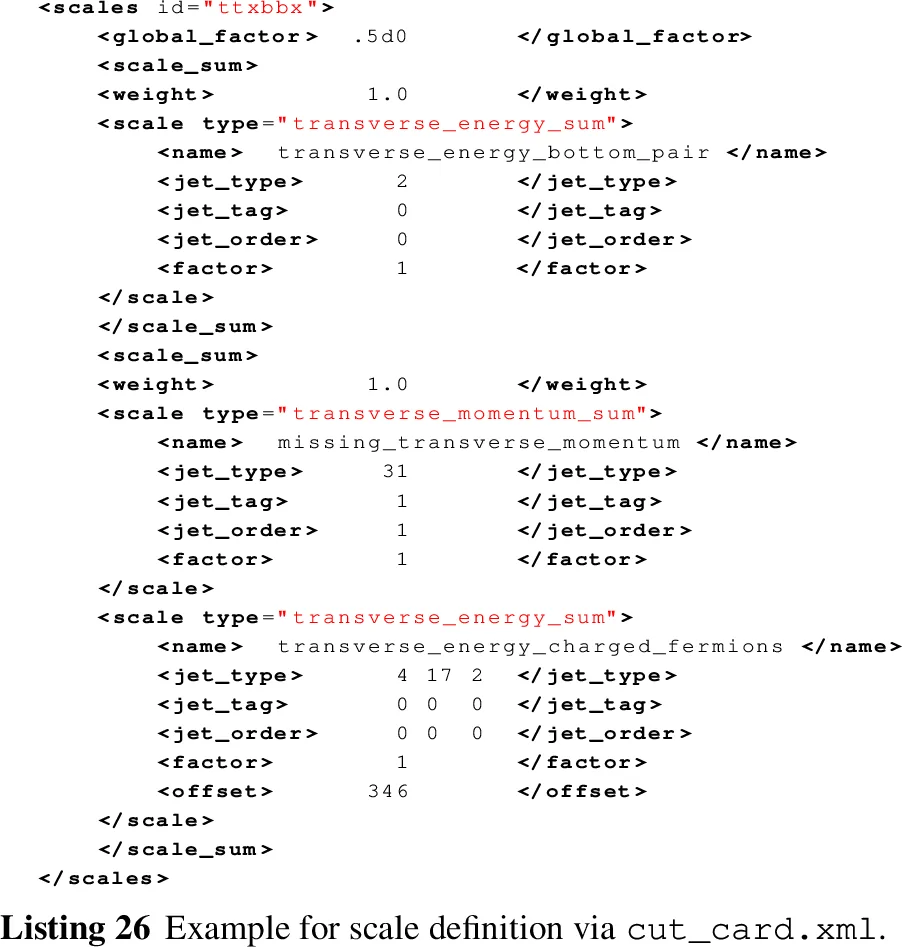

This defines the scale

8 $$\begin{aligned} \mu _0 = \frac{1}{2}\Biggl [ \Biggl ( \sum _{i={\textrm{j}_{\text {b}}}} E_{{\textrm{T}},i}\Biggr ) \Biggl ( p^{\textrm{miss}}_{{\textrm{T}}} + \sum _{i=\text {e} ^+,\mu ^-,{\textrm{j}_{\text {b}}}} E_{{\textrm{T}},i}+ 346\,\text {GeV} \Biggr )\Biggr ]^{1/2}, \end{aligned}$$

where $\textrm{j}_{\text {b}}$ denotes all bottom and antibottom jets. The missing transverse momentum $p^{\textrm{miss}}_{\textrm{T}}$ is defined via the reconstruction cut in Listing 27.

**Figure:**
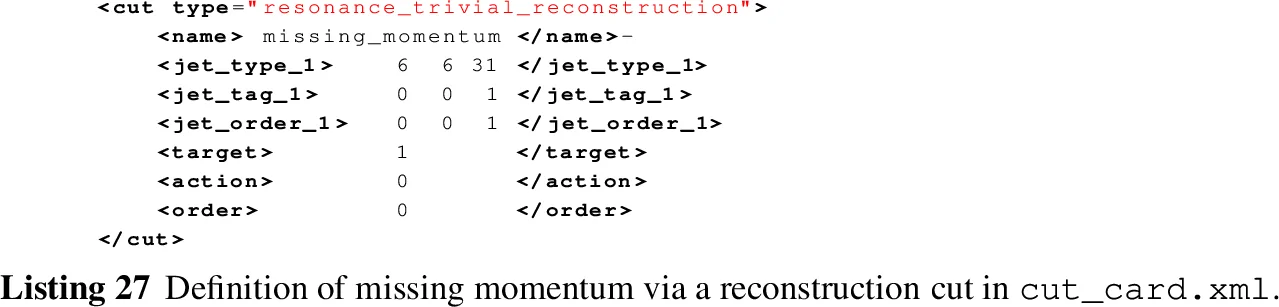

The top-quark mass is accounted for by the offset $2m_\textrm{t}=346\,\text {GeV}$. Alternatively, it can be taken into account by adding an extra scale as shown in Listing 28 to the second $\mathtt {<}  \texttt {scale\_sum}\mathtt {>}$ in Listing 26.

**Figure:**
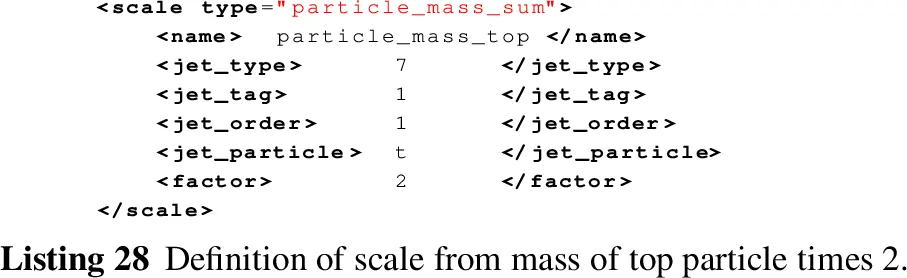

Another example used for $\text {p} \text {p} \rightarrow \text {e} ^+\text {e} ^-\gamma$ is shown in Listing 29

**Figure:**
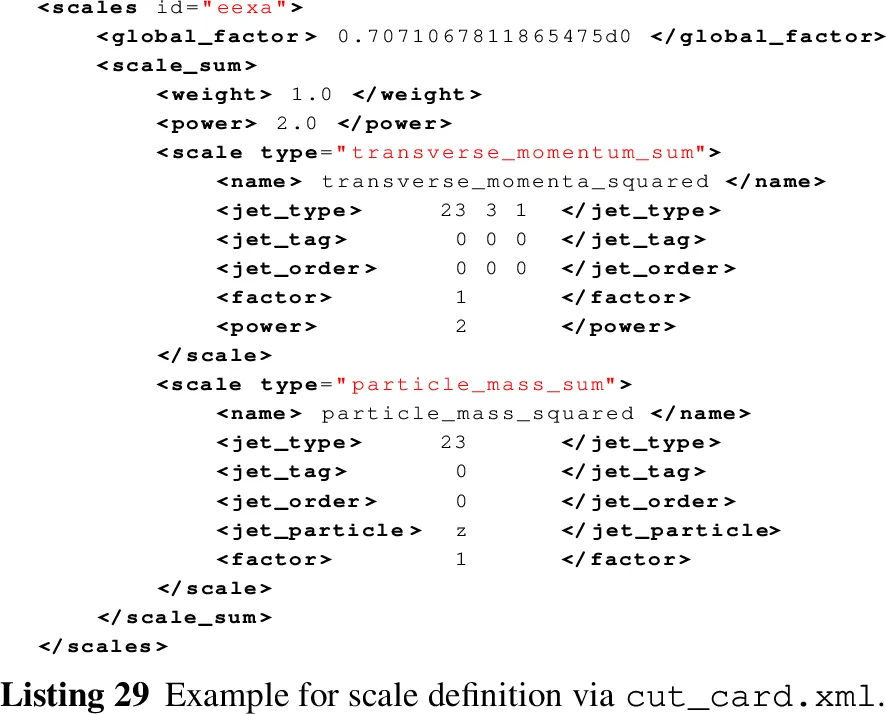

and defines the scale

9 $$\begin{aligned} \qquad \quad \mu _0 = \frac{1}{\sqrt{2}}\Biggl [ M_\text {Z} ^2+ \sum _{i=\text {Z},\gamma ,\text {j}} p_{{\textrm{T}},i}^2\Biggr ]^{1/2}. \end{aligned}$$

### Consistency checks of input

MoCaNLO performs some consistency checks on the input. When reading the cuts and scales, it checks, in particular, whether the input for $\mathtt {<}  \texttt {jet\_type}\mathtt {>}$, $\mathtt {<}  \texttt {jet\_tag}\mathtt {>}$, and $\mathtt {<}  \texttt {jet\_order}\mathtt {>}$ matches with the final state of the considered process or the objects constructed during the recombination and cut procedure. By default, the code stops if it detects a potential inconsistency, which is useful to correctly set up the input cards. At their own risk, the stop can be switched off for some warnings by the user by adding

**Figure:**
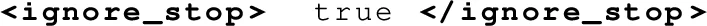

in the $\mathtt {<}  \texttt {cut}\mathtt {>}$ or $\mathtt {<}  \texttt {scale}\mathtt {>}$ environment. Moreover, by using the tag

**Figure:**
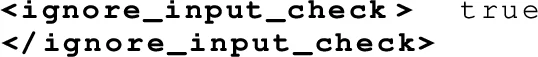

all checks for a particular $\mathtt {<}  \texttt {cut}\mathtt {>}$ or $\mathtt {<}  \texttt {scale}\mathtt {>}$ can be disabled. However, this is not recommended, as it usually leads to run-time errors.

## Listing observables with the plot_card.xml

The plot card plot_card.xml contains the section $\mathtt {<}  \texttt {histograms}\mathtt {>}$, where histograms of observables are listed. Individual histograms are defined in $\mathtt {<}  \texttt {histogram}\mathtt {>}$ sections, with their type specified by the attribute type. The histograms operate on momenta after cuts, i.e. on recombined and/or reconstructed objects called generically jets.

The implemented histogram types are listed in Table 27.

**Table 27:** Implemented histogram types

Category	Observable	Type
Single-jet based	$\sum _n E_n$	energy
Single-jet based	$(\sum _n p_n)_{\textrm{T}}$	transverse_momentum
Single-jet based	$\sum _n|p_{{\textrm{T}}_n}|$	transverse_momentum_scalar_sum
Single-jet based	$\sum _nE_{{\textrm{T}}n}$	transverse_energy
Single-jet based	$\sum _nM_{{\textrm{T}}n}$	transverse_mass
Single-jet based	$\sqrt{ (\sum _n p_n)^2}$	invariant_mass
Single-jet based	$y(\sum _np_n)$	rapidity
Single-jet based	$\eta (\sum _np_n)$	pseudorapidity
Single-jet based	$\cos \theta (\sum _np_n)$	cosine_angle
Single-jet based	$\tilde{M}_{{\textrm{T}}}$	transverse_mass_neutrino
Multi-jet based	$\Delta R_{ij}$	distance
Multi-jet based	$\Delta r_{ij}$	pseudodistance
Multi-jet based	$\phi _{ij}$	azimuthal_angle_separation
Multi-jet based	$\cos \theta _{ij,k}$	cosine_angle_separation
Multi-jet based	$y_{ij}$	rapidity_separation
Multi-jet based	$\eta _{ij}$	pseudorapidity_separation
Multi-jet based	$p_{{\textrm{T}},j}/p_{{\textrm{T}},i}$	transverse_momentum_ratio
Multi-jet based	$\cos \theta ^*_{ij,k}$	cosine_decay_angle
Multi-jet based	$\phi ^*_{ijkl,m}$	azimuthal_angle_planes
Multi-jet based	$z_{ijk}$	z3rap
Overall	$H_\text {T}$	total_transverse_energy
Overall	$\sqrt{\hat{s}}$	cms_energy

The parameters of a histogram are summarised in Table 28.

**Table 28:** Parameters of a histogram

Parameter	Possible values	Default value
$\mathtt {<} \texttt {name}\mathtt {>}$	String	None
$\mathtt {<} \texttt {jet\_type}\mathtt {>}$	List of jet types	None
$\mathtt {<} \texttt {jet\_tag}\mathtt {>}$	Corresponding list of jet tags	[0,...,0]
$\mathtt {<} \texttt {jet\_order}\mathtt {>}$	Corresponding list of jet orders	[0,...,0]
$\mathtt {<} \texttt {x\_min}\mathtt {>}$	Real number	Histogram-specific
$\mathtt {<} \texttt {x\_max}\mathtt {>}$	Real number	Histogram-specific
$\mathtt {<} \texttt {bin\_width}\mathtt {>}$	Real number	Histogram-specific
$\mathtt {<} \texttt {x\_scale\_up}\mathtt {>}$	Real number	1.0
$\mathtt {<} \texttt {x\_scale\_down}\mathtt {>}$	Real number	1.0
$\mathtt {<} \texttt {abs}\mathtt {>}$	True/false	False
$\mathtt {<} \texttt {use\_tagging}\mathtt {>}$	0, 1, 2	1
$\mathtt {<} \texttt {latex\_title}\mathtt {>}$	String	None
$\mathtt {<} \texttt {latex\_observable}\mathtt {>}$	Latex string	None
$\mathtt {<} \texttt {plot\_xmax}\mathtt {>}$	Real number	$\mathtt {<} \texttt {x\_max}\mathtt {>}$
$\mathtt {<} \texttt {plot\_xmin}\mathtt {>}$	Real number	$\mathtt {<} \texttt {x\_min}\mathtt {>}$
$\mathtt {<} \texttt {plot\_ymax}\mathtt {>}$	Real number	None
$\mathtt {<} \texttt {plot\_ymin}\mathtt {>}$	Real number	None
$\mathtt {<} \texttt {plot\_kmax}\mathtt {>}$	Real number	None
$\mathtt {<} \texttt {plot\_kmin}\mathtt {>}$	Real number	None
$\mathtt {<} \texttt {logscale}\mathtt {>}$	True/false	Histogram-specific

Listing 30 shows as an example the definition of a histogram of the transverse momentum of a bottom-jet pair. The parameter $\mathtt {<}  \texttt {name}\mathtt {>}$ is used for naming the resulting data file, e.g. for the example in Listing 30 it reads histogram_transverse_momentum_bb12.dat. The tags $\mathtt {<}  \texttt {jet\_type}\mathtt {>}$, $\mathtt {<}  \texttt {jet\_tag}\mathtt {>}$, and $\mathtt {<}  \texttt {jet\_order}\mathtt {>}$ indicate which transverse momentum to plot. The arrays of integers in $\mathtt {<}  \texttt {jet\_type}\mathtt {>}$ specify the types of jets, those in $\mathtt {<}  \texttt {jet\_tag}\mathtt {>}$ their tagging status and those in $\mathtt {<}  \texttt {jet\_order}\mathtt {>}$ the position of the jets in the ordered lists for $\mathtt {<}  \texttt {jet\_type}\mathtt {>}$ as defined by the tagging scheme (see below). While $\mathtt {<}  \texttt {jet\_type}\mathtt {>}$ and $\mathtt {<}  \texttt {jet\_order}\mathtt {>}$ are mandatory for all jet-based histograms, $\mathtt {<}  \texttt {jet\_tag}\mathtt {>}$ is assumed to be zero if not present. The definitions in Listing 30 correspond to add the momenta of the first and second tagged and ordered b jets and to bin the transverse momentum of this sum, labelled as $p_{\text {T},\text {b} _1\text {b} _2}$. The tags $\mathtt {<}  \texttt {x\_min}\mathtt {>}$ and $\mathtt {<}  \texttt {x\_max}\mathtt {>}$ fix the observable’s range to bin and $\mathtt {<}  \texttt {bin\_width}\mathtt {>}$ the bin width to be used.

**Figure:**
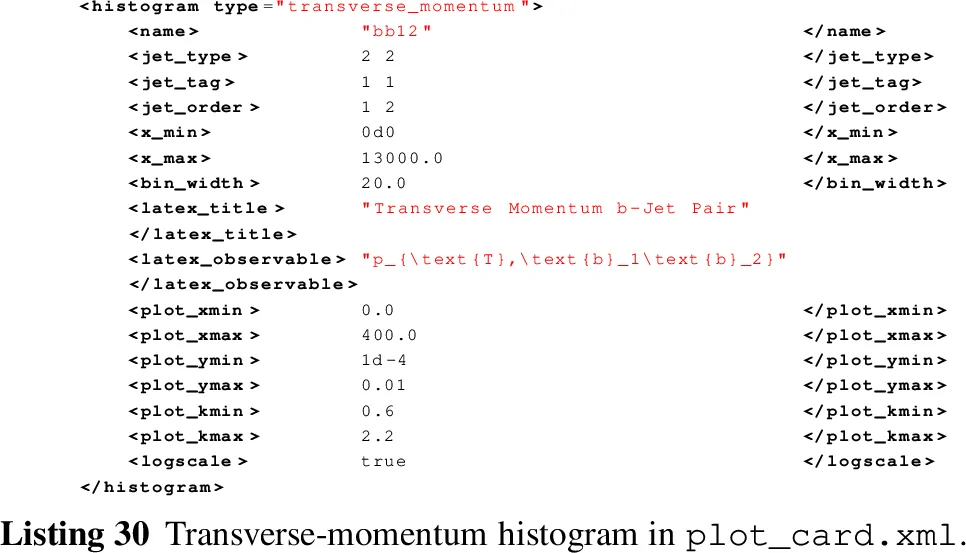

The tags $\mathtt {<}  \texttt {x\_scale\_up}\mathtt {>}$, $\mathtt {<}  \texttt {x\_scale\_down}\mathtt {>}$, and $\mathtt {<}  \texttt {abs}\mathtt {>}$ are optional and modify the default settings of the histogram. If $\mathtt {<}  \texttt {abs}\mathtt {>}$ is set to true, the absolute value of the observable is binned. With $\mathtt {<}  \texttt {x\_scale\_up}\mathtt {>}$ and $\mathtt {<}  \texttt {x\_scale\_down}\mathtt {>}$, the observable is multiplied and divided by the respective input number before filling the bins. By default, observables with mass dimension are measured in GeV and angles in degrees. In the latter case, setting $\mathtt {<}  \texttt {x\_scale\_down}\mathtt {>}$ to 180 and $\mathtt {<}  \texttt {x\_scale\_up}\mathtt {>}$ to 3.1415 will display the distributions in radians.

The remaining tags are only passed through the histogram files to gnuplot. The strings given in $\mathtt {<}  \texttt {latex\_title}\mathtt {>}$ and $\mathtt {<}  \texttt {latex\_observable}\mathtt {>}$ are used as title and observable label of the histogram in the LaTeX output. The other tags define the visible plot ranges for the *x* axis via $\mathtt {<}  \texttt {plot\_xmin}\mathtt {>}$and $\mathtt {<}  \texttt {plot\_xmax}\mathtt {>}$, the ranges of the *y* axis via $\mathtt {<}  \texttt {plot\_ymin}\mathtt {>}$ and $\mathtt {<}  \texttt {plot\_ymax}\mathtt {>}$, while $\mathtt {<}  \texttt {plot\_kmin}\mathtt {>}$ and $\mathtt {<}  \texttt {plot\_kmax}\mathtt {>}$ define the *y* axis range of the *k* factor in relative plots. The tag $\mathtt {<}  \texttt {logscale}\mathtt {>}$ allows to specify whether to use a logarithmic scale in the plot.

For the single-jet-based histogram types, the lists $\mathtt {<}  \texttt {jet\_type}\mathtt {>}$, $\mathtt {<}  \texttt {jet\_tag}\mathtt {>}$, and $\mathtt {<}  \texttt {jet\_order}\mathtt {>}$ can have an arbitrary number of entries (same number for all lists) and the observables of all partons that match the corresponding entries are summed up as indicated in Table 27. The transverse energy $E_{\textrm{T}}$ and the transverse mass $M_{\textrm{T}}$ are defined as in Eqs. (5) and (6), respectively. In the histogram of type transverse_mass_neutrino, the momenta of jets of type neutrino are summed separately from all other entries, resulting in two momenta $p_\nu$ and $p_{\textrm{vis}}$. From these momenta the transverse mass is calculated as

$$ \tilde{M}_{{\textrm{T}}} = \sqrt{(p_{{\textrm{T}},\textrm{vis}}+p_{{\textrm{T}},\nu })^2 - (\vec {p}_{{\textrm{T}},\textrm{vis}}+\vec {p}_{{\textrm{T}},\nu })^2}. $$

For multi-jet-based histogram types with two indices *ij*, exactly two entries are expected in each of these lists. In those cases all observables are computed from momenta in the laboratory system. For the observables with indices *ij*, *k* either two or three entries can be given. If the third entry is missing for cosine_angle_separation, $\cos \theta _{ij}$ is calculated in the laboratory system. If the third entry is missing for cosine_decay_angle, $\cos \theta ^*_{ij}$, i.e. the decay angle of parton *i* in the rest frame of parton *j*, is computed by first boosting the momentum *i* in the frame of *j*, and evaluating in this frame the angle of *i* with respect to the boost direction. If the third argument *k* is present, in both cases all momenta are first boosted to the rest frame of parton *k*, and thereafter the angles are calculated as specified above. The histogram azimuthal_angle_planes works similarly and produces a distribution in the angle between two planes, defined by the momenta of the first two pairs of the specified jets. Without a fifth jet specified, this angle is calculated in the laboratory frame, otherwise it is calculated in the rest system of the fifth jet. Finally, the Zeppenfeld variable (or centrality) is defined as

$$ z_{ijk} = \frac{2 y_i - y_j - y_k}{2(y_j-y_k)} $$

and requires exactly three entries.

For the histograms total_transverse_energy and cms_energy the tags $\mathtt {<}  \texttt {jet\_type}\mathtt {>}$, $\mathtt {<}  \texttt {jet\_tag}\mathtt {>}$, and $\mathtt {<}  \texttt {jet\_order}\mathtt {>}$ are not allowed. The former histogram yields the sum of the transverse energies of all final-state objects, including neutrinos (missing energy) and bremsstrahlung objects. The histogram cms_energy yields the total partonic CM energy.

Histograms can be based on three different tagging schemes, which are selected using the tag $$\mathtt {<}  \texttt {use\_tagging}\mathtt {>}$$, which either applies to all histograms (when specified in the section $$\mathtt {<}  \texttt {histograms}\mathtt {>}$$) or to individual histograms (when specified within the $$\mathtt {<}  \texttt {histogram}\mathtt {>}$$ definitions). The default is $$\mathtt {<}  \texttt {use\_tagging}\mathtt {>}$$$${}=1$$.

For $$\mathtt {<}  \texttt {use\_tagging}\mathtt {>}$$$${}=0$$, tagging is not used, i.e. $$\mathtt {<}  \texttt {jet\_tag}\mathtt {>}$$ should not be present. All jets are taken from the lists of *recombinants* of the corresponding $$\mathtt {<}  \texttt {jet\_type}\mathtt {>}$$, and $$\mathtt {<}  \texttt {jet\_order}\mathtt {>}$$ refers to the corresponding position in this list. The ordering in the list of *recombinants* corresponds to an ordering in transverse momenta.

**Figure:**
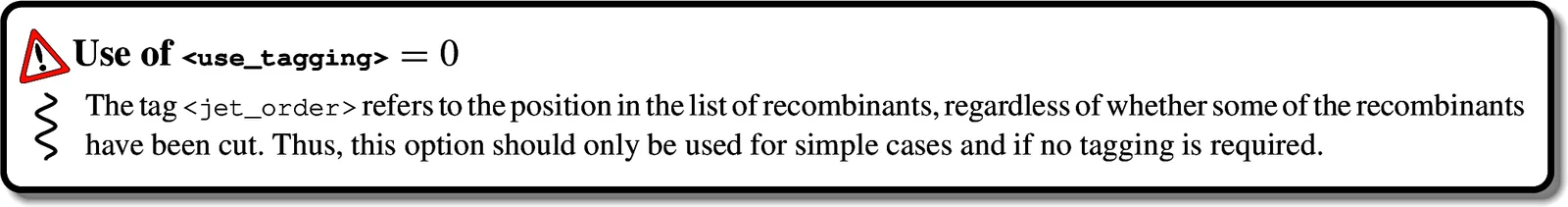

For $$\mathtt {<}  \texttt {use\_tagging}\mathtt {>}$$$${}=1$$ (default), the tagging information of the cut routines is consistently used. Specific jets *must* be tagged and ordered and only ordered jets can be addressed via the tag $$\mathtt {<}  \texttt {jet\_order}\mathtt {>}$$. *This might require the use of extra cuts that take care of the tagging.* For $$\mathtt {<}  \texttt {jet\_order}\mathtt {>}$$$${}>0$$, the jets are taken from the list of *ordered recombinants*, and $$\mathtt {<}  \texttt {jet\_order}\mathtt {>}$$ refers to the corresponding position in this list. For $$\mathtt {<}  \texttt {jet\_order}\mathtt {>}$$$${}=0$$, the jets are taken from the list of *recombinants* after discarding those entries whose tag does not match $$\mathtt {<}  \texttt {jet\_tag}\mathtt {>}$$ or with $$\mathtt {<}  \texttt {jet\_order}\mathtt {>}$$$${}\ne 0$$. For histograms in the single-jet category, all matching jets are taken into account with $$\mathtt {<}  \texttt {jet\_order}\mathtt {>}$$$${}=0$$. This is useful if extra NLO jets, i.e. jets that may or may not be present in the final state, are to be included. For histograms in the multi-jet category, $$\mathtt {<}  \texttt {jet\_order}\mathtt {>}$$$${}=0$$ is not allowed.

For $$\mathtt {<}  \texttt {use\_tagging}\mathtt {>}$$$${}=2$$, the tagging information of the cut routines is used in a different way. If $$\mathtt {<}  \texttt {jet\_tag}\mathtt {>}$$ is missing in the histogram definition, the jets are assumed not to be tagged. Furthermore, all tagged jets must also be ordered. For $$\mathtt {<}  \texttt {jet\_tag}\mathtt {>}$$$${}>0$$, the jets are taken from the list of *ordered recombinants*. For $$\mathtt {<}  \texttt {jet\_tag}\mathtt {>}$$$${}=0$$, the jets are taken from the list of *recombinants* after discarding those entries with non-zero $$\mathtt {<}  \texttt {jet\_tag}\mathtt {>}$$. In both cases, $$\mathtt {<}  \texttt {jet\_order}\mathtt {>}$$ refers to the corresponding entry in this list and not necessarily to the corresponding $$\mathtt {<}  \texttt {jet\_order}\mathtt {>}$$ as defined by the cuts. For histograms that can take lists of jets, all matching jets are taken into account. This scheme allows to access untagged jets in *recombinants* via $$\mathtt {<}  \texttt {jet\_order}\mathtt {>}$$.

A further example is shown in Listing 31, which is understood together with the cuts defined in Listing 24, which define the two tagged jets of the process according to the jet_rapidity cut.

**Figure:**
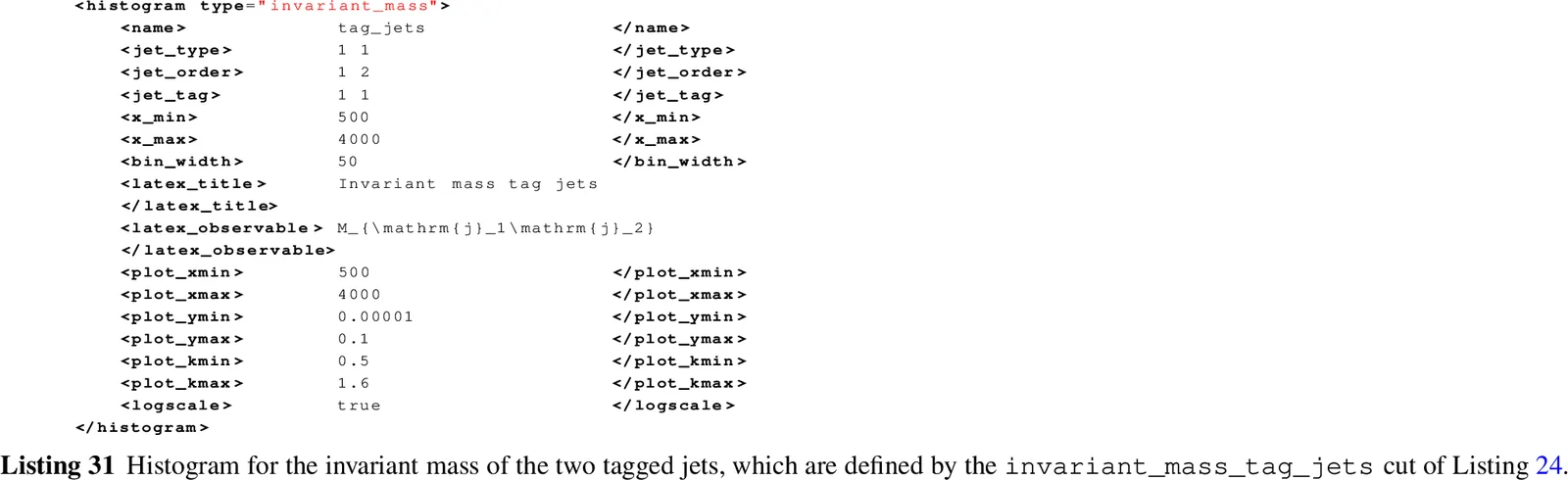

The histogram bins the invariant mass of the two tagged jets, calculated from the first and second momenta ($\mathtt {<}  \texttt {jet\_order}\mathtt {>}$ = 1 2) of the ordered recombinants.

As for cuts and scales, MoCaNLO checks the input for $\mathtt {<}  \texttt {jet\_type}\mathtt {>}$, $\mathtt {<}  \texttt {jet\_tag}\mathtt {>}$, and $\mathtt {<}  \texttt {jet\_order}\mathtt {>}$ also for histograms. By setting the tag

**Figure:**
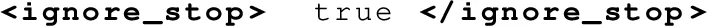

in the $\mathtt {<}  \texttt {histogram}\mathtt {>}$ environment, one can prevent the code from stopping, and if one sets

**Figure:**
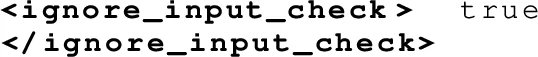

all checks are switched off for the corresponding histogram. This, however, might lead to run-time errors.

## User-defined input features

Despite the general structure and flexibility of the cards, there might still be cuts, scales, or histograms that cannot be obtained from the input in the cards. These objects can be defined in dedicated Fortran files, which are named user_defined_cuts.F90, user_defined_scales.F90, and user_defined_histograms.F90, respectively.

Templates for such files can be found in the folder

**Figure:**
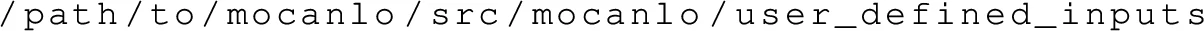

Depending on the user’s needs, one, two, or all of them must be copied from that location to the folder of the process to be computed, and specifically in a subfolder named user_defined. As an example, we consider the process $\text {p} \text {p} \rightarrow \text {e} ^+\text {e} ^-\mu ^-\bar{\nu }_\mu \text {j} \text {j}$ with polarised intermediate bosons, to be found in the folder /path/to/process/vbs/wz/pp_wz_ew_ew_pol. Once the necessary files have been copied, the process directory will look like

**Figure:**
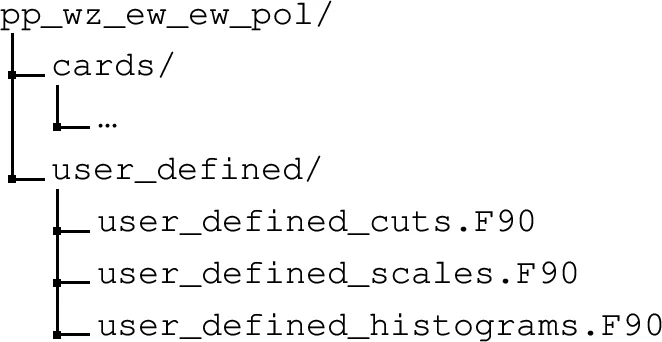

Then, the files must be modified accordingly, as described below. Finally, to link these files when compiling the code, the script compile_mocanlo must be run with the (relative or absolute) path of the process folder as an argument, like

**Figure:**
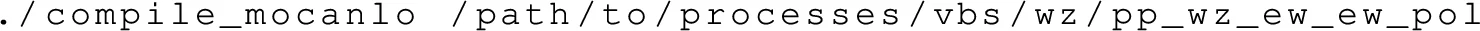

The modification of a user-defined file always requires three steps: specifying the numbers of new features that one wants to implement, setting a template for all of them, and setting up the procedure that contains their actual definitions.

We start showing how a new cut acting on a Zeppenfeld-like variable can be defined for $\text {p} \text {p} \rightarrow \text {e} ^+\text {e} ^-\mu ^-\bar{\nu }_\mu \text {j} \text {j}$ by modifying the file user_defined_cuts.F90. The content of the file is reported in Listing 32.

**Figure:**
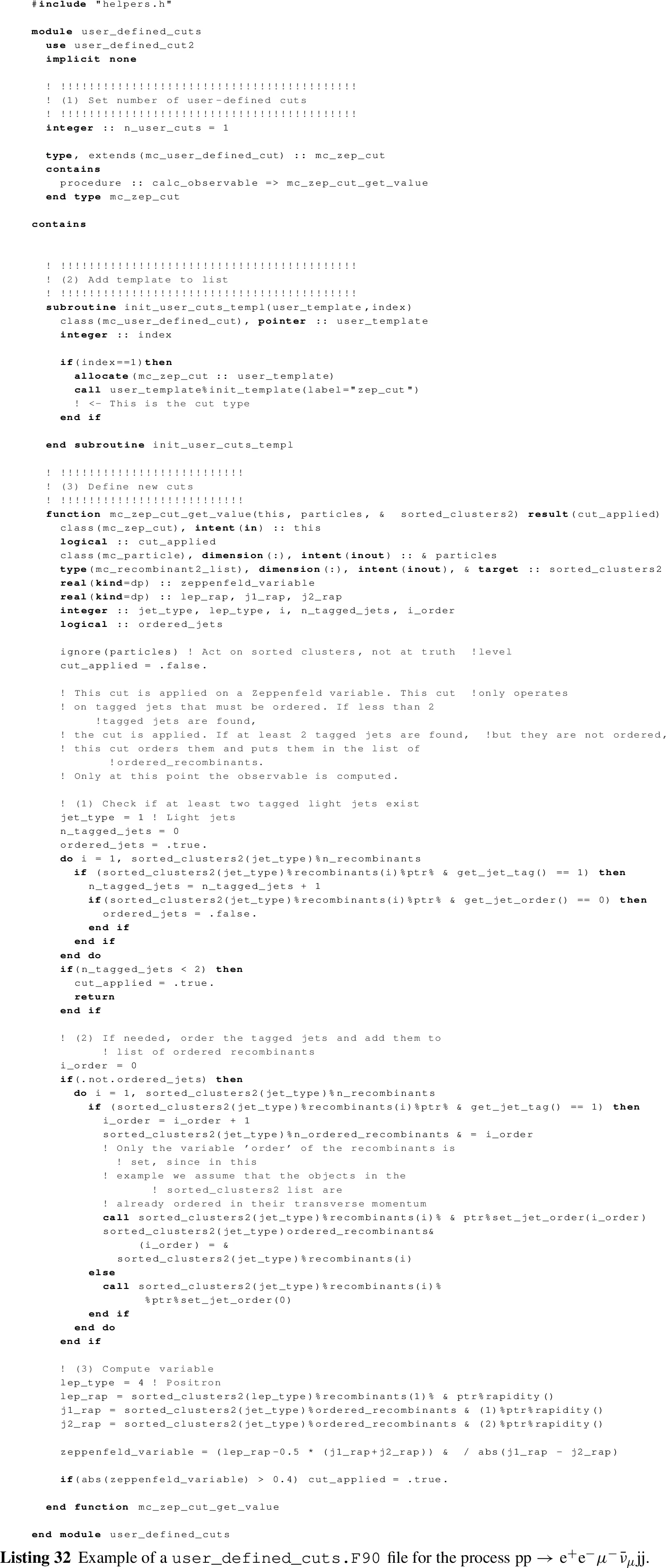

**Figure:**
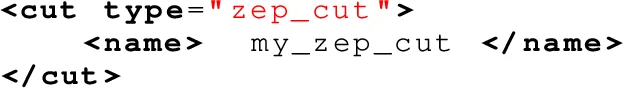

First, the variable n_user_cuts is set to one, i.e. the number of new cuts that we want to add. Right below, a new type mc_zep_cut is declared, inheriting from the type mc_user_defined_cut. The new type just needs to define its own calc_observable procedure, which in the example here is renamed mc_zep_cut_get_value.

As a second step, the if-statement in the subroutine init_user_cuts_templ has to be adapted. Specifically, for each new cut type an else-if scope must be added with the condition index==n, where n is an integer number indexing the different cuts. In the corresponding scope, a template for the cut has to be allocated and initialised. For a user-defined cut, this just requires to set a label. This label defines the type with which the cut becomes accessible from the cut_card.xml:

**Figure:**
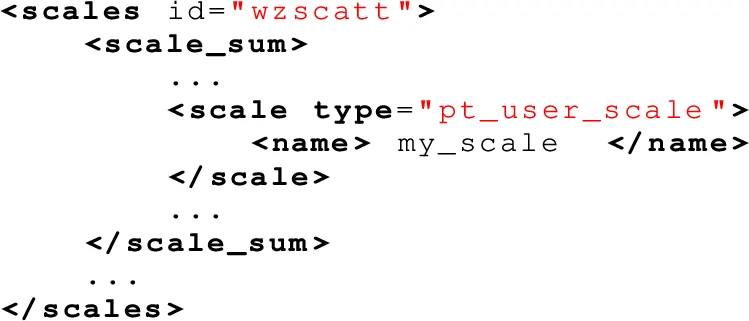

Note that, as for any other cut, the position of the user-defined cut in the sequence of cuts in the cut_card.xml is important.

Finally, the function mc_zep_cut_get_value must be defined. The function is expected to return a logical variable cut_applied that tells whether the cut must be applied or not. Moreover, it always operates on sorted_clusters2, which collects the reconstructed objects for all jet types possibly resulting from the recombination chain and the action of previous cuts. Each of these jet types is a component of the sorted_clusters2 array. Depending on the process, each component can collect multiple objects of the same type. Such objects are accessible via two different lists (for further context, see discussion at the beginning of Sect. 7.3):

- **sorted_clusters2(jet_type)%recombinants(i)%** **ptr** is the list of *recombinants* containing sorted_clusters2(jet_type)%n_recombinants objects of type jet_type. This is the only way of accessing objects if they have not been ordered by the action of a cut via the tag $\mathtt {<}  \texttt {order}\mathtt {>}$. One can inquire whether an object in the list has been tagged or ordered by checking the value of get_jet_tag() and get_jet_order(), respectively.
- **sorted_clusters2(jet_type)%ordered_recombinants(i)%ptr** is the list of *ordered recombinants* containing sorted_clusters2(jet_type)%n_ordered_recombinants objects. This list for a given jet_type is only filled and accessible once the corresponding objects have been ordered by a previous cut. The order of an object corresponds to the position i in the list.

Accessing the member ptr of the recombinant and ordered-recombinant lists allows to perform a set of operations on particle momenta via some pre-defined methods, like computing the transverse momentum or the rapidity using ptr%rapidity() and ptr%transverse_momentum(), respectively. A more comprehensive list of available operations can be found by inspecting the file /path/to/mocanlo/src/mocanlo/math/lorentz.f90.

After this basic introduction, the example reported in Listing 32 can be understood as follows. The user-defined cut first checks if at least two tagged light jets (jet_type==1) exist. If not, the event is cut away. Otherwise, the function mc_zep_cut_get_value proceeds filling the list of ordered_recombinants, in case the jets were not already ordered by a previous cut. Finally, a Zeppenfeld variable among the positron, the leading, and the subleading jet is computed, and the event is cut away if this variable exceeds a hard-coded value.

Defining a new dynamical scale via the user_defined_scales.F90 file requires to go through very similar steps as for the definition of a new cut. The user is expected to introduce a new scale type inheriting from mc_scale having its own calc procedure. As for the cuts, the latter has access to the particle momenta via the list of sorted_clusters2. One should keep in mind that when a scale is computed, the objects collected in sorted_clusters2 are the ones that went through the complete sequence of cuts of cut_card.xml. It is the responsibility of the user to make sure that the objects used in the construction of the scale really exist and are well defined. The actual scale definition must be part of an appropriate calc procedure that returns the scale as a real variable. The user-defined scale can then be used either as an independent scale or as part of a scale_sum definition (see Sect. 7.4). It can be called from the input card like

**Figure:**
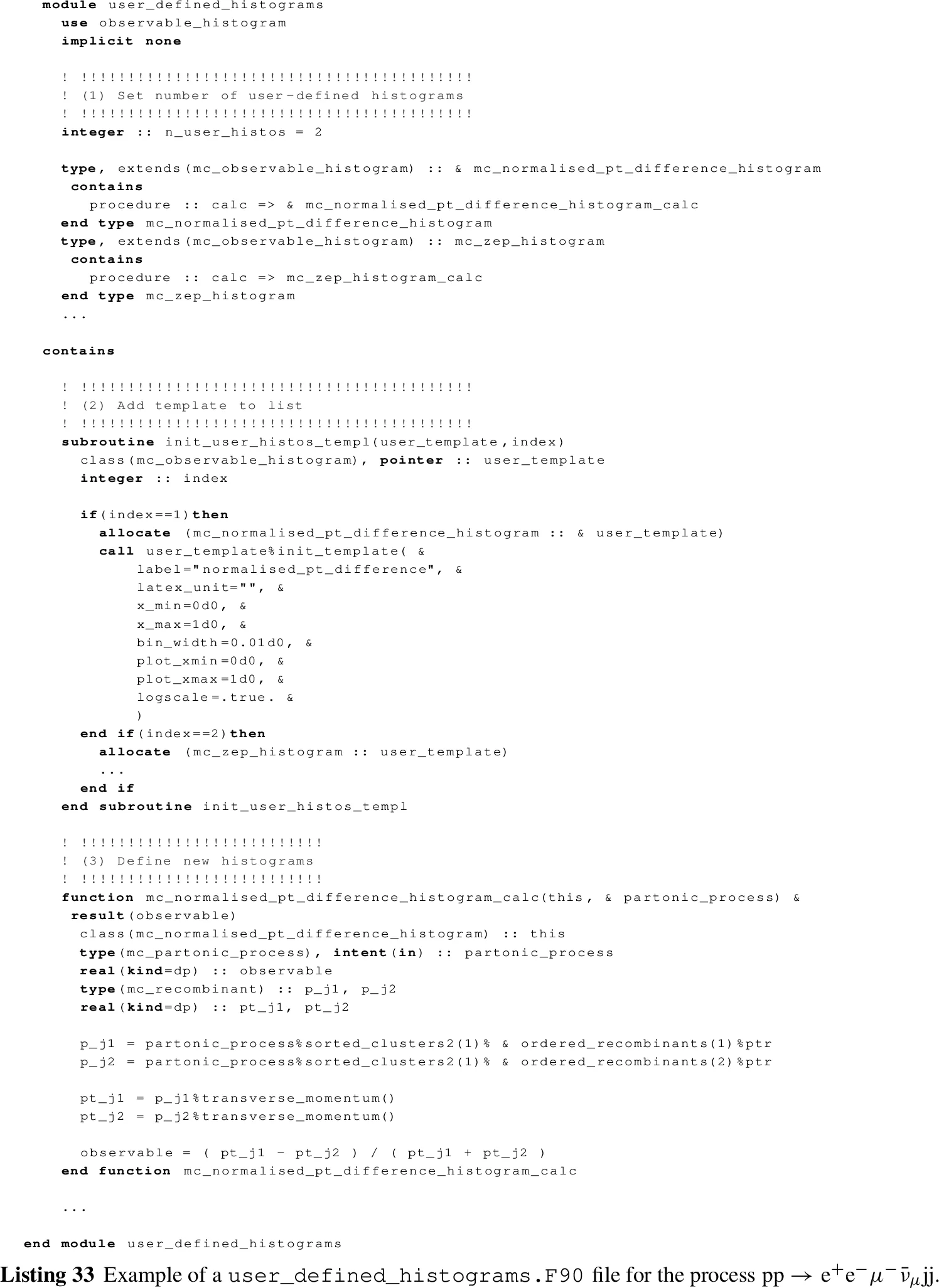

where pt_user_scale is the label used to initialise the scale template.

To introduce new histograms, the workflow is pretty similar up to some small differences that are illustrated in Listing 33.

**Figure:**
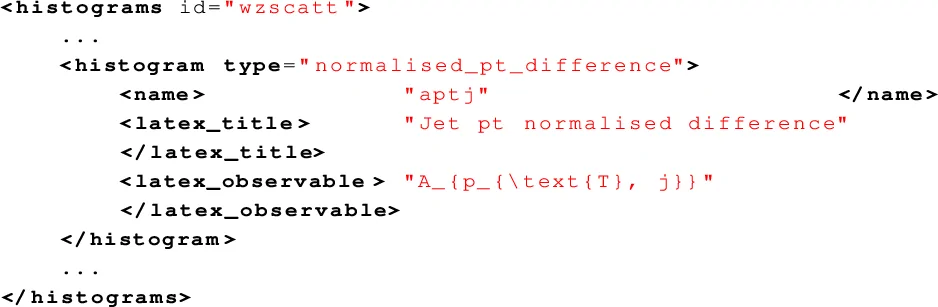

In the example above, two new histograms are introduced. The first thing to note is the definition of the template for the new types. In this case, the template contains a label together with an additional set of entries setting default values (that could always be overwritten from the input card, if needed) for the histogram ranges, binning and so on. The second difference is found in the calc procedure. There, the particle momenta must now be obtained through the partonic_process argument, that in turn provides access to the list of sorted_clusters2, already described above.

Once a histogram is defined, it can be used in the plot_card.xml as any other distribution:

**Figure:**
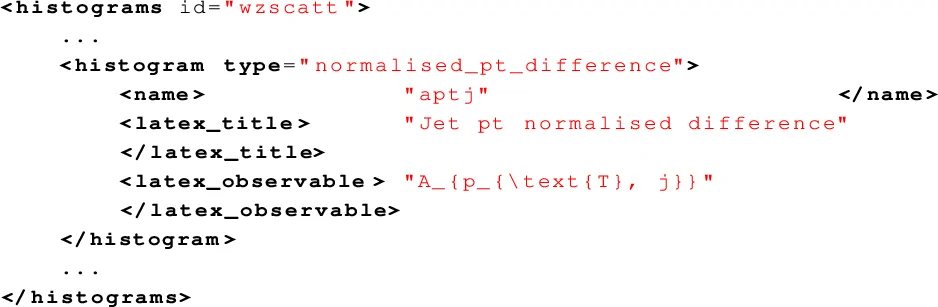

where in the example above the default values for all additional features of the histograms will be used as defined in the allocation of the template for the new type.

## Conclusions

This manual describes the usage of MoCaNLO, a Monte Carlo integration code designed for arbitrary scattering processes at high-energy colliders. It allows the calculation of both NLO QCD and EW corrections as long as external particles that can give rise to real radiation are massless. Non-radiating final-state particles can be massive, and collinear singularities arising from initial-state electrons and muons can be regulated by their masses. For the calculation of tree-level and one-loop matrix elements, the program Recola is used, which in turn relies on Collier for the loop integrals. Infrared singularities are treated with the Catani–Seymour dipole formalism and its extensions, whereby all light fermions are assumed to be massless. Integrated and differential results are obtained upon combining different contributions like the Born, the virtual, the subtracted real and the integrated dipoles for a standard NLO calculation.

MoCaNLO has already been used for a large number of scattering processes at the LHC, including processes with up to eight particles in the final state. It can also deal with processes at lepton–lepton and lepton–proton colliders, as well as with ultra-peripheral collisions of photons in heavy-ion collisions. It supports the helicity selection for intermediate weak bosons and for incoming leptons.

In the future MoCaNLO could be extended in several respects. While MoCaNLO provides results for varied renormalisation and factorisation scales in an efficient way, it would be nice to also evaluate uncertainties from the parton-distributions functions efficiently. The treatment of processes with polarised resonances, which is currently subject to some limitations, is expected to be progressively extended and generalised. Presently, we are working on the matching of MoCaNLO predictions to parton showers via the MC@NLO method. Also, support for 1→n decay processes and 2→1 processes might be considered as an additional feature.

## Data Availability

This manuscript has no associated data. [Author’s’ comment: Data sharing not applicable to this article as no datasets were generated or analysed during the current study.].

## A. Validated processes

This section contains a list of calculations that have been carried out with MoCaNLO, together with the respective references. The corresponding input cards are provided in the subfolders of the folder validated_processes/.

### A.1. Top–antitop production and associated production

1. pp_epvemumvmxbbx_qcd The cards reproduce the calculation of NLO QCD corrections to off-shell tt production in Ref. [98].
2. pp_epvemumvmxbbx_ew The cards have been set up for the calculation of NLO EW corrections for off-shell tt production in Ref. [34].
3. pp_epvemumvmxbbx_ew_DPA_ww The cards have been set up for the calculation of NLO EW corrections for off-shell tt production in Ref. [34] in the WW DPA for the virtual part.
4. pp_epvemumvmxbbx_ew_DPA_tt The cards have been set up for the calculation of NLO EW corrections for off-shell tt production in Ref. [34] in the tt DPA for the virtual part.
5. pp_epvemumvmxbbxh_qcd The cards have been set up for the calculation of NLO QCD corrections for off-shell ttH production in Ref. [35]. Please also cite Ref. [33].
6. pp_epvemumvmxbbxh_ew The cards have been set up for the calculation of NLO EW corrections for off-shell ttH production in Ref. [35].
7. pp_epvemumvmxbbxh_ew_DPA_ww The cards have been set up for the calculation of NLO EW corrections for off-shell ttH production in Ref. [35] in the WW DPA for the virtual part.
8. pp_epvemumvmxbbxh_ew_DPA_tt The cards have been set up for the calculation of NLO EW corrections for off-shell ttH production in Ref. [35] in the tt DPA for the virtual part.
9. pp_ttw The cards have been set up for the calculation of the NLO corrections of orders $\mathcal {O}(\alpha _\text {s} ^3\alpha ^6)$, $\mathcal {O}(\alpha _\text {s} ^2\alpha ^7)$, and $\mathcal {O}(\alpha _\text {s} \alpha ^8)$ to off-shell $\text {t} \overline{\text {t}}\text {W} ^+$ production in the fully leptonic decay channel of Refs. [37, 39].
10. pp_ttz The cards have been set up for the calculation of the full set of LO and NLO corrections for off-shell $\text {t} \overline{\text {t}}\text {Z}$ production in Ref. [40].
11. pp_ttbb The cards have been set up for the calculation of the NLO QCD corrections for off-shell $\text {t} \overline{\text {t}}\text {b} \overline{\text {b}}$ production in Ref. [38].

### A.2. Single-top production

1. pp_tzj
  The cards have been set up for the calculation of NLO QCD and EW corrections to associated tZj production in Ref. [58].

### A.3. Vector-boson scattering

1. pp_ssww_ew_ew
  The cards have been set up for the calculation of $\mathcal {O}\!\left( \alpha ^7 \right)$ corrections to same-sign WW scattering in Ref. [50]. Please also cite Ref. [49] for the $\text {W} ^+\text {W} ^+$ signature.
2. pp_ssww_ew_qcd
  The cards have been set up for the calculation of $\mathcal {O}\!\left( \alpha ^6 \alpha _\text {s} \right)$ corrections to same-sign WW scattering in Ref. [50] for the $\text {W} ^+\text {W} ^+$ signature.
3. pp_ssww_qcd_ew
  The cards have been set up for the calculation of $\mathcal {O}\!\left( \alpha ^5 \alpha _\text {s} ^2 \right)$ corrections to same-sign WW scattering in Ref. [50] for the $\text {W} ^+\text {W} ^+$ signature.
4. pp_ssww_qcd_qcd
  The cards have been set up for the calculation of $\mathcal {O}\!\left( \alpha ^4 \alpha _\text {s} ^3 \right)$ corrections to same-sign WW scattering in Ref. [50] for the $\text {W} ^+\text {W} ^+$ signature.
5. pp_ssww_ew_ew_minus_minus
  The cards have been set up for the calculation of $\mathcal {O}\!\left( \alpha ^7 \right)$ corrections to same-sign WW scattering in Ref. [50] for the $\text {W} ^-\text {W} ^-$ signature. Please also cite Refs. [49, 53].
6. pp_ssww_ew_qcd_minus_minus
  The cards have been set up for the calculation of $\mathcal {O}\!\left( \alpha ^6 \alpha _\text {s} \right)$ corrections to same-sign WW scattering in Ref. [50] for the $\text {W} ^-\text {W} ^-$ signature.
7. pp_ssww_ew_ew_pol
  The cards have been set up for the calculation of $\mathcal {O}\!\left( \alpha ^7 \right)$ corrections to polarised $\text {W} ^+\text {W} ^+$ scattering in Ref. [65].
8. pp_wz_ew_ew
  The cards have been set up for the calculation of $\mathcal {O}\!\left( \alpha ^7 \right)$ corrections to $\text {W} ^+\text {Z}$ scattering in Ref. [51].
9. pp_wz_ew_qcd
  The cards have been set up for the calculation of $\mathcal {O}\!\left( \alpha ^6 \alpha _\text {s} \right)$ corrections to $\text {W} ^+\text {Z}$ scattering in Ref. [51].
10. pp_wz_ew_ew_minus
  The cards have been set up for the calculation of $\mathcal {O}\!\left( \alpha ^7 \right)$ corrections to $\text {W} ^-\text {Z}$ scattering in Ref. [51].
11. pp_wz_ew_qcd_minus
  The cards have been set up for the calculation of $\mathcal {O}\!\left( \alpha ^6 \alpha _\text {s} \right)$ corrections to $\text {W} ^-\text {Z}$ scattering in Ref. [51].
12. pp_wz_ew_ew_pol
  The cards have been set up for the calculation of $\mathcal {O}\!\left( \alpha ^7 \right)$ corrections to polarised $\text {W} ^+\text {Z}$ scattering in Ref. [69].
13. pp_zz_ew_ew
  The cards have been set up for the calculation of $\mathcal {O}\!\left( \alpha ^7 \right)$ corrections to ZZ scattering in Ref. [55]. Please also cite Ref. [54].
14. pp_zz_ew_qcd
  The cards have been set up for the calculation of $\mathcal {O}\!\left( \alpha ^6 \alpha _\text {s} \right)$ corrections to ZZ scattering in Ref. [55]. Please also cite Ref. [54].
15. pp_zz_qcd_ew
  The cards have been set up for the calculation of $\mathcal {O}\!\left( \alpha ^5 \alpha _\text {s} ^2 \right)$ corrections to ZZ scattering in Ref. [55].
16. pp_zz_qcd_qcd
  The cards have been set up for the calculation of $\mathcal {O}\!\left( \alpha ^4 \alpha _\text {s} ^3 \right)$ corrections to ZZ scattering in Ref. [55].
17. pp_wpwm_ew_ew
  The cards have been set up for the calculation of $\mathcal {O}\!\left( \alpha ^7 \right)$ corrections to $\text {W} ^+\text {W} ^-$ scattering in Ref. [56].
18. pp_wpwm_ew_qcd
  The cards have been set up for the calculation of $\mathcal {O}\!\left( \alpha ^6 \alpha _\text {s} \right)$ corrections to $\text {W} ^+\text {W} ^-$ scattering in Ref. [56].

### A.4. Triboson production

1. pp_www_ew_ew
  The cards have been set up for the calculation of $\mathcal {O}\!\left( \alpha ^7 \right)$ corrections to WWW production with one W boson decaying hadronically in Ref. [47]. Please also cite Ref. [49].
2. pp_www_ew_qcd
  The cards have been set up for the calculation of $\mathcal {O}\!\left( \alpha ^6 \alpha _\text {s} \right)$ corrections to WWW production with one W boson decaying hadronically in Ref. [47].
3. pp_www_qcd_ew
  The cards have been set up for the calculation of $\mathcal {O}\!\left( \alpha ^5 \alpha _\text {s} ^2 \right)$ corrections to WWW production with one W boson decaying hadronically in Ref. [47].
4. pp_www_qcd_qcd
  The cards have been set up for the calculation of $\mathcal {O}\!\left( \alpha ^4 \alpha _\text {s} ^3 \right)$ corrections to WWW production with one W boson decaying hadronically in Ref. [47].
5. wzv_semi_leptonic
  The folder collects the cards for the different contributions to WVZ production, with V being either a W or a Z boson decaying hadronically, with the setup described in Ref. [48]. The subfolder pp_wvw_ew_ew contains the $\mathcal {O}\!\left( \alpha ^6 \right)$ and the $\mathcal {O}\!\left( \alpha ^7\right)$ corrections, while the subfolder pp_wvw_ew_qcd the $\mathcal {O}\!\left( \alpha ^6 \alpha _\text {s} \right)$ corrections. The LO contributions at $\mathcal {O}\!\left( \alpha ^5 \alpha _\text {s} \right)$ and $\mathcal {O}\!\left( \alpha ^4 \alpha _\text {s} ^2\right)$ can be found in pp_wvw_LO_as1_a5 and pp_wvw_LO_as2_a4, respectively. Cards to reproduce the results for the bottom- and photon-induced channels at the perturbative orders described in Ref. [48] are located in the subfolder pp_wvw_bottoms and pp_wvw_photons, respectively.

### A.5. Diboson production

1. pp_ww_pol
  The cards have been set up for the calculation of NLO EW corrections to inclusive $\text {W} ^+\text {W} ^-$ production at the LHC with polarised bosons in the fully leptonic channel [63]. Please also cite Ref. [59].
2. pp_wz_pol
  The cards have been set up for the calculation of NLO QCD and NLO EW corrections to inclusive $\text {W} ^+\text {Z}$ production at the LHC with polarised bosons in the fully leptonic channel [68]. Please also cite Ref. [60].
3. pp_zz_pol
  The cards have been set up for the calculation of NLO QCD and NLO EW corrections, as well as the loop-induced $\text {g} \text {g}$ contribution, to inclusive $\text {Z} \text {Z}$ production at the LHC with polarised bosons in the fully leptonic channel [66]. Please also cite Ref. [61].

### A.6. Higgs production

1. vbf_h
  It contains the cards of Ref. [46] for Higgs production via vector-boson fusion (VBF) in one of the setups considered in the reference. It provides the NLO QCD and NLO EW corrections in the full computation to VBF in addition to the subleading contributions of orders $\mathcal {O}\!\left( \alpha _\text {s} ^2 \alpha ^2\right)$, $\mathcal {O}\!\left( \alpha _\text {s} ^3 \alpha ^2\right)$, and $\mathcal {O}\!\left( \alpha _\text {s} ^4 \alpha \right)$. Please also cite Ref. [45] when relevant, as indicated in the README files.
2. vbf_hh
  It contains cards for di-Higgs production via VBF from Ref. [45] at NLO QCD and NLO EW. In addition, cards for an inclusive set-up are also available for NLO EW corrections. In particular, these are used by the LHC Higgs Cross Section Working Group as canonical predictions for VBF.

### A.7. Ultra-peripheral collisions

1. tautau
  The cards have been set up for one check of the calculation of EW corrections to tau-pair production in UPC [99], namely on-shell massless tau fermions leptons.

## Code Availability Statement

This manuscript has associated code/software in a data repository. [Author’s comment: The code described in this publication is available in the gitlab repository at https://mocanlo.gitlab.io or alternatively at the Zenodo record https://zenodo.org/records/19829093.]
